# Polymorphic Solid Solutions in Molecular Crystals: Tips, Tricks, and Switches

**DOI:** 10.1021/jacs.3c07105

**Published:** 2023-09-06

**Authors:** Adam Hill, Weronika Kras, Fragkoulis Theodosiou, Monika Wanat, Daniel Lee, Aurora J. Cruz-Cabeza

**Affiliations:** † Department of Chemistry, University of Durham, Lower Mount Joy, South Rd, Durham, DH1 3LE, U.K.; ‡ Department of Chemical Engineering, The University of Manchester, Oxford Road, Manchester, M13 0PL, U.K.; § Faculty of Chemistry, University of Warsaw, Pasteura 1, 02-093 Warsaw, Poland

## Abstract

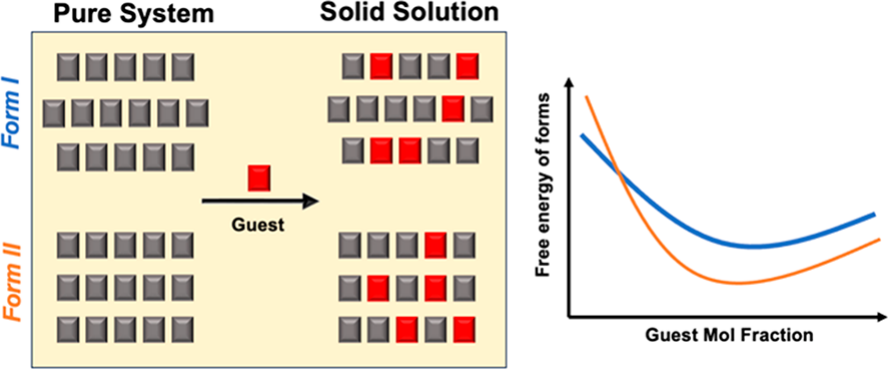

Crystal polymorphism has been a topic of much interest for the past 20 years or so, especially since its scientific (and legal) importance to the pharmaceutical industry was realized. By contrast, the formation of solid solutions in molecular crystals has been overlooked despite its long-standing prevalence in the analogous field of inorganic crystals. Wilfully forgotten, crystalline molecular solid solutions may be very common in our world since molecular compounds are rarely produced with 100% purity, and impurities able to form solid solutions are difficult to reject via recrystallization. Given the importance of both polymorphism and solid solutions in molecular crystals, we share here some tips, tricks, and observations to aid in their understanding. First, we propose a nomenclature system fit for the description of molecular crystalline solid solutions capable of polymorphism (tips). Second, we highlight the challenges associated with their experimental and computational characterization (tricks). Third, we show that our recently reported observation that polymorph stabilities can change by virtue of solid solution formation is a general phenomenon, reporting it on a second system (switches). Our work focuses on the historically important compound benzamide forming solid solutions with nicotinamide and 3-fluorobenzamide.

## 1 Introduction

The physiochemical properties of a crystalline material can be altered by changing the spatial arrangement of its constituents, a phenomenon known as crystal polymorphism. First reported in 1832 for the molecular system benzamide (bzm),^1^ polymorphism is of paramount importance for the chemical, food, and pharmaceutical industries. A common adage repeated in the study of polymorphism is that *“in general, the number of forms known for a compound is proportional to the time and money spent in its research”*.^2^ In some cases, a metastable crystalline form of a compound has more desirable physical properties for a particular application, resulting in a need for experimental methods to obtain metastable polymorphs reliably and efficiently.^3,4^ The spontaneous and unexpected appearance of new more stable and less soluble forms can have significant consequences for drug delivery (as seen in the famous case of Ritonavir).^5^

Beyond polymorphs, crystalline molecular systems can also form solid solutions (SSs).^6^ Analogous to liquid solutions, crystalline SSs contain a major component or components (the “*host*”) and a minor component (the “*guest*”), which is distributed in the crystal lattice in sites otherwise occupied by the host in the pure crystal. When referring to “host” or “guest”, one can refer to the structure of the compound itself (host compound) or the structure of the crystal (host crystal structure). The crystalline host molecules act as “solvent”, while guest molecules act as “solute” within the host crystal structure. SSs can exist in a range of compositions, from small and undetectable molar fractions (*x*_g_ ≈ 0), to equimolar (*x*_g_ = 0.5) or even dominant fractions of the guest (*x*_g_ > 0.5, effectively resulting in a switch of the host:guest roles). The difference between a SS with *x*_g_ = 0.5 and a 1:1 cocrystal is that in the SS, guest and host molecules sit in equivalent lattice sites while in the cocrystal, the different components occupy different lattice sites. Like liquid solutions, some SSs can become saturated thus forming what is known as a *partial* SS.^7^ For these, one can thus find a maximum value of *x*_g_ (*x*_g,sat_) above which no further guest molecules can be incorporated into the lattice of the SS (the solid solubility of the guest in the crystal structure of the SS). In other SSs, all *x*_g_ compositions are possible (from 0 to 1), thus the host:guest system is capable of forming a *continuous* SS. Thermodynamics dictate the incorporation of a guest in the lattice of a host and whether or not the SS is possible across the entire compositional range.^8^ The crystal structure of the SS is usually similar to the structure of the pure host, though this is not always the case, and in certain cases various crystal structures of SSs are possible (analogous to polymorphs). While it is not obvious when a given guest molecule will form a SS with a given host molecule in a specific crystal structure, molecular similarity between the guest and the host is often (though not always) a necessity for the formation of a SS. Early works by Kitaigorodskii and recent works by Lusi et al. have explored this question in more detail, a subject topic which is beyond the scope of the present study.^6,9^ Here we ask a different question in relation to SSs and polymorphism: given a host able to crystallize in different polymorphic forms, what is the effect of SS formation on the host crystal polymorphism?^10−12^

In recent work inspired by the ball mill grinding results of Fischer et al.,^13^ we have shown that the otherwise very elusive form III of bzm can be consistently crystallized from solution in the presence of nicotinamide (ncm). We showed that bzm form I (stable as pure, BZM-I) and bzm form III (metastable as pure, BZM-III) switch thermodynamic stabilities upon SS formation with ncm and that such a switch occurs at *x*_ncm_ ≥ 0.03. The study of SS formation with modeling and experimental approaches is in its infancy, with only a small pool of articles investigating their formation with two approaches.^14−20^ Our previous work lies in an even smaller pool of studies, demonstrating that SS formation can result in a switch of thermodynamic stabilities of crystal forms.^21−23^ In the present work, our intention is to demonstrate that our previous observation is common and that polymorph stability switches in SSs can occur in other systems. Here we show that the thermodynamic stability switch between BZM-I and BZM-III can also be achieved with a different guest: 3-fluorobenzamide (3fbzm). For this system, however, our computer simulations as performed in our previous work fell short in reproducing the experimental findings. After much work to improve our experimental characterization and simulations of the SSs, we found that consideration of the conformational disorder of the *guest* molecules, as well as other entropic contributions in the calculations, is a necessity for the correct stability computation of polymorphic SSs. The results are encouraging since with these complex calculations we can now derive very accurate phase diagrams for the SS systems, but they are also inconvenient since they confirm the necessity for complex and computationally expensive simulations to achieve the correct modeling.

**Figure 1:**
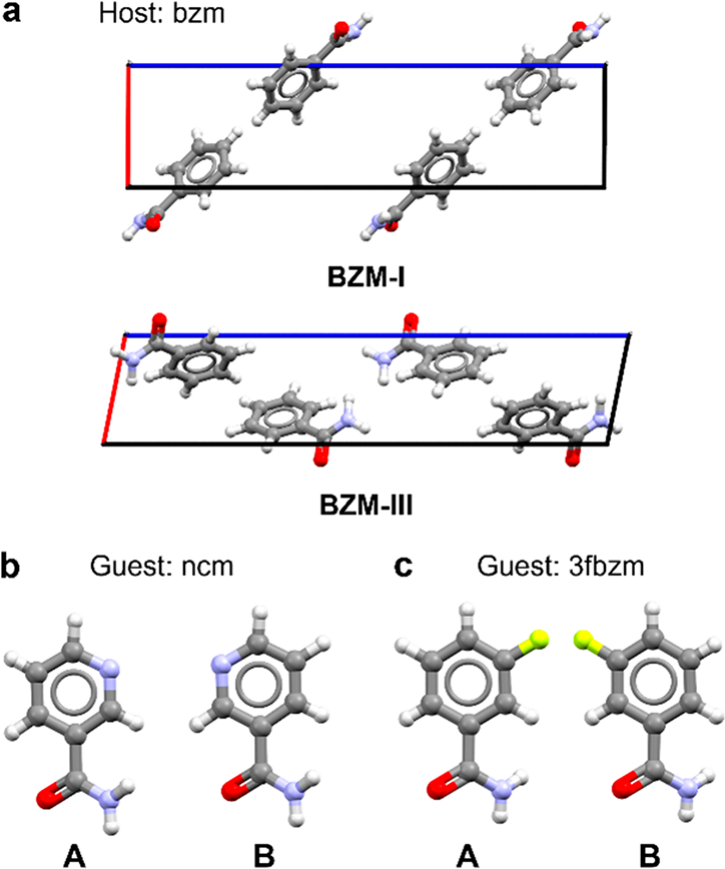
Systems studied in this work: bzm is the host compound crystallized in its forms I and III polymorphs (a) and ncm (b) and 3fbzm (c) are the guests. Both guests can adopt two distinct conformers (A and B).

In this article, we first introduce appropriate general nomenclature for dealing with these multicomponent polymorphic systems (tips). We then demonstrate techniques for accurately identifying and characterizing polymorphic SSs experimentally, along with methods to simulate them computationally (tricks). Alongside the discussion of this two-pronged approach, we demonstrate its applicability supported by results from our host compound, bzm, crystallized in its BZM-I and BZM-III polymorphs in the presence of two different guests: ncm and 3fbzm (Figure 1). Finally, we show that polymorph stability switches in SSs are general with detailed discussion of results from our model systems and then seek to expand the underlying concepts to molecular solids as a whole (switches).

## 2 Results and Discussion

### 2.1 Tips

In this section we present nomenclature “tips” for concise and meaningful descriptions of SS systems. Robust systems of nomenclature and terminology are crucial for discussing SSs, as interpretations of their compositions and structures can vary widely in scientific discussion.

#### 2.1.1 Naming and Abbreviations for Solid Solutions

Our systems here can vary in molecular composition and crystal structure. As such, it is important that our abbreviations distinguish between compounds and crystal structures. For clarity, when referring to compounds lower case abbreviations are used, while when referring to crystal structures upper case abbreviations followed by roman numerals denoting the form are used. For example, bzm and 3fbzm are used to refer to compounds benzamide and 3-fluorobenzamide, respectively. In contrast, BZM-I, BZM-III, and 3FBZM-I are used to refer to the crystal structures of form I of bzm, form III of bzm, and form I of 3fbzm, respectively.

Here we propose an efficient and clear abbreviation system for describing molecular SSs. For this, we state the crystal structure of the SS with its composition in brackets. For example, BZM-III [bzm_0.75_:3fbzm_0.25_] refers to a SS adopting the crystal structure of BZM-III, containing the two components bzm and 3fbzm. The subscripts indicate the mole fractions of each of the components within the lattice. In this case, bzm is the host compound and 3fbzm is the guest since the former is the major component. This is also reflected in the order of the molecular components in the formula.

Cases will exist where the crystal structure of the pure host is identical to the crystal structure of the pure guest. This is true for bzm and 3fbzm, with BZM-III being isostructural to 3FBZM-I. In such a case, BZM-III [bzm_0.75_:3fbzm_0.25_] and 3FBZM-I [bzm_0.75_:3fbzm_0.25_] would be equally accepted abbreviations for the same SS, though the former nomenclature should be preferred because the host (major component) at the reported composition is bzm.

In cases where components can exist in two or more configurations or conformations, we can also refer to the configurational composition by adding an extra subscript denoting the mole fraction of the A–B–C–M··· configurations possible. While this may not always be known or determined experimentally, these considerations are of importance for the modeling of SSs. Hence in BZM-III [bzm_0.75_:3fbzm_0.08,A_:3fbzm_0.17,B_], 3fbzm exists in a 0.25 mole fraction and it can be orientated in two configurations A and B, with B being the major and A the minor configuration.

#### 2.1.2 Relationships between Solid Solutions and Polymorphism

A crystalline SS is a crystal form that contains at least two different components in its structure, a host and a guest, whose compositions can vary continuously within some compositional limits. As detailed in the introduction, the guest compound is “dissolved” in the crystal lattice of the host. To fully describe a SS, it is therefore important to establish the molecular nature of the host and guest structures as well as the crystal structure adopted by the SS.

Polymorphs are defined as different crystal structures adopted by the same compound (or compounds) with an unchanged composition. McCrone’s definition is illustrative here: “polymorphs have different crystal structures but are identical in the liquid or vapour states”.^2^ A SS host:guest system at a fixed composition can indeed be classed as polymorphic if multiple crystal structures exist for such a system at such fixed composition.

**Figure 2:**
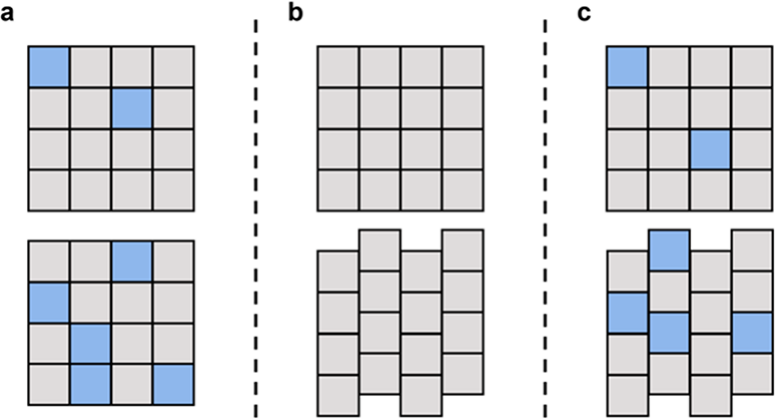
Illustrative diagrams of (a) isostructural SSs with different host:guest concentrations, (b) polymorphic crystal structures, and (c) polymorphic SSs at different concentrations. Blue and gray squares denote chemically distinct crystalline species. Figure design inspired by M. Lusi – ref (8).

In the confluence of polymorphism and SSs, the question then arises as to how we shall refer to those systems when there is a change in the crystal structure as well as a change in the relative composition of constituent components. Such systems are not “pure” polymorphs per se, according to McCrone’s definition. They are SSs, and thus can change the concentration of host:guest, and they can also adopt different crystal structures, therefore being polymorphic. As such, the term *polymorphic SSs at different concentrations* should be appropriate. Structural differences between SSs, polymorphs, and polymorphic SSs are illustrated in Figure 2.

#### 2.1.3 Nomenclature and Relationship Examples

Given the above definitions, we have compiled examples of pairs of crystal structures and their relationships in Table 1. When there is a change of crystal structure but no further change in its component or relative component composition, those systems are pure polymorphs (examples 1 and 2). When there is a change in the relative composition of components within an SS, this is simply a change in concentration of the SS (examples 4 and 5) and the crystal structure relationship should indicate whether the pairs are isostructural or polymorphic. These definitions also apply to SSs with species present in different conformations, with the additional information on the conformer ratio provided when required (examples 6 and 7). Finally, the same nomenclature can be used when all host molecules in a host crystal structure are replaced by guest molecules to produce an isostructural polymorph of the guest compound (example 7) and even for describing conformer disorder in a single component system (example 7) or solid solutions of other systems such as hydrates, solvates, cocrystals, or inclusion compounds. For this, all that is needed is an abbreviation for the crystal structure of the main component with all components and their compositions given inside the brackets. We have shown this for solid solutions of single component systems, and this can be expanded to multicomponent systems, but this is beyond the scope of the current article.

**Table 1:** Examples of Relationships among a Number of Crystal Structure Pairs

**Example**	**CrystalStructure I**	**CrystalStructure II**	**Relationship**
**#1**	BZM-I	BZM-III	Polymorphs
**#2**	BZM-I [bzm_0.75_:3fbzm_0.25_]	BZM-III [bzm_0.75_:3fbzm_0.25_]	Polymorphs
**#3**	BZM-I	BZM-I [bzm_0.75_:3fbzm_0.25_]	Pure form with isostructural SS
**#4**	BZM-I [bzm_0.75_:3fbzm_0.25_]	BZM-I [bzm_0.6_:3fbzm_0.4_]	Isostructural SSs at different concentrations
**#5**	BZM-I [bzm_0.75_:3fbzm_0.25_]	BZM-III [bzm_0.6_:3fbzm_0.4_]	Polymorphic SSs at different concentrations
**#6**	BZM-I [bzm_0.75_:3fbzm_0.20,A_:3fbzm_0.05,B_]	BZM-III [bzm_0.6_:3fbzm_0.2,A_:3fbzm_0.4,B_]	Polymorphic SSs at different concentrations with different guest conformer ratios
**#7**	BZM-III	BZM-III [3fbzm_0.3,A_:3fbzm_0.7,B_]	Isostructural forms for pure host and pure guest with a specific ratio of guest conformers

#### 2.1.4 Terminology for Host:Guest Concentrations

Due to the in-depth discussion of relative quantities of host and guest species that can differ in meaning and value dependent on experimental parameters of synthesis and characterization, care has been taken to develop a clear set of terms for referring to their respective mole fractions. The nomenclature is developed so that one is able to refer to the synthetic route for SS formation, although the solvent composition in the overall synthetic route (if applicable) is often obviated. Using the symbol *x*, as is commonly used to refer to mole fractions, a series of superscripts and subscripts are applied for different purposes. When referring to mole fractions of a general host, the subscript h is used (*x*_h_), while g is used to denote mole fractions of a general guest (*x*_g_). These general subscripts are replaced by abbreviations denoting the compounds present when discussing real examples of SSs (bzm, ncm, and 3fbzm). For systems in which conformational disorder is of importance, the subscript can be modified to represent conformers by following the compound with a comma, and the letter assigned to the specific conformer or configuration. Superscripts are reserved for the experimental environment in which the mole fraction is measured; for example, the quantity of substance added to a slurry solution is denoted with “slurry”, while the equivalent for liquid assisted grinding (LAG) is denoted “LAG” and physical mixtures of species are denoted “PM”. When discussing the incorporation of species into the SS structure, the superscript “SS” is used. To summarize, *x*_h_^slurry^, *x*_h_^LAG^, *x*_h_^PM^, and *x*_h_^SS^ refer to the mole fraction of host initially added to the slurry, LAG or physical mixture, and the mole fraction of host present in the SS, respectively. When guest species are referenced, *x*_g_^slurry^, *x*_g_^LAG^, *x*_g_^PM^, and *x*_g_^SS^ are equivalent terms. The term *x*_g_^SS^ can be modified to *x*_g,*A*_^SS^ when referring to a specific molecular conformer (in this case, conformer A) which can only be measured with confidence after SS incorporation. This system of superscripts and subscripts will be used for all terms presented later in the manuscript.

Some specific mole fractions of guests incorporated into an SS lattice are important and are assigned unique labels. For example, the mole fraction of guest required to cause a polymorphic stability switch in a SS can be denoted as *x*_g,switch_^SS^. This term can be modified in scenarios where there are multiple examples of host structure stability switches across the *x*_g_^SS^ range to *x*_g,switch1_^SS^ and *x*_g,switch2_^SS^ and so on. *x*_g,min_^SS^ and *x*_g,sat_^SS^ denote the mole fraction of guest which results in the lowest energy SS across the system and the point at which the SS becomes saturated (for partial solid solutions only) respectively.

### 2.2 Tricks

In this section, we present general experimental “tricks” for preparing and characterizing SSs, along with a computational approach for modeling their energetics. Combination of both approaches can give a detailed insight into the properties of SSs which can vary extensively depending on their structure and behavior across guest concentration ranges.

#### 2.2.1 Experimental Preparation and Characterization of Solid Solutions

**Figure 3:**
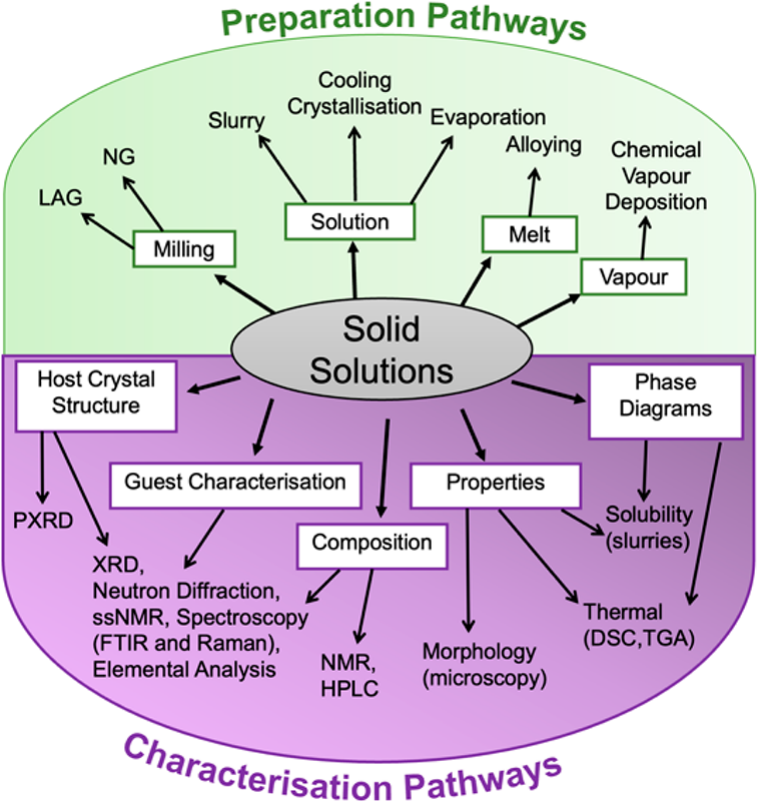
A summary of general experimental pathways in SS formation and characterization.

SSs can be formed in several ways: milling, from solution, through melting and via vapor deposition.^24−27^ Once formed, due to their disordered nature and typically minor amount of guest incorporation, their identification and characterization can be challenging. A summary of SS formation and characterization techniques is presented in Figure 3. If obtained in a solid crystalline form, for example, from milling, the first and usually quickest way of characterization would be via powder X-ray diffraction (PXRD). This would however give a diffraction pattern corresponding to the host structure; the trick here is to compare the PXRD patterns at various guest concentrations and analyze shifts of diffraction peaks as a function of guest concentration. These peak shifts would occur in a SS due to the guest incorporation in the host’s crystal structure, impacting on *d*-spacings. If suitable single crystals can be obtained from solution crystallization, methods such as single crystal X-ray diffraction and neutron diffraction can be utilized to characterize the host’s crystal structure and guest’s molecular structure and incorporation.

Another important factor to consider in SS characterization is the quantification of guest incorporation (*x*_g_^SS^). This would usually depend on the synthetic method used, with *x*_g_^SS^ being typically equal to the amount used in a LAG milling experiment (*x*_g_^SS^ = *x*_g_^LAG^) if *x*_g_^LAG^ < *x*_g,sat_^SS^, and thus no diffraction peaks of the guest structure should be observed. However, with solution crystallization, *x*_g_^SS^ is expected to differ from the amount initially added in the crystallization experiment (i.e., some of the guest will remain in the mother liquor and will not incorporate in the host crystal structure), and thus *x*_g_^SS^ needs to be quantified in the solid phase after the SS has been crystallized. In Figure 3 we suggest several characterization techniques to conduct SS guest quantification NMR, HPLC, and spectroscopy, though other methods are also possible. The decision of which one to use would significantly depend on the differences in properties of the guest and the host molecules.

Lastly, one cannot overlook the importance of analyzing the physical properties and constructing phase diagrams of SSs, especially for polymorphic SSs. Thermal analysis via differential scanning calorimetry (DSC) provides further evidence of SS formation through changes in curve profiles denoting melting events, as well as changes in measured melting temperatures as a function of *x*_g_. SSs, being homogeneous phases, would have a characteristic single melting event and would usually display a continuous melting temperature relationship with changing *x*_g_. An exception to this behavior is seeing when a SS is “ideal”, in which case the melting temperature remains constant with increasing guest concentration.^28^

#### 2.2.2 Computational Modeling of Solid Solutions

The modeling of SSs is notoriously tricky. On one hand, highly accurate energy models are required to be able to model molecular crystals. On the other hand, SSs are inherently disordered. First, there is positional disorder of a guest compound in a host lattice, and second, conformational disorder may also exist due to the possibility of the guest and/or host to exist in such a SS in several conformations. Because disorder is inherently aperiodic, modeling of these systems tends to require ensemble approaches and the utilization of large supercells.^18^

**Figure 4:**
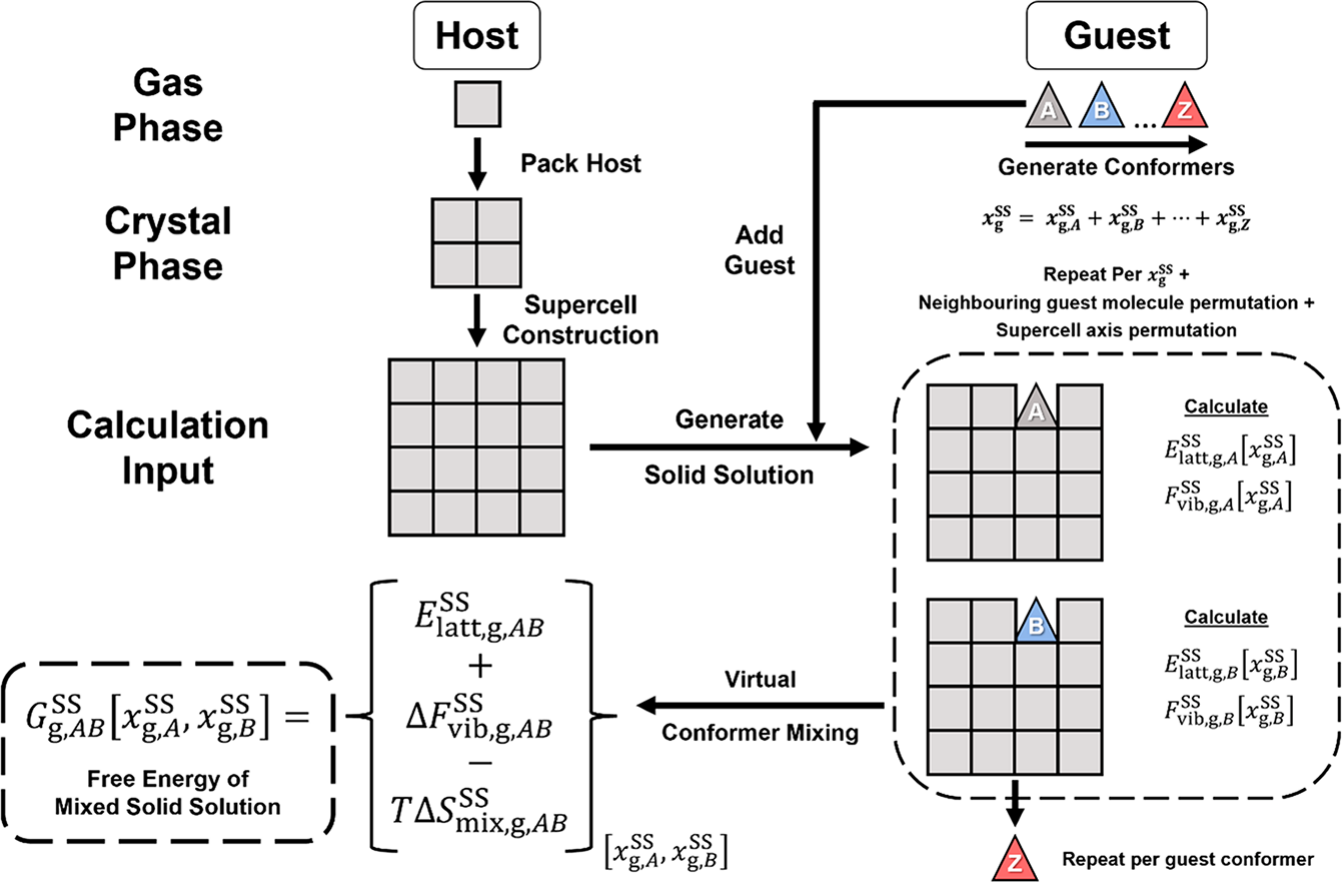
A flow diagram presenting the simulation approach to obtain free energies for conformationally disordered SS, starting from a pure host crystal structure.

A general workflow to obtain accurate free energies for conformationally disordered SSs is presented in Figure 4. Only conformational disorder in the guest molecule is considered in this example; however, the process can be adapted for a conformationally disordered host or for conformational disorder in both host and guest molecules. Proceeding through the workflow proceeds in order, reference states for the isolated host and guest molecules in the gas phase must first be computed. This is performed by optimizing a single molecule in a supercell of sufficient size to avoid self-interaction of molecules in adjacent cells. All major conformers of the guest molecule denoted A through Z are generated and optimized in the gas phase to find the lowest energy conformer to use as the reference state. Moving to the solid phase, lattice energies (*E*_latt_) for the stable crystal forms of both host and guest are computed. This is also conducted for metastable forms of the host if polymorphic switches are to be investigated. The pure host structures are necessary to be used as *E*_latt_ at *x*_g_^SS^ = 0, while the pure guest *E*_latt_ is required to plot the energy of the physical mixture between the pure host and pure guest (along with mixtures of any SS and excess host or guest).

Using a bespoke Python code and the CSD Python API, SS cells are then constructed where host molecules are replaced by overlaid guest molecules in the host crystal structure (stable or metastable forms), with the cell generation repeated for each *x*_g_^SS^ level required. Depending on the number of molecules present in a single unit cell of the host structure, different *x*_g_^SS^ values can be achieved through molecule replacement. If the desired *x*_g_^SS^ requires multiple host molecules within a single cell to be replaced by guest molecules, then SS cells are generated with all possible permutations of neighboring guest molecules for comparison. For the opposing case where the *x*_g_^SS^ value is too low to be reached by replacing molecules within a single unit cell, then SS supercells must be constructed. This supercell is sized so that replacing a single host molecule with a guest molecule produces the desired *x*_g_^SS^. Different supercell axis permutations producing the same *x*_g_^SS^ are also sampled (e.g., 2 × 4 × 1 and 4 × 2 × 1 cells). For each of the SS supercells generated, a copy is also generated with each known conformer of the guest molecule used as a substitute.

Once the SS cells have been generated, they are optimized with VASP (or other equivalent periodic DFT software), and the *E*_latt_ for a SS at a specific *x*_g_^SS^ value (*E*_latt,g_^SS^[*x*_g_^SS^]) is calculated using the reference states of host and guest. This parameter is calculated for all guest conformer-substituted SS crystal structures at each accessible value for *x*_g_^SS^. Optimized SS cells are then used to compute the vibrational contributions to the free energy (*F*_vib_). As far as the authors are aware, this is the first application of this methodology to molecular SSs. These calculations were performed by first expanding optimized cells into supercells with cell parameters of at least 10 Å (as recommended in the literature).^29^ The finite-difference approach within VASP is then used to calculate the *F*_vib_ for a particular *x*_g_^SS^, denoted *F*_vib,g_^SS^[*x*_g_^SS^] in the workflow. This parameter is calculated alongside *E*_latt,g_^SS^[*x*_g_^SS^] for all cells listed previously. The mean *E*_latt,g_^SS^[*x*_g_^SS^] and *F*_vib,g_^SS^[*x*_g_^SS^] can be taken from all cells simulated at the same *x*_g_^SS^ to be used for further calculations to account for disorder.

Moving to the final stage of the workflow, once the energetic parameters are calculated for the SS cells, mixing effects must be considered. SSs are mixed solids, and therefore neglecting these terms can unfairly penalize their stability versus pure solids. As computing all combinations of mixed guest molecules and conformers in host cells at this level of theory would be prohibitively expensive, some simplifications are necessary. First, the mixing effect on enthalpy due to guest conformers is considered by calculating a mixed *E*_latt,g_^SS^[*x*_g_^SS^], comprised of a weighted average of *E*_latt,g_^SS^[*x*_g_^SS^] from each guest conformer substituted SS cell. Second the mixing effect on the *F*_vib,g_^SS^[*x*_g_^SS^] due to guest conformers is computed in the same manner; however, the *F*_vib,g_^SS^[*x*_g_^SS^] is first converted to a relative term Δ*F*_vib,g_^SS^[*x*_g_^SS^] (by subtracting *F*_vib,g_^SS^ from a consistent reference cell for each *x*_g_^SS^). This is necessary to avoid computation of *F*_vib_ for a molecule in the gas phase and thus requires only the simulation of vibrational modes in the solid phase. Finally, the mixing effect of host and guest on the entropy is also considered by calculating Δ*S*_mix,g_^SS^ using eq 1 (assuming ideal mixing of guest molecules in the host crystal structure).
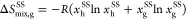
1*R* denotes the gas constant, and the mole fractions of host and guest are denoted following the conventions shown in the Tips section of this manuscript. Should the guest (or the host) exist in different conformations, each conformation will be treated with an independent mixing term. For example, a SS with a guest able to be included into two different conformations will have three terms for the entropy of mixing (the host, guest A, and guest B terms).

The final free energy of the system (*G*_g_^SS^) is then computed by adding the three host:guest terms, as in eq 2, which are all dependent on the host:guest concentration. The free energy can then be plotted over the entire concentration range of *x*_g_^SS^ for all desired host crystal structures, and conformational disorder of the host and/or guest is also considered if necessary.
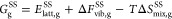
2

## 3 Switches

In this section, we apply our tricks for the generation and characterization of SSs of bzm with two guest compounds: (i) ncm and (ii) 3fbzm. Following on our previously reported observation that polymorphs can switch in thermodynamic stability through the incorporation of impurities for the bzm:ncm system,^23^ we extend our work to a second guest (3fbzm) for which similar observations are seen. We compare both systems side-by-side to illustrate the different nature of the SSs forming and that a switch in phase stabilities as a function of guest concentration is a common phenomenon.

### 3.1 Introduction to the Benzamide System

As the first molecular compound reported to be polymorphic (by Wöhler and Liebig in 1832^1^), bzm plays a significant role in the history of polymorphism. The structure of its stable form I (BZM-I) was characterized in 1959,^30^ and other metastable forms reported thus far are BZM-II, BZM-III, and the recently discovered BZM-IV.^31−33^ It is notoriously difficult to crystallize and characterize the metastable forms of bzm since they are very elusive, and they often crystallize concomitantly with BZM-I. In our recent paper, we have shown that good quality single crystals of BZM-III can be crystallized exclusively in the presence of ncm. This observation was ascribed to the fact that, through SS formation with ncm, BZM-III becomes the most stable polymorph.^23^ Here, we revised our characterization and modeling of this bzm system with ncm and expand the work with a new guest, 3fbzm.

### 3.2 Exploring Solid Solution Formation in Benzamide with LAG

**Figure 5:**
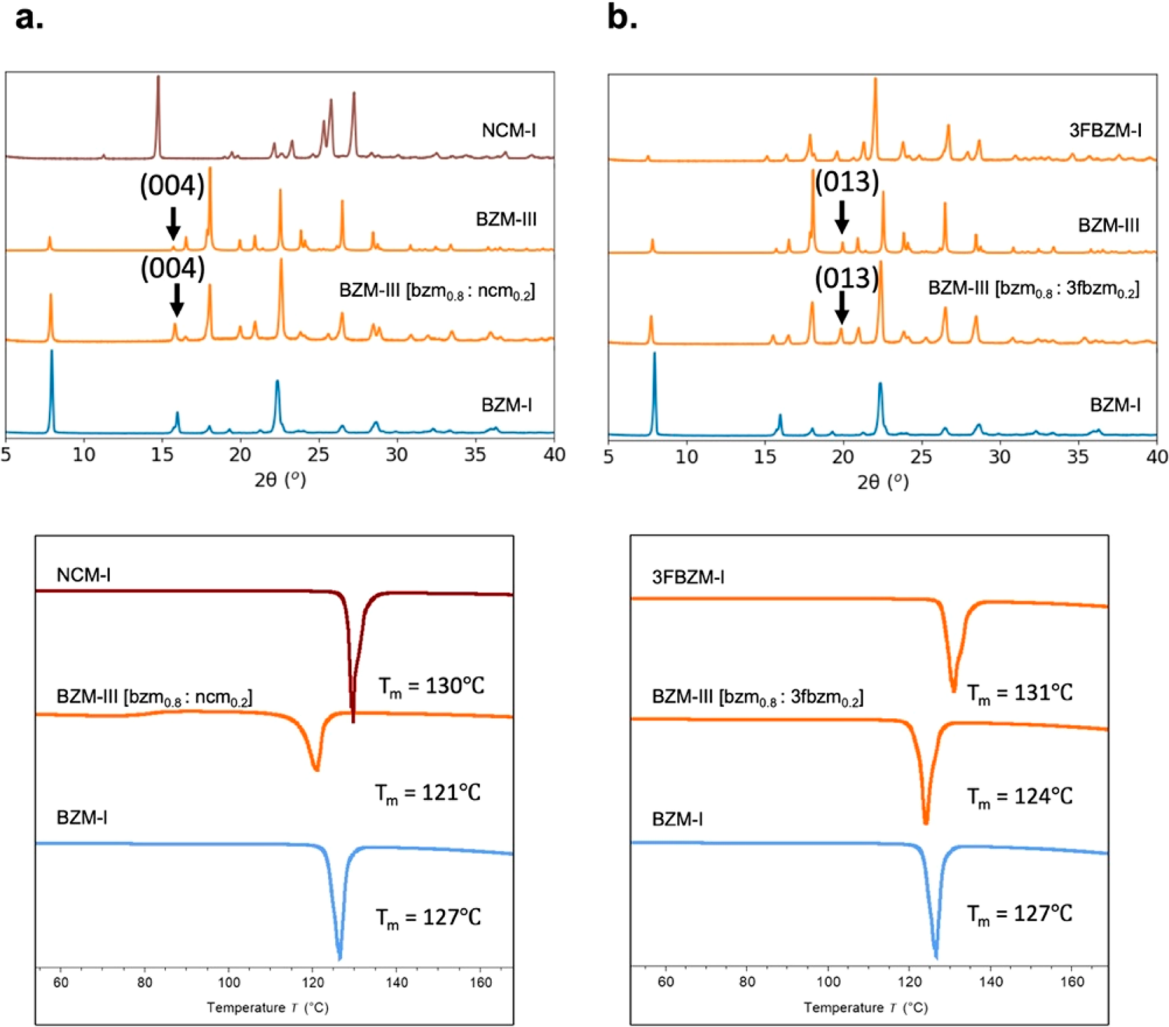
Characterization of LAG crystals via PXRD (top) and DSC (bottom, endotherms down) for (a) bzm:ncm and (b) bzm:3fbzm systems. *x*_g_^LAG^ is approximately 0.2. The labeled peaks were found to have the clearest peak shifts in the 2θ range of 5° to 22° (their shift behavior is shown in Figure 6). Lines are colored by the crystal structure adopted by the sample: blue – BZM-I, orange – BZM-III or 3FBZM-I, and brown/red – NCM-I.

We began assessing SS formation with bzm by conducting LAG experiments with isopropanol (IPA). Previous mechanochemistry studies on bzm indicated that while LAG of pure BZM-I in IPA affords BZM-I, as soon as small amounts of ncm are added to the grinding jar or in neat grinding, BZM-III is obtained.^13,23^ Milling 3FBZM-I in the range of 0.04 ≤ *x*_g_^LAG^ ≤ 1.00 with BZM-I and 1 μL mg^–1^ of IPA for 1 h allowed for efficient initial insights into the possibility of SS formation. The milled materials were then characterized with PXRD and DSC (Figure 5).

As shown in Figure 5, at *x*_g_^LAG^ ≈ 0.2, the PXRD peaks match well those of the BZM-III structure of both systems, with no evidence of BZM-I formation. When comparing the milled samples to the pattern of pure BZM-III, there is an evident shift of some PXRD peaks of the tested sample, indicative of SS formation. A detailed analysis of peak shifts and unit cell variations upon SS formation for both host:guest systems was carried out and is presented in SI. For clarity, however, we only show results for a single peak per system: (i) the (004) peak for the BZM-III[bzm_(1-x)_:ncm_*x*_] system and (ii) the (013) peak for the BZM-III[bzm_(1-x)_:3fbzm_*x*_] system. These peaks were chosen because of being low 2θ peaks, not overlapping with other phases, and having a linear relation between the shift and the guest concentration. The structures of these BZM-III (004) and (013) planes are shown in SI for the interested reader.

Additionally, DSC traces of the milled samples showed single melting events with melting temperatures lower than those of the pure host and guest structures. These results led to the unequivocal conclusion that SS formation had taken place in both systems upon LAG in the presence of IPA together with a switch in the polymorphic crystal structure.

### 3.3 Assessing the Nature and Polymorphic Behavior of the Benzamide Solid Solutions with LAG

**Figure 6:**
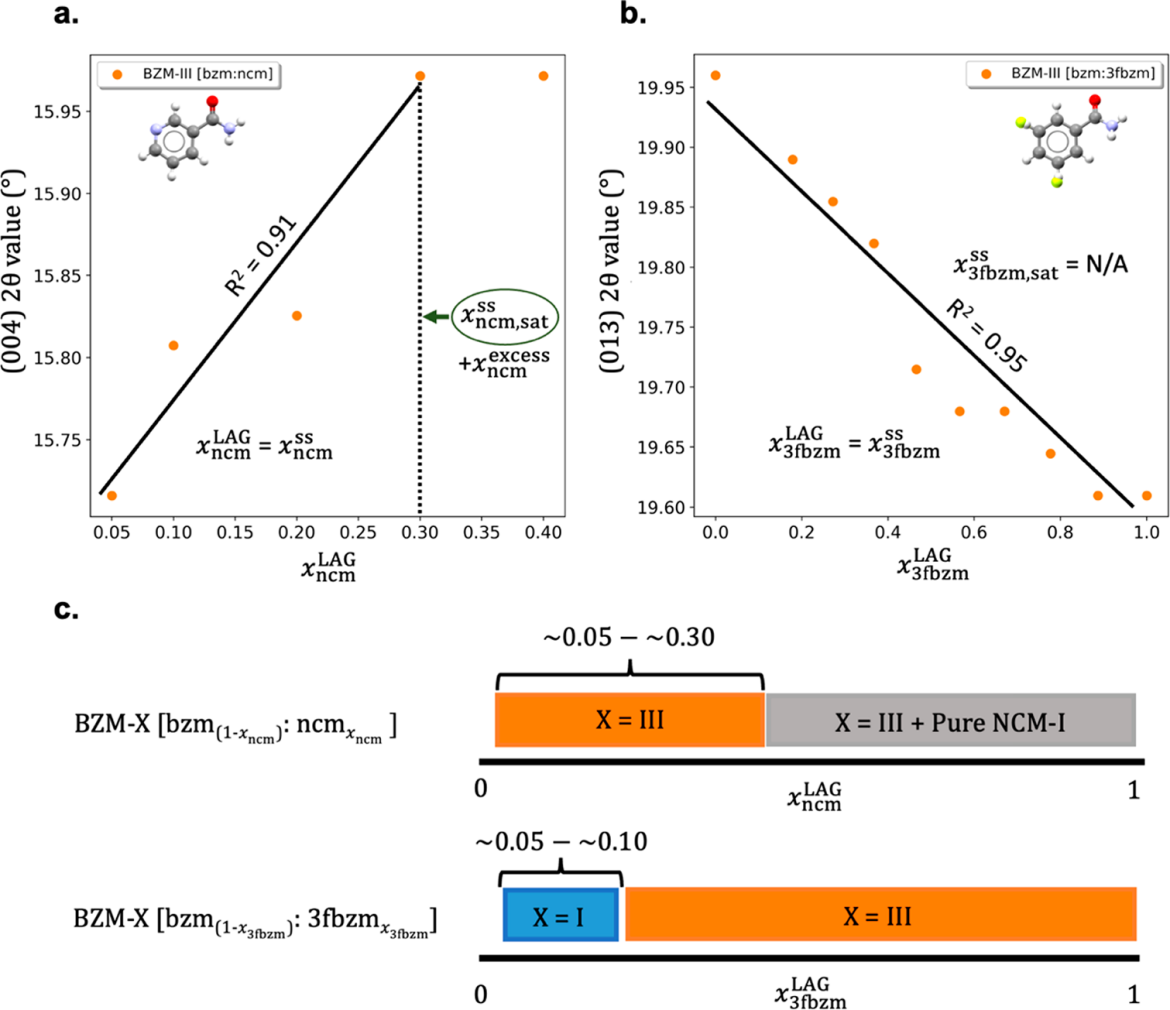
PXRD peak shifts of SS samples produced from LAG with IPA indicate the impact of increasing *x*_g_^LAG^ on the bzm:ncm and bzm:3fbzm systems adopting the BZM-III structure: (a) (004) peak shift as a function of *x*_ncm_^LAG^, (b) (013) peak shift as a function of *x*_3fbzm_^LAG^ (conformational disorder marked by partial occupancy of both meta positions), and (c) switch concentrations and solid-state solubilities for different phases found in the two systems, bzm:ncm - top and bzm:3fbzm - bottom.

After establishing that the two systems form SSs, the subsequent step was to examine the nature and polymorphic behavior of the bzm SSs. This was achieved via analysis of the PXRD patterns obtained at different *x*_g_^LAG^. Diffraction peaks and their positions at different *x*_g_^LAG^ values were compared with those of pure bzm polymorphs and the stable forms of the respective guest compounds. Figure 6 shows the peak shift with changing *x*_g_^LAG^ for peaks corresponding to the (004) plane and the (013) plane in the BZM-III crystal structure for the in bzm:ncm and the bzm:3fbzm SSs, respectively.

The approach used in Figure 6 illustrates another *trick* that can be utilized with PXRD data when studying SSs—analysis of diffraction peak shifts as a function of *x*_g_^LAG^ to determine the solid solubility of a partial SS. Figure 6a demonstrates this clearly for the bzm:ncm system adopting the BZM-III host structure, where a shift in the 2θ position of the (004) plane is observed upon increasing the guest concentration in the LAG experiment until a plateau point is reached. The plateau begins at *x*_ncm_^LAG^ ≈ 0.3, revealing the solid solubility limit of ncm in the host crystal structure BZM-III (*x*_ncm,sat_^SS^). This *x*_ncm,sat_^SS^ value was reconfirmed by a careful analysis of corresponding PXRD patterns and DSC traces of the LAG samples obtained. At *x*_ncm_^LAG^ = 0.3, milled samples analyzed showed evidence of a small amount of NCM-I present. Consequently, the SS achieved in the bzm:ncm system can be classified as a partial SS.

In contrast, a plateau in the peak shift of the (013) plane for the bzm:3fbzm system is never observed (Figure 6b). In this system, the pure BZM-III and 3FBZM-I crystals are isostructural, and thus a continuous shift of peaks across the entire host:guest composition and a formation of a continuous solid solution is observed for the BZM-III host structure. The DSC traces of the milled samples showed single melting events for all concentrations, with a minimum melting temperature observed at *x*_3fbzm_^LAG^ ≈ 0.3 (*T*_m_ ≈ 121 °C). All observations suggest that a continuous SS is formed in the bzm:3fbzm system.

Beside confirming and classifying SSs as partial or continuous, monitoring whether a polymorphic change occurs as a function of *x*_g_^SS^ is also a necessity. For the bzm:ncm system at the tested range of concentrations, the PXRD patterns of all milled products matched that of BZM-III with some shifted peaks. Because BZM-III SSs form at very low concentrations of ncm (*x*_ncm_^LAG^ = 0.05), we were unable to determine the exact guest concentration required for the polymorph stability switch (*x*_ncm,switch_^SS^) with the LAG experiments. This must be in the range 0.00–0.05 (Figure 6c) since pure bzm is most stable as BZM-I. For the bzm:3fbzm system, milled products with *x*_3fbzm_^LAG^ between 0.04 and 0.09 formed SSs matching the crystal structure of BZM-I, while milling with *x*_3fbzm_^LAG^ > 0.18 resulted in SSs with the BZM-III crystal structure. The *x*_3fbzm_^LAG^ for which the polymorphic switch between BZM-I and BZM-III occurs was determined to be between 0.09 and 0.18.

### 3.4 Assessing the Nature and Polymorphic Behavior of Benzamide Solid Solutions with Slurry Experiments

**Figure 7:**
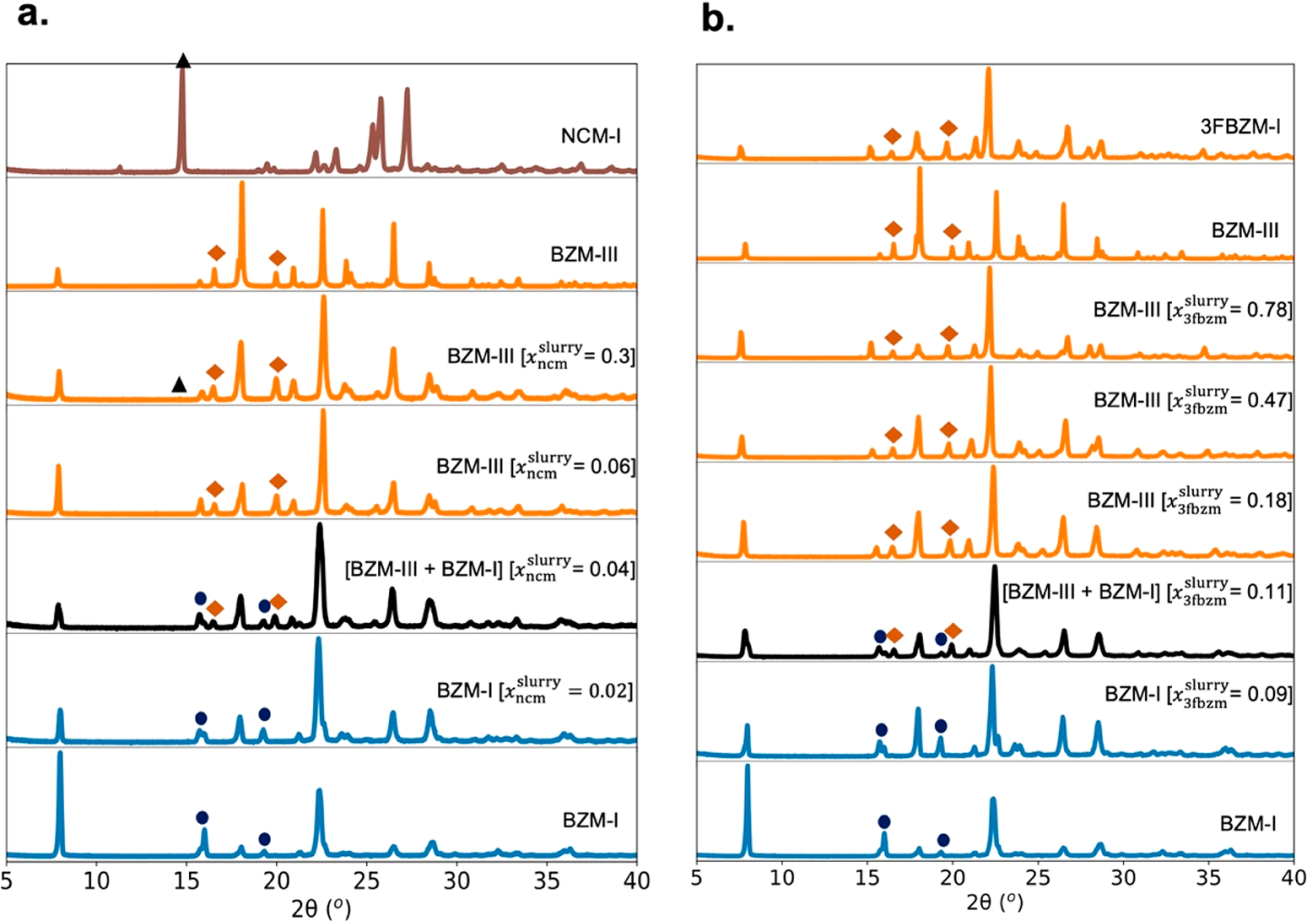
PXRD patterns of slurry crystallites retrieved after isothermal equilibration for 1 week at 25 °C in IPA for (a) the bzm:ncm system and (b) the bzm:3fbzm system. Patterns are colored according to the crystal structure adopted by the major component of the system: BZM-I is shown in blue, BZM-III and isostructural 3FBZM-I are shown in orange, a mixture of BZM-I and BZM-III is shown in black, and NCM-I is shown in brown. Indicative diffraction peaks of the different structures are denoted by symbols: circles for BZM-I, diamonds for BZM-III/3FBZM-I, and triangles for NCM-I. The amounts indicated by *x*_g_^slurry^ refer to the guest mole fraction as a function of the total initial amount of bzm and guest added to the slurries.

Since LAG processes are still not well understood, the assumption that the outcomes of the LAG experiments are due to thermodynamics was validated by enabling solvent-mediated polymorphic transformations (slurry experiments). After sufficient time, slurries of solids in a saturated solution (relative to the existing form) may lead to the nucleation and growth of a more stable (less soluble) polymorph.^34^ Saturated solutions of bzm with excess solids of pure BZM-I and chosen amounts of guest (*x*_g_^slurry^) were prepared in IPA and stirred for 1 week at 25 °C. The resulting solids were filtered and characterized with PXRD (Figure 7), DSC, optical microscopy, and NMR.

The slurry results were in excellent agreement with LAG, confirming that the driving force for the SS formation is thermodynamics; stable SSs were obtained in both bzm:3fbzm and bzm:ncm systems. Consistent with LAG, a partial SS was found for the bzm:ncm system, with excess ncm crystallizing at *x*_ncm_^slurry^ = 0.3, and a continuous SS was found in the bzm:3fbzm system (Figure 7). Further to this, all resulting solids were compared with the corresponding physical mixtures of the pure reactants, providing further unambiguous evidence of SS formation (Supporting Information (SI) 2.4).

In summary, the experiments and multiple characterization analyses of the slurried solids reconfirmed that SSs form for the bzm:ncm and the bzm:3fbzm systems and that their formation and the resulting polymorphic form obtained are driven by thermodynamics. Unlike with LAG, the concentration of the guest in the solution will be different from the final guest concentration in the solids. Thus, further quantification of the amount of guest incorporating into the SS crystal structure is required (*x*_g_^SS^) when the SSs are prepared from solution (i.e., slurry or other crystallization methods).

**Figure 8:**
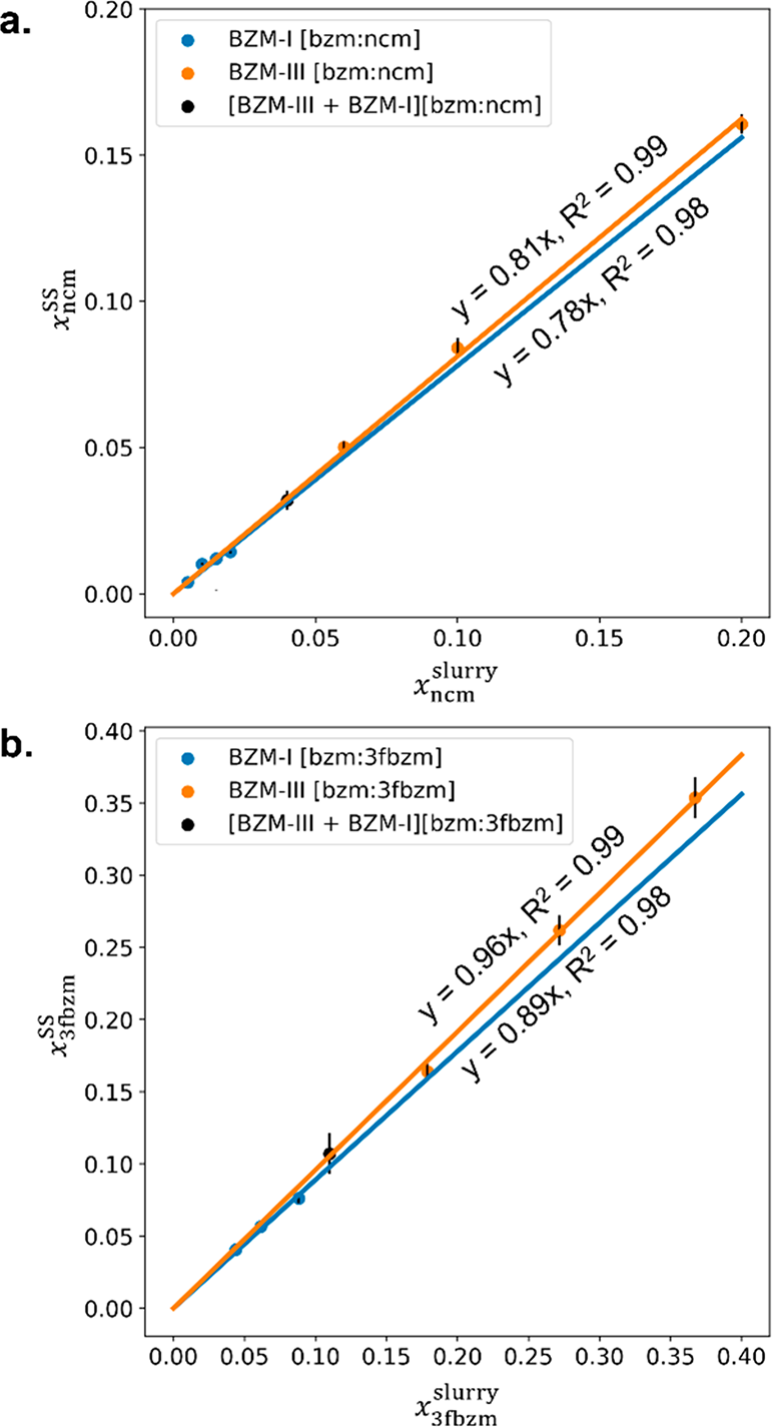
Guest incorporation in the BZM-I and BZM-III SSs obtained under slurry conditions of 25 °C in IPA, (a) for the bzm:ncm system and (b) for the bzm:3fbzm system.

The exact guest uptake into a SS (*x*_g_^SS^) can be analyzed using a quantitative characterization technique such as ^1^H NMR spectroscopy. Figure 8 shows the amount of guest added into the slurry experiments versus the amount of guest incorporated in the resulting SSs (BZM-I and BZM-III) for the ncm and the 3fbzm guests (a and b respectively). The ratio between the amount of guest in the SS and the amount of guest initially in the solution is referred to as “the segregation coefficient” and is calculated from the gradient of the fitted lines in the plots in Figure 8.

The data in Figure 8 show that the 3fbzm guest incorporates with higher efficiency in both BZM-I and BZM-III than ncm. The 3fbzm guest has a segregation coefficient of 0.96 and 0.89 for BZM-III and BZM-I respectively while the ncm guest has a segregation coefficient of 0.81 and 0.78 for BZM-III and BZM-I respectively. The most efficient guest incorporation was found for 3fbzm in the BZM-III structure which is perhaps unsurprising since 3FBZM-I is isostructural with BZM-III (SI 2.2). The guest molecules of ncm, on the other hand, were not taken up by the bzm polymorphs to the same level, perhaps due to the absence of the isostructurality in BZM-III and NCM-I. The difference in uptake efficiency for ncm in BZM-I and BZM-III was also almost negligible (0.03), whereas it was more prominent for the bzm:3fbzm system (0.07).

Finally, as with LAG, the phases obtained as a function of guest incorporation also need to be monitored. If experiments are performed under thermodynamic control (i.e., slurries or prolonged milling), the observation of polymorphic SSs at different concentrations is an indication that a thermodynamic stability “switch” has occurred where a previously metastable polymorph has become stable through the incorporation of guest species. This is, of course, not representative of all crystallization conditions, and metastable polymorphic SSs may also be produced due to kinetics effects during crystallization from solution or the melt.^35−38^ For the case of thermodynamic control, there will be a guest switch concentration (*x*_g,switch_^SS^) below and above which the polymorphic structures change in relative stability. This critical guest switch concentration was estimated based on the corresponding PXRD patterns. In slurries, the guest switch concentration was estimated as the incorporated amount of guest where PXRD peaks corresponding to BZM-III first began to appear, shown in Figure 7 by the black diffraction patterns and in Figure 8 by a black point labeled [BZM-III + BZM-I][bzm: guest]. For the bzm:ncm system from the slurry data, *x*_ncm,switch_^SS^ was estimated to be just 0.032 ± 0.001; this indicates the very small amount of ncm required for a polymorph stability switch. For the bzm:3fbzm system from the slurry data, the *x*_3fbzm,switch_^SS^ value was estimated to be 0.107 ± 0.004. For the second system, a significantly higher amount of guest is required for the switch. In LAG experiments, the guest switch region was approximated to be between the maximum amount of guest tested that resulted in BZM-I and the minimum amount of guest tested that resulted in BZM-III. The switch concentrations obtained from the slurry experiments are in good agreement with those approximated for LAG in section 3.3.

### 3.5 Characterization of Conformational Disorder of the Guest

For the studied systems, both guest compounds can exist in two different conformations (termed A and B in Figure 1) depending on the orientation of the amide functional group relative to the ring substituents (in meta position). We note that, for pure NCM-I,^39^ the guest compound is incorporated in one orientation (ncm_A_) whist for pure 3FBZM-I, 3fbzm is conformationally disordered as 3fbzm_A,0.33_:3fbzm_B,0.66_. There exists, therefore, the possibility that both of these guests may exist in one of those conformations or a combination of both in the SSs.

As the guest component of a SS is typically present in a minor proportion in the host lattice, quantifying its possible conformational disorder is challenging. Several techniques potentially suitable for characterizing guest conformer ratios are shown in Figure 3. One such suitable technique is ssNMR provided that the atomic nucleus of interest is NMR active. For the characterization of the conformational disorder in 3fbzm, ^19^F ssNMR was used, while for the characterization of conformational disorder in ncm, ^13^C ssNMR was used.

**Figure 9:**
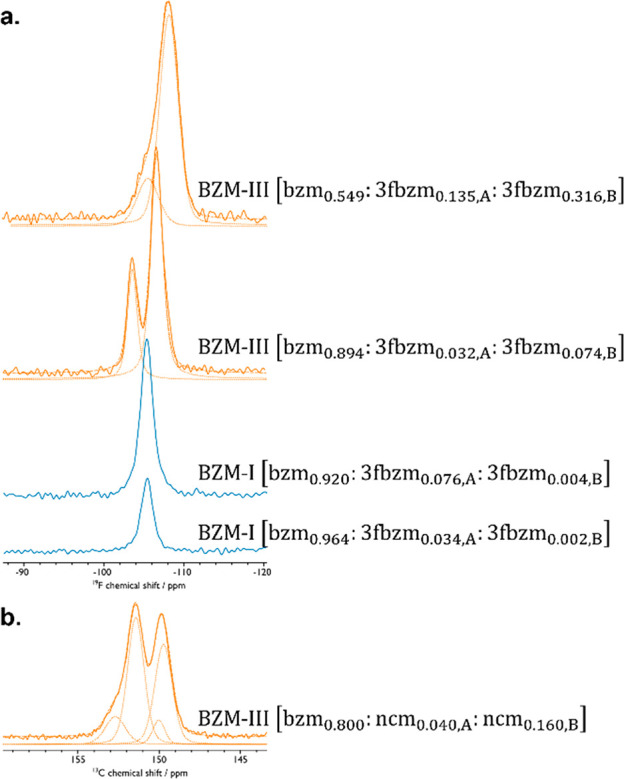
Solid-state magic angle spinning (MAS) NMR characterization to extract guest conformational ratios. Twenty T ^19^F MAS NMR was used for the bzm:3fbzm system, with a MAS frequency of 60 kHz (a) and 9 T {^1^H-}^13^C cross-polarization (CP)MAS NMR was used for the bzm:ncm system, with a MAS frequency of 12 kHz (b). Deconvoluted peak fits are given with dotted lines and their sum with dashed lines. Samples were obtained from 1 week 25 °C slurries, with the bzm:ncm samples then treated by doping with Cu(II) and undergoing neat grinding (b). The ^19^F spectrum for pure 3FBZM-I is provided in SI 2.9.1, while the full ^13^C spectrum for the bzm:ncm system is provided in SI 2.9.2.

For the 3fbzm guest, the ^19^F ssNMR spectra (Figure 9a) indicate that the guest molecule conformer ratio changes dramatically depending on the crystal structure adopted by the SS. When BZM-I-SS is formed, deconvolution of the ^19^F NMR signal in the samples gave an A:B ratio of 3fbzm of approximately 0.95:0.05, where the overall relative conformer fraction of A or B is calculated as *x*_3fbzm,A or B_^SS^/(*x*_3fbzm,A_^SS^ + *x*_3fbzm,B_^SS^). In contrast, when BZM-III-SS is formed, a relative A:B ratio of 0.30:0.70 is observed for 3fbzm independent of its concentration (*x*_3fbzm_^SS^) and consistent with the ratio observed in pure 3FBZM-I. Conducting slurries and analysis of samples at higher and lower temperatures appeared to have a negligible impact on this conformer ratio of the guest observed (SI 2.5). For the ncm guest (Figure 9b), the ^13^C NMR data for BZM-III-SS obtained at *x*_ncm_^slurry^ = 0.2 indicated that the ncm guest is also disordered with a conformational A:B split of 0.2:0.8 of the ncm, with the conformers assigned by comparison to calculated energies from molecular simulations.^23^

Characterization of unit cells and the guest conformations in the SSs was also carried out making use of single crystal X-ray diffraction (SCXRD). Single crystals of good quality were obtained for the SSs from slurries at two (*x*_ncm_^slurry^ = 0.15 and 0.20) and three (*x*_3fbzm_^slurry^ = 0.04, 0.11, and 0.46) different concentrations of ncm and 3fbzm, respectively (Table 2). Relative occupancies of disordered components were used to obtain guest conformer ratios for both guest compounds, which are presented in Table 2. For the bzm:ncm system, all samples obtained adopted the BZM-III structure. Due to the similar X-ray scattering factors of carbon and nitrogen, it was challenging to obtain an accurate crystal structure solution for a disordered three-component system. Neutron diffraction could be useful here, more so if it were to use a deuterated guest. Nevertheless, the best fit to the SCXRD data agreed well with the ssNMR results in indicating that ncm_B_ is the preferred conformer incorporated into BZM-III-SS. For the bzm:3fbzm system, determination of the relative occupancies and the disorder of 3fbzm was more obvious, since the difference in scattering between a hydrogen and a fluorine atom is significant. The SCXRD-obtained conformational populations are in good agreement with the ssNMR data, indicating, again, that the major conformer is 3fbzm_A_ for BZM-I-SS and 3fbzm_B_ for BZM-III-SS. It is important to note that ssNMR gives an overview of the average disorder across the bulk material, whereas SCXRD is performed on selected single crystals which may display a range of disorder values across the system.

**Table 2:** Single Crystal XRD Characterization of BZM-I and -III Pure Together with bzm:ncm and bzm:3fbzm SSs^a^

Guest	Crystal structure	***x***_**g**_^**slurry**^	***x***_**g,A**_^**SS**^	***x***_**g,B**_^**SS**^	Guest A:B ratio^b^	*R* factor	*T*	Unit cell lengths^b^ (*a*, *b*, *c*)	Unit cell angles^c^ (α, β, γ)	Unit cell Volume
–	BZM-I^d^	–	–	–	–	6.3%	123 K	5.549 Å	90.00°	601.74 Å^3^
								5.033 Å	89.22°	
								21.548 Å	90.00°	
–	BZM-III	–	–	–	–	2.7%	100 K	5.042 Å	90.00°	601.95 Å^3^
								5.430 Å	103.80°	
								22.639 Å	90.00°	
–	BZM-III	–	–	–	–	2.1%	150 K	5.048 Å	90.00°	607.54 Å^3^
								5.447 Å	103.86°	
								22.759 Å	90.00°	
ncm	BZM-III	0.15	0.05	0.10	0.33:0.67	4.1%	100 K	5.045 Å	90.00°	598.46 Å^3^
								5.429 Å	103.79°	
								22.499 Å	90.00°	
ncm	BZM-III	0.20	0.06	0.14	0.30:0.70	5.7%	100 K	5.046 Å	90.00°	597.87 Å^3^
								5.432 Å	103.75°	
								22.451 Å	90.00°	
3fbzm	BZM-I	0.04	0.04	0.01	0.80:0.20	4.0%	100 K	5.550 Å	90.00°	602.12 Å^3^
								5.034 Å	90.75°	
								21.550 Å	90.00°	
3fbzm	BZM-III	0.11	0.03	0.07	0.30:0.70	4.5%	150 K	5.042 Å	90.00°	612.72 Å^3^
								5.448 Å	103.33°	
								22.924 Å	90.00°	
3fbzm	BZM-III	0.46	0.10	0.34	0.22:0.78	3.9%	150 K	5.024 Å	90.00°	624.27 Å^3^
								5.444 Å	103.11°	
								23.432 Å	90.00°	

a The guest conformer ratio is resolved in the structure solution. For the SS systems, crystals were obtained after 1 week of slurry experiments at 25°C in IPA.

b A:B ratio in SS = *x*_g,*A*_^SS^/(*x*_g,A_^SS^ + *x*_g,*B*_^SS^).

c We also monitored the changes in unit cell parameters from the pXRD peak shifts and those were found in good agreement to those obtained from SCRXD (see SI).

d Form I crystal structure from the CSD (BZAMID03).

### 3.6 Modeling of Benzamide Solid Solutions

In this section, we show the modeling results for the bzm:ncm and the bzm:3fbzm systems illustrating and validating, against the experimental data, our modeling methodology. For simplicity, we only show the modeling results for the simplest computation, lattice energies of SSs (Grimme-D2) – and the full approach including computation of free energies with improved model energies (TS-MBD), frequency calculations, and consideration of entropy of host:guest mixing and guest disorder. The evolution of results upon adding each of the energetic approximations is fully presented in the Supporting Information.

For a SS to be the most stable form, its free energy must be the lowest overall when compared to the free energy of the other competing systems at a given value of *x*_g_^SS^ (or the general term *x*_g_) including mixtures of multiple phases. The free energies of mixtures of phases can be simply calculated by extrapolating the values *G*_g_^SS^[*x*_g_^SS^] between the phases involved. For example, the free energy of the mixture of pure host and pure guest can be plotted by drawing the line between the free energy of the pure host most stable crystal structure (*x*_g_ = 0) and the free energy of the pure guest most stable crystal structure (*x*_g_ = 1). Energies for mixtures of different SSs can also be calculated by connecting the energies of the two SSs at their respective *x*_g_^SS^ values and then extrapolating to either pure component in excess, depending on *x*_g_^SS^ or *x*_g_. Further details on how these free energies are extrapolated along with a detailed discussion of their significance when evaluating the output of lattice energy calculations can be found in SI 3.4.

**Figure 10:**
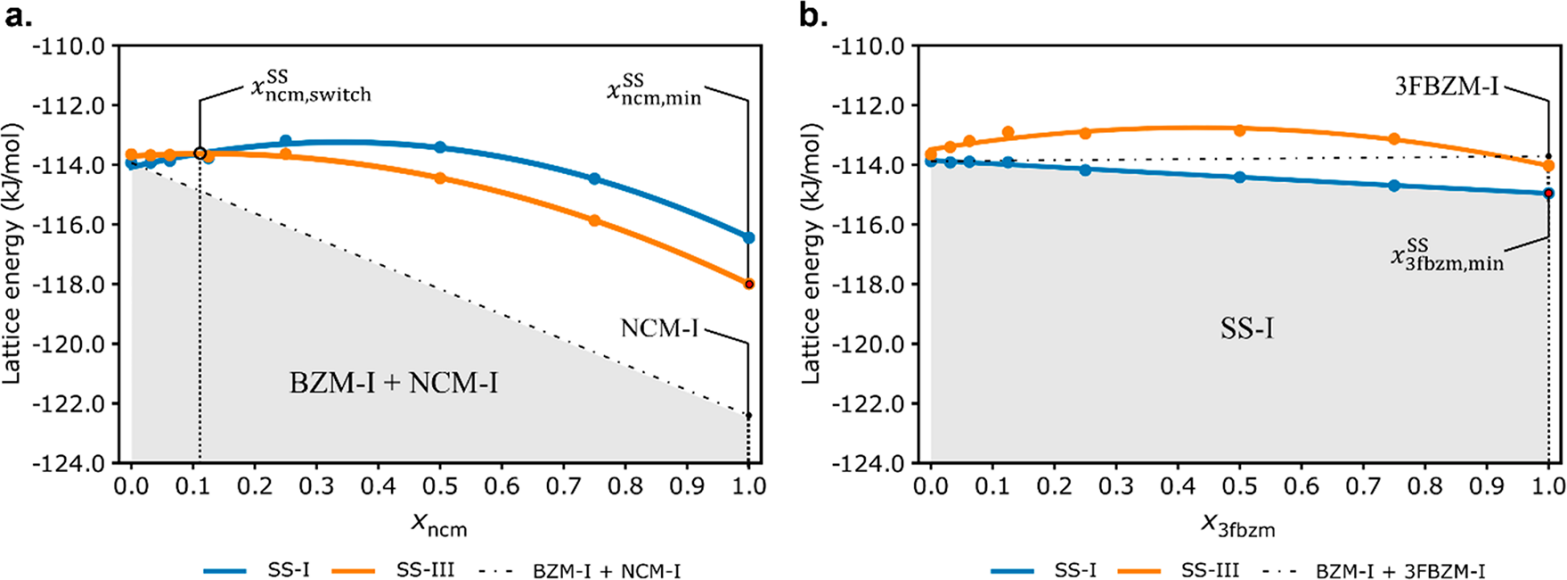
Results from the original Grimme-D2 model for calculating SS energies for bzm:ncm (a) and bzm:3fbzm (b) with no mixing effects considered. Only the conformer producing the lowest energy SS is shown per structure (ncm_B_ in both SSs in bzm:ncm but 3fbzm_A_ in BZM-I-SS and 3fbzm_B_ in BZM-III-SS in bzm:3fbzm). SSs adopting the BZM-I structure are colored blue, while the SSs adopting the BZM-III structure are shown in orange. Black dot-dashed lines are used to represent the energy of statistical physical mixture of the two most stable components (BZM-I + NCM-I, and BZM-I + 3FBZM-I for each SS system, respectively), while a red point denotes the energy of the global minimum SS energy. The lowest energy phases are shaded and labeled BZM-I + NCM-I (a) and SS-I (b) to denote a mixture of pure BZM-I and NCM-I, and a SS with the BZM-I structure, respectively. The same figure with error bars is presented in the SI (3.6.1).

First, we compare the lattice energies of both host:guest systems computed across the entire compositional range (Figure 10), where each plotted point is the mean lattice energy (across all simulation cells considered) at a value of *x*_g_^SS^. For bzm:ncm, although this model shows a polymorph stability switch (SS-I → SS-III), the physical mixture of the pure components (dashed black line) is predicted to be the most stable outcome. For bzm:3fbzm, no polymorph switch is predicted, with SS-I being the most stable form across the entire compositional range. While this methodology was used to predict the polymorph stability switch in the bzm:ncm system before,^21^ when applied to the bzm:3fbzm system and the energetics compared to those of the mixtures of pure forms, the predictions do not validate the experimental outcomes.

**Figure 11:**
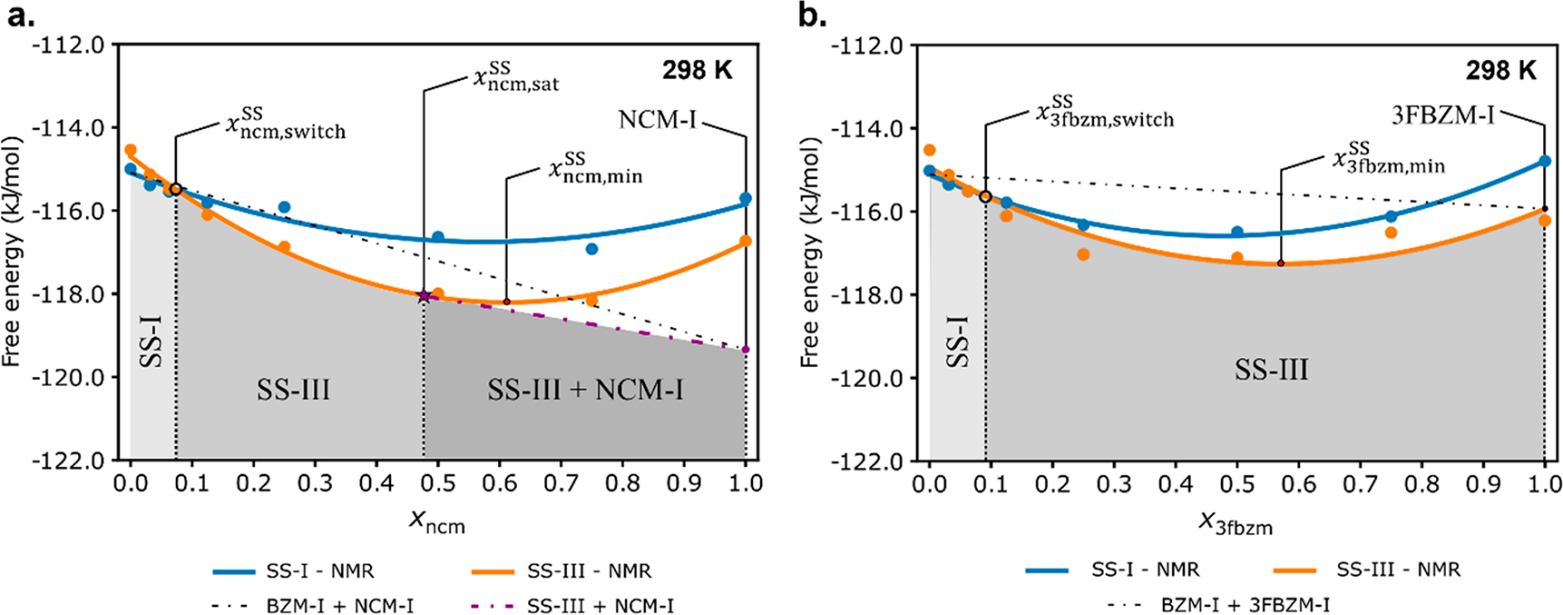
Results from the improved model with ssNMR measured conformational disorder values for bzm:ncm (a) and bzm:3fbzm (b) at 298 K. SSs adopting the BZM-I structure are colored blue, while SSs adopting the BZM-III structure are shown in orange. The black dotted lines and the red points denote the energies of physical mixtures of pure components and the overall minimum SS energy, respectively. The purple dot-dashed line in the bzm:ncm system denotes the energy of the mixture of pure components a SS at the solid saturation point (partial SS) with *x*_ncm,sat._^SS^ marked by a purple star. Phase boundaries corresponding to the lowest energy phase are shaded and labeled, with SS-I and SS-III denoting SSs adopting the BZM-I and BZM-III structures, respectively, while SS-III + NCM-I denote the mixture of BZM-III-SS at *x*_ncm,sat._^SS^ with excess NCM-I. The same figure with error bars is presented in the SI (3.6.2).

Second, we compare the free energies of both host:guest systems computed across the entire compositional range with our full modeling procedure discussed in the previous section (Figure 11). The full modeling approximation renders results in favorable agreement with the experimental findings. For bzm:ncm, the full modeling procedure now predicts SS formation and a polymorph stability switch (SS-I → SS-III) at *x*_ncm,switch_^SS^ = 0.07, which is in good agreement with experiments (*x*_ncm,switch_^SS^ ≈ 0.03). Further, the calculations also predict that the bzm:ncm would only form a partial SS with a predicted *x*_ncm,sat._^SS^ = 0.48. This value is also in good agreement with the experimental *x*_ncm,sat_^SS^ ≈ 0.03. For bzm:3fbzm, the free energy calculations now predict SS formation as well as a change in polymorph stabilities at *x*_3fbzm,switch_^SS^ = 0.09, in excellent agreement with experiment (*x*_3fbzm,switch_^SS^= 0.10). In addition to this, a continuous SS was still predicted, as seen in previous models.

### 3.7 On the Modeling of Guest Disorder

For the modeling of SS disorder in the section above (Figure 11), representative experimental guest disorder values obtained from the ssNMR studies were used for the modeling (see SI 3.3). For a blind conformer disorder prediction, the lowest possible free energy needs to be computed for each SS at each value of *x*_g_^SS^ by varying the conformers A/B ratio from 1:0 to 0:1 in order to find the “optimal conformer disorder ratio”. Incorporation of an enthalpically disfavored conformer offers some gain in entropy due to the additional combinatorial microstates accessible with a third component present in the solid, and can result in a lower overall free energy for the SS.

**Figure 12:**
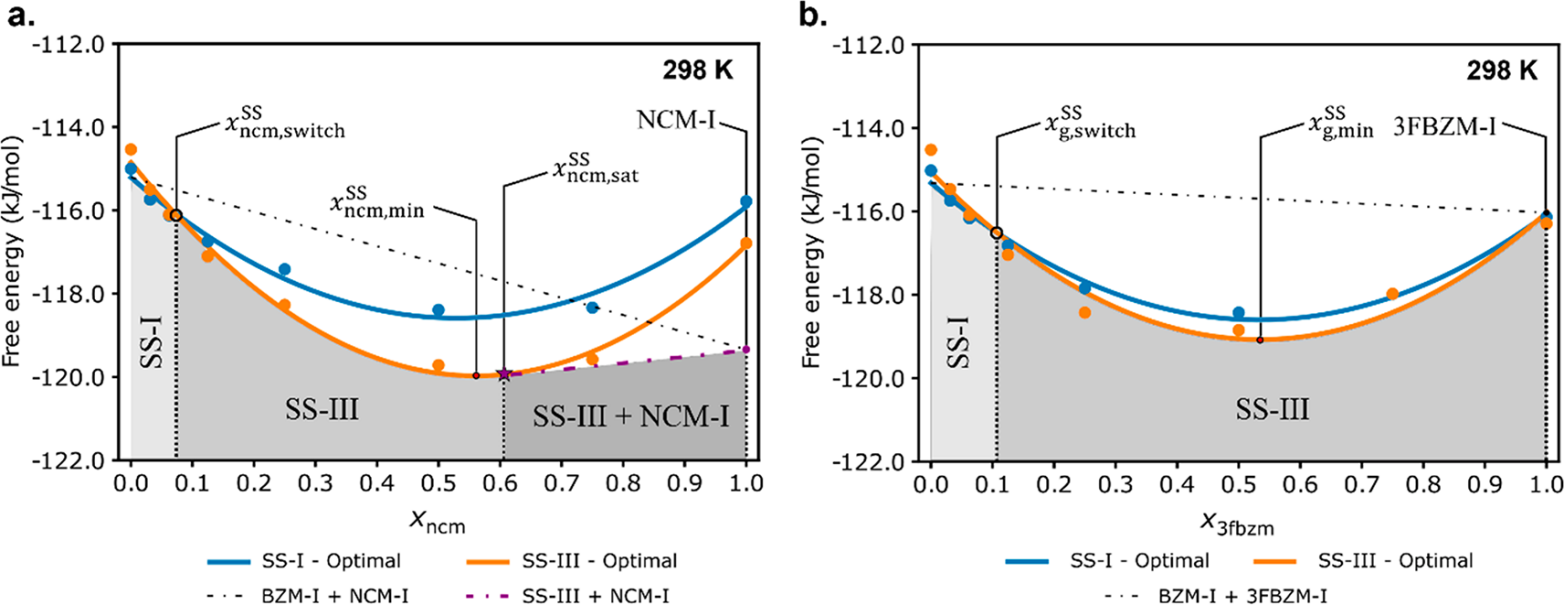
Free energies obtained using the final model with predicted optimal disorder values for bzm:ncm (a) and bzm:3fbzm (b) at 298 K. Points of interest, phase boundaries, and mixture lines are labeled identically to those shown on the ssNMR measured disorder energy diagram. The same figure with error bars is presented in SI (3.6.3).

The free energies obtained from the predicted “optimal conformer disorder ratio” are listed in Figure 12. Overall, the results are again in good agreement with the experimental observations; however, the prediction of the guest switch concentration and the guest saturation values is considerably worse. Further to this, the BZM-I[3fbzm] and BZM-III[3fbzm] are predicted to be almost equal in free energy despite the fact that only BZM-III[3fbzm] is observed experimentally. When the experimentally derived conformer disorder values are used (Figure 11), the theory is in better agreement with the observations.

Modeling of the absolute disorder resulting in the lowest free energies in bzm:ncm demonstrates a clear preference for ncm_B_ in both BZM-I and BZM-III lattices (SI 3.5.1), with BZM-III demonstrating the lowest incorporation of ncm_A_ from the pair of structures (0.3A:0.7B vs 0.1A:0.9B for BZM-I and BZM-III, respectively). Only small changes are observed in the conformer preference with the addition of mixing and vibrational corrections (SI 3.5.2). Disorder preferences vary between BZM-I and BZM-III more dramatically for bzm:3fbzm (SI 3.5.3), with BZM-I preferring 3fbzm_A_ whereas BZM-III prefers a mix of conformers (0.7A:0.3B vs 0.4A:0.6B for BZM-I and BZM-III respectively on average when including mixing and vibrational corrections). Both structures are predicted to incorporate more 3fbzm_B_ with increasing temperature according to the vibrational contributions to the free energy.

## 4 Conclusions

In this study we have presented general methods for communicating, synthesizing, characterizing, and simulating polymorphic SSs. The system of nomenclature has been designed to be robust while also being flexible enough to convey relationships between systems in a concise and clear manner.

Our experimental tricks show a general guide for the synthesis and characterization of SSs, with a deeper dive into the study of polymorphic SSs, with thermodynamic switches caused by the incorporation of guest species. Expansion of these characterization tools to the quantification of guest conformations incorporated in SSs demonstrates the complexity of these systems, where different polymorphic SSs have different conformer preferences, and can drive the incorporation of conformers generally not observed in solution crystallization (SI 3.7.1). These tricks complement existing approaches for synthesizing and characterizing SSs and highlight the information that can be gleaned from different approaches.

**Figure 13:**
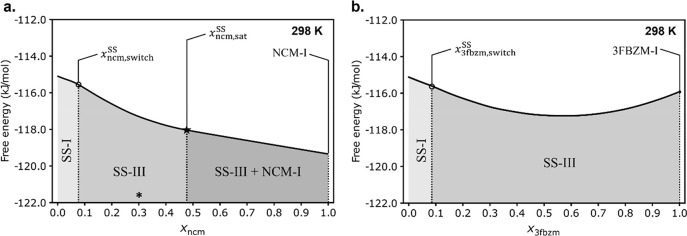
Phase diagram figures displaying only the lowest energy phase across the entire *x*_g_ range at 298 K, using the best performing model compared to experiments (MBD, ssNMR measured disorder, mixing, and temperature/*F*_vib_ effects). For (a) – the bzm:ncm system and (b) – the bzm:3fbzm system. SSs adopting the BZM-I and BZM-III structures are denoted by SS-I and SS-III, respectively. Phase boundaries (*x*_g,switch_^SS^ and *x*_ncm,sat._^SS^) are denoted by dashed lines, shading, and labeling. An asterisk denotes the experimental value of *x*_ncm,sat._^SS^.

We also present a general modeling framework for the calculation of SS energetics, without requiring the evaluation of all combinations of guest and host molecules (although an extended approach will likely only improve the accuracy further). Highly accurate models are required, and each corrective factor has been evaluated in its impact on the energies of SSs. Entropy of mixing is required to stabilize SSs relative to pure components, along with vibrational and conformer mixing terms to extract free energies for polymorphic SSs with accurate guest switch concentrations (*x*_g,switch_^SS^). The latter two of the corrections are crucial for enantiotropic and conformationally disordered systems. Through this type of modeling, we can predict and compose accurate phase diagrams for both continuous and partial polymorphic SSs (Figure 13).

Finally, through the study of two model systems, we have reconfirmed the generality of polymorph stability switches through thermodynamically stable SS formation by inducing a switch in bzm with a second type of guest molecule—3fbzm. Other guest molecules may exist which induce a switch or simply form a SS with the stable BZM-I and have no observable effect on the polymorphic landscape. We are now performing a wide analysis of SS formation with other compounds and have further observed the generality of these switches in several systems, which will be reported shortly.

Polymorphic stability switches have been shown to be an important topic of research, with the possibility of finding efficient routes for crystallizing previously metastable forms of compounds by controlling this phenomenon. Here we have presented a combinatorial experimental and computational workflow for tackling polymorphic SSs. While this article’s focus has been on *thermodynamically* stabilized polymorphic SSs, the possibility of metastable or kinetically stable SSs must be kept in mind. This is particularly important when conducting nonequilibrium-based experiments such as melt or crash cooling crystallization. Slurry experiments are key to avoid the formation of metastable phases and ascertain the thermodynamic stability of SSs.

## 5 Experimental and Computational Methods

### 5.1 Materials

BZM-I (99%) and NCM-I (99%) were obtained from Sigma-Aldrich, and both were confirmed as form I by powder X-ray diffraction (PXRD). 3FBZM-I (99%) was purchased from Acros Organics. Solvents: isopropanol (IPA, reagent grade >99.5%) was obtained from Honeywell; acetone-D6 (deuteration degree −99.8%) was obtained from VWR International Ltd. All materials were used without further purification.

### 5.2 Mechanochemistry (Liquid Assisted Grinding Experiments)

Liquid Assisted Grinding (LAG) experiments were completed using a Retsch MM400 Mixer Mill with screw top 5 mL stainless steel milling jars fitted with a Teflon gasket and 7 mm stainless steel milling balls. Approximately 300 mg of sample and 300 μL of solvent were used. The samples were milled at a frequency of 30 Hz for 60 min. Different ratios of guest:host were tested to examine the impact of guest presence on the host’s polymorphism.

### 5.3 Solvent Mediated Phase Transformations (Slurry Experiments)

Excess solids consisting of different ratios of BZM-I to 3FBZM-I (1.5 g in total) were stirred in 3 g of 2-propanol in sealed 20 mL vials. Temperature control was conducted at the stability of ±0.02 °C using a Polar Bear Plus (Cambridge Reactor Design) with a stirring speed of 250 rpm. Slurries were allowed to equilibrate for 1 week at 25 °C. After 1 week, slurries were filtered using a Büchner flask connected to a vacuum pump and washed with small amounts of isopropanol to aid the separation from saturated solution. The crystallites were characterized immediately via PXRD and after drying at 40 °C in vacuum oven for 48 h, using differential scanning calorimetry (DSC), nuclear magnetic resonance (NMR), and PXRD. Slurry methodology for bzm:ncm system is described elsewhere.^23^

Attempts were also made to examine the impact of temperature on the slurry outcome and configurational conformation of 3fbzm. Slurries containing 12.5 wt % of 3fbzm were tested at 5, 35, 40, 45, and 50 °C. Furthermore, the impact of time of slurring at 3fbzm switch concentration was examined by slurrying for 1 month at 25 °C.

To modify the procedure to produce larger SS crystals for SCXRD, slurries were first heated to 60 °C with all solids dissolved while stirring, then followed by rapid cooling to 25 °C. This temperature was then held for 1 week to allow crystallization and equilibration. Slurries that were stirred, even at a very low speed, produced crystals that were too small to obtain good quality SCXRD data. Consequently, experiments were repeated with stirring turned off once the slurries were cooled to 25 °C. This resulted in suspensions with crystals of sufficient size after 1 week, which were then filtered, dried, and characterized with SCXRD.

### 5.4 Physical Mixtures of BZM-I with NCM-I or 3FBZM-I

For detailed comparison and confirmation of SS formation, physical mixtures of BZM-I and NCM-I or 3FBZM-I were prepared. Various ratios of BZM-I and NCM or 3FBZM-I were weighed out and transferred to vials, followed by subtle mixing with the aid of a spatula and PXRD and DSC characterization procedures.

### 5.5 Characterization Techniques

#### 5.5.1 X-ray Diffraction

##### Powder X-ray Diffraction

PXRD patterns for the different samples were recorded using a Bruker D2 Phaser diffractometer equipped with a LYNXEYE detector in the Bragg–Brentano geometry, using Cu Kα radiation (λ= 1.54 Å). Intensity data were recorded in the 2θ range of 5°–40° at time per step of 0.3s, with a 2θ increment of 0.018° per step.

##### Single Crystal X-ray Diffraction

Single crystal X-ray diffraction (SCXRD) was conducted on BZM-III [bzm_*x*_:ncm_1–*x*_] SSs, BZM-III [bzm_*x*_:fbzm_1–*x*_] SSs, BZM-I [bzm_*x*_:fbzm_1–*x*_] SSs, and pure BZM-III. While the SSs crystals were obtained from slurries, BZM-III crystals were obtained from crash cooling crystallizations, cooled from 50 to 25 °C. BZM-III crystals were retrieved immediately to prevent the conversion of BZM-III to BZM-I. The data for all crystals were collected at 100 and 150 K using an Oxford Cryostream 800 Plus cooling device on a four-circle XtaLAB AFC11 (RINC): Kappa single diffractometer equipped with Hybrid Pixel Array Detector using Cu Kα (λ = 1.54 Å) radiation. Data reduction and face indexing were performed with the CrysAlisPro software (version 171.40_64.69a - Rigaku). OLEX2 (version 1.2)^40^ was used to solve the structures with the SHELXS^41^ program by employing direct methods and to refine the structures with the least-squares procedure using the SHELXL package.^42^ The refinement was based on F^2^ for all reflections, except those with negative intensities. Weighted R factors (*w*R) and all goodness-of-fit (S) values were based on F^2^, whereas conventional R factors were based on the amplitudes, with F set to zero for negative F^2^. Scattering factors were taken from the International Tables for Crystallography.^43^ Crystal data, data collection, and refinement details are summarized in the Supplementary Tables 2.7.1–2.7.3.

Further details on the crystal structure solution methodology for the SSs structures are provided in SI 1.6.5. CCDC deposition numbers are 2253556, 2253557, 2253772, 2253773, 2253774, 2253776, 2253775.

#### 5.5.2 Differential Scanning Calorimetry

DSC characterization was conducted using a TA Instruments DSC 2500 with sealed Tzero aluminum pans. Most samples were heated at a rate of 10 K min^–1^, but for a subset of samples the heating rate was varied to verify its impact on melting events.

#### 5.5.3 NMR

##### ^1^H Solution NMR

The incorporation of guest in BZM-I and BZM-III crystallites was investigated by ^1^H NMR, using a 400 MHz NMR spectrometer (Bruker). Acquisition parameters consisted of 128 scans, 1 s relaxation delay, 65536 of FID points, and 4.09s acquisition time. For each sample obtained from slurry experiments, at least 10 mg of crystals were dissolved in approximately 600 μL of acetone-D_6_ (VWR International Ltd., 99.8% deuteration degree). Data manipulation was performed using the MestReNova software. The host:guest ratios present in slurry crystallites were calculated based on the integrated areas of peaks corresponding to host and guest protons, respectively. The accuracy of these measurements was validated by conducting 5 tests in which known ratios of guest:host were added to NMR tubes and compared with the ratios obtained from the ^1^H spectra. Linearity of the measurements was confirmed, and errors were computed, resulting in an average percentage error of 9.8% and a standard error of 4.3%. The 5 validation tests were also performed with different acquisition parameters (16 scans, 10s relaxation delay, 32768 of FID points, and 2.6s acquisition time), and the obtained ratios were very similar.

##### Solid-State NMR

Magic angle spinning (MAS) NMR spectra were recorded in two regimes. ^19^F ssNMR spectra were recorded on a Bruker 20.0 T (850 MHz ^1^H Larmor frequency) AVANCE NEO spectrometer equipped with a 1.3 mm HXY MAS probe that was used in the ^19^F/^13^C double resonance mode. Experiments were acquired at ambient temperature using a MAS frequency of 60 kHz. ^19^F-pulses of 91 kHz were used, and a Hahn-echo sequence was employed to reduce interference from the probe background and used a free-evolution delay of 10 rotor periods on either side of the π-pulse, giving a total echo duration of 0.333 ms. The ^19^F *T*_1_ is extremely long for these samples (>1000 s), so 8 transients were recorded and coadded, using experimental repetition delays that varied between 300 and 1500 s, depending on the sample. ^13^C ssNMR spectra were recorded on a Bruker 9.4 T (400 MHz ^1^H Larmor frequency) AVANCE III spectrometer equipped with a 4 mm HFX MAS probe that was used in the ^1^H/^19^F/^13^C triple resonance mode. Experiments were acquired at ambient temperature using a MAS frequency of 12 kHz. ^1^H- and ^13^C-pulses of 100 and 50 kHz were used, respectively, and spectra were recorded after {^1^H-}^13^C cross-polarization (CP) and a Hahn-echo sequence that used a free-evolution delay of 1 rotor period either side of the π-pulse, giving a total echo duration of 0.167 ms. For CP, a 70–100% ramp was used for ^1^H (∼73 kHz ν_rf_ at 100%) to match 50 kHz ^13^C spin-locking. The ^1^H *T*_1_ is extremely long for these samples (>1000 s), so 1.6 mol % of Cu (from anhydrous CuCl_2_) was neat ground using a Retsch MM400 Mixer Mill at 30 Hz for 30 min along with crystals from the bzm:ncm system obtained from a slurry. This resulted in the ^1^H *T*_1_ being reduced to ∼1 s. 36 200 transients were coadded, with a repetition delay of 1.3*T*_1_. PXRD analysis showed that this subsequent neat grinding with CuCl_2_ did not affect the crystal structure.

### 5.6 Computational Methodology

#### 5.6.1 Optimization Procedure

The approach for calculating *E*_latt_ for SSs was based on previous work on bzm:ncm by this group.^23^ All structures were optimized with DFT-d, using the VASP software (version 5.4.4),^44−47^ employing the PBE functional^48^ along with PAW pseudopotentials.^49,50^ Three separate methods were employed to calculate the van der Waals dispersion force contributions to the electronic energies: Grimme’s D2 method,^48^ the Tkatchenko–Scheffler (TS) method,^51^ and the many-body dispersion (MBD) energy method^52,53^ (implemented in k-space).^54^ Each optimized cell and atom positions were fed through to the following optimization procedure in the following workflow: Grimme → TS → MBD.

#### 5.6.2 SS Lattice Energy Calculations

To calculate *E*_latt_, a reference to the molecule in the gas phase is required. This value was obtained by optimizing a single molecule of bzm in a fixed 20 Å × 20 Å × 20 Å supercell, with matching energetic and dispersion constraints as the solid phase. This was also performed for both conformers of 3fbzm and ncm, with 3fbzm_B_ and ncm_A_ (Figure 1) chosen due to their lower energies from each conformer pair when optimized in the gas phase. *E*_latt,g_^SS^ for a SS at a defined *x*_g_^SS^ can be computed according to the following equation:
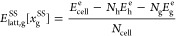
where *N*_cell_, *N*_h_, and *N*_g_ denote the total number of molecules present in the SS supercell, the number of host (bzm) molecules present, and the number of guest (ncm or 3fbzm) molecules present in the supercell, respectively, and *E*_cell_^e^, *E*_h_^e^, and *E*_g_^e^ denote the electronic energy of the SS supercell along with host and guest in the gas phase, respectively. *x*_g_^SS^ denotes the mole fraction of the guest that has incorporated into the SS, as the values of *N* are dependent on the amount of guest and host molecules present in the unit cell. Replacing the guest terms with corresponding ncm and 3fbzm terms yields *E*_latt,g_^SS^[*x*_g_^SS^] for the bzm:ncm and bzm:3fbzm SS systems, respectively. To plot the *E*_latt,g_^SS^[*x*_g_^SS^] values where more than one simulation cell permutation is available (whether through altering the unit cell axes or selecting a different molecule set for replacement), the mean value for the energies is taken. Standard errors (standard deviation divided by the square root of the sample size) can also be computed from the data and are presented in SI 3.6 for the lattice energy and free energy plots.

#### 5.6.3 Vibrational Mode Calculations

An approach used previously within our group for tolfenamic acid^55^ was extended to multicomponent solids to investigate the effect of temperature on *E*_latt,g_^SS^ originally computed at 0 K. Vibrational modes were calculated using VASP, via the finite-difference method and converted to energy values with an in-house Python script. Sufficiently sized supercells of the TS optimized structures were used to perform these calculations, as recommended in literature.^29,56^ Temperature corrections were then added as a relative value compared with the BZM-I [bzm_*x*_:guest_1–*x*, A_] cell at each guest concentration. Adding this parameter as a relative term ensures that vibrational mode contributions are only computed from solid phases, and not for the gas phase reference molecule (the previous approaches utilized a Δ*E*_latt_ term exclusively for comparing polymorphs, and thus did not require a gas phase reference state).

#### 5.6.4 Physical Mixture Energy Calculations

The energies for the physical mixtures of BZM-I and NCM-I or 3FBZM-I were computed assuming linearity between the *E*_latt_ of the experimentally observed stable pure components. Pure *E*_latt_ values were calculated using the same methodology and parameters as those for the SS cells (including entropy and vibrational contributions where appropriate). This can be extended to mixtures of SSs by adding data points at their respective *x*_g_^SS^ values, linking each SS point together with increasing *x*_g_, and then extrapolating said points for the SSs at the maximum and minimum points on the *x*_g_ scale to the energy for pure guest and pure host energies, respectively.

Across this paper, only the conformers which result in the lowest *E*_latt,g_^SS^ values for BZM-I and BZM-III are shown for each structure. Full discussion of the computational methodology is provided in SI 1.3, while additional computational results are provided in SI 3.

## Supporting Information Available

Materials and methods for synthesis and characterization, further details on computational studies, supplementary experimental results and characterization, supplementary computational results, supplementary Figures 2.3.1 to 3.7.2 and Tables 1.2.1 to 2.7.3 (PDF)
