# Supplementary material for: Polymorphic Solid Solutions in Molecular Crystals: Tips, Tricks, and Switches
Source: J Am Chem Soc. 2023 Sep 6;145(37):20562–77. doi: 10.1021/jacs.3c07105 (PMC10515635; doi:10.1021/jacs.3c07105)
Supplement: Supplementary file 1 — ja3c07105_si_001.pdf [file ja3c07105_si_001.pdf]

Polymorphic Solid Solutions in Molecular Crystals: Tips, Tricks, and Switches

Adam Hill,[a, b] Weronika Kras,[b] Fragkoulis Theodosiou,[a, b] Monika Wanat,[b, c] Daniel Lee,[b] Aurora J. Cruz-Cabeza*[a, b]

[a]Department of Chemistry, University of Durham, Lower Mount Joy, South Rd, Durham, DH1 3LE, UK

[b]Department of Chemical Engineering, The University of Manchester, Oxford Road, Manchester, M13 0PL, UK

[c]Faculty of Chemistry, University of Warsaw, Pasteura 1, 02-093 Warsaw, Poland

*Corresponding Author: aurora.j.cruz-cabeza@durham.ac.uk

Table of Contents

Supplementary Methods 3

1.1 Materials 3

1.2 Liquid Assisted Grinding (LAG) 3

1.3 Molecular Simulations 5

1.4 Slurry Experiments 15

1.5 Crash Cooling Experiments 17

1.6 Characterisation Techniques 17

1.6.1 Powder X-ray Diffraction (PXRD) measurements 17

1.6.2 Differential Scanning Calorimetry (DSC) measurements 17

1.6.3 Nuclear Magnetic Resonance (NMR) measurements 17

1.6.4 Optical Microscopy 18

1.6.5 Single Crystal X-ray Diffraction (SCXRD) Measurements 18

1.6.6 Solid-State NMR 18

Supplementary Results 19

2.1 Identification of polymorphs 19

2.2 Crystal packing comparison 19

2.3 LAG experiments 19

2.4 Physical Mixtures 22

2.5 Slurry Experiments 24

2.6 Unit Cell Parameters 28

2.7 SCXRD 30

2.8 Optical microscopy images 33

2.9 Supplementary ssNMR spectra 34

Supplementary Computational Results 35

3.1 Impact of Dispersion Corrections 35
3.2 Impact of Mixing Entropy 36
3.3 Impact of Disorder 37
3.4 Free Energy vs. Physical Mixtures – A Detailed Discussion 39
3.5 Disorder Ratio Calculations 43
3.6 SS Energies with Error Bars 46
3.7 Studies of Molecular ncm and 3fbzm 48
Supplementary References 49

Supplementary Methods

1.1 Materials

BZM-I and NCM-I were purchased from Sigma Aldrich (≥ 99 %, United Kingdom) and 3FBZM-I was purchased from Acros Organics (99%), respectively. Both were used without further purification. Deuterated acetone (acetone D6, 99.8%) was purchased from VWR International Ltd. and used without any further purification for NMR analysis. Isopropanol (IPA) was supplied by Honeywell Research Chemicals (> 99%).

1.2 Liquid Assisted Grinding (LAG)

LAG experiments in IPA were conducted using the Retsch Mixer Mill MM 400. Approximately, a total mass of 0.3 g of sample, 300.0 µL of isopropanol and 7 mm diameter stainless steel ball were loaded to a 5 ml steel jar. After addition of 300.0 µL of isopropanol to each jar, grinding for 1 h at 30Hz was conducted. The same procedure was followed for bzm:ncm and is described elsewhere.[1] Supplementary Table 1.2.1 and 1.2.2 details the compositions used in the LAG experiments.

Supplementary Table 1.2.1 3fbzm and bzm quantities used in LAG in wt%

Solvent	Wt.%[a] 3fbzm	Mass 3fbzm (g)	Wt.%[a] bzm	Mass bzm (g)
IPA	5	0.015	95	0.285
	10	0.030	90	0.270
	20	0.060	80	0.240
	30	0.090	70	0.210
	40	0.120	60	0.180
	50	0.150	50	0.150
	60	0.180	40	0.120
	70	0.210	30	0.090
	80	0.240	20	0.060
	90	0.270	10	0.030
	100	0.300	0	0

[a] wt.% with respect to the total solid load used

Supplementary Table 1.2.2 3fbzm and bzm mol fractions used in LAG.

Solvent	Wt.%[a] 3fbzm	x_{3fbzm}^{LAG}	Wt.%[a] bzm	x_{bzm}^{LAG}
IPA	5	0.04	95	0.96
	10	0.09	90	0.91
	20	0.18	80	0.82
	30	0.27	70	0.73
	40	0.37	60	0.63
	50	0.47	50	0.53
	60	0.57	40	0.43
	70	0.67	30	0.33
	80	0.78	20	0.22
	90	0.89	10	0.11
	100	1	0	0.00

[a] wt.% with respect to the total solid load used

1.3 Molecular Simulations

Crystal structure models

The crystal structures of BZM-I and III were retrieved from the Cambridge Structural Database[2] (CSD refcodes BZAMID05[3] and BZAMID08[4] respectively). Basic crystallographic information for these two forms is provided in Supplementary Table 1.3.1.

Crystallographic information of all reported structures of bzm, nicotinamide (ncm) and 3fbzm are reported in Supplementary Tables 1.3.2, 1.3.3 and 1.3.4, respectively.

Supplementary Table 1.3.1. Crystallographic information for bzm forms I and III.

	**Form I**	**Form III**
REFCODE	BZAMID05	BZAMID08
T (K)	173	283-303
Space Group	$P2_1/c$	$P2_1/c$
Z'	1	1
a, b, c (Å)	5.57, 5.04, 21.70	5.06, 5.51, 22.96
α, β, γ(°)	90.0, 90.4, 90.0	90.0, 101.3, 90.0

Supplementary Table 1.3.2 Crystallographic information for crystal structures of bzm reported in the CSD.

Form	CSD Refcode	Space Group	Unit Cell parameters a, b, c (Å) α, β, γ (°)	Z	Z’	R-factor(%)	Temperature (K)	Notes
Form I*	BZAMID	$P2_1/c$	5.59, 5.01, 21.93 90.00, 90.75, 90.00	4	1	8.70	283-303	N/A
	BZAMID01	$P2_1/c$	5.61, 5.05, 22.05 90.00, 90.66, 90.00	4	1	6.80	283-303	N/A
	BZAMID02	$P2_1/c$	5.53, 5.03, 21.34 90.00, 88.73, 90.00	4	1	4.40	15	N/A
	BZAMID03	$P2_1/c$	5.55, 5.03, 21.55 90.00, 89.22, 90.00	4	1	6.30	123	N/A
	BZAMID04	$P2_1/c$	5.55, 5.03, 21.55 90.00, 89.22, 90.00	4	1	4.70	123	N/A
	BZAMID05	$P2_1/c$	5.57, 5.04, 21.70 90.00, 90.39, 90.00	4	1	3.76	173	N/A
	BZAMID07	$P2_1/c$	5.61, 5.04, 22.12 90.00, 90.64, 90.00	4	1	5.33	283-303	Error: N and O positions switched in structure solution
	BZAMID09	$P2_1/c$	5.61, 5.04, 22.12 90.00, 90.64, 90.00	4	1	5.33	283-303	Missing 3D coordinates
	BZAMID14	$P2_1/c$	5.57, 5.04, 21.70 90.00, 90.50, 90.00	4	1	5.00	173	N/A
	BZAMID15	$P2_1/c$	5.57, 5.03, 21.75 90.00, 90.18, 90.00	4	1	3.67	200	N/A
Form II	BZAMID06	*Pba2*	17.43, 14.19, 4.98 90.00, 90.00, 90.00	8	2	8.60	283-303	Z and Z’ incorrectly listed
	BZAMID13	*Fdd2*	28.39, 34.86, 5.14 90.00, 90.00, 90.00	32	2	14.89	14.89	N/A
	BZAMID13[5] (revised)	$P2_1/c$	5.17, 16.92, 14.59 90.00, 103.94, 90.00	8	2	0.86/9.62[a]	N/A[b]	CSP predicted
Form III	BZAMID08	$P2_1/c$	5.06, 5.51, 22.96 90.00, 101.29, 90.00	4	1	5.32	283-303	N/A
	BZAMID10	$P2_1/c$	5.06, 5.51, 22.96 90.00, 101.29, 90.00	4	1	5.32	283-303	Missing 3D coordinates

	BZAMID11	*P2₁/c*	5.05, 5.42, 21.95<br>90, 90.92, 90.00	4	1	4.20	90	N/A
	BZAMID12	*P2₁/n*	5.06, 5.46, 22.83<br>90.00, 103.85, 90	4	1	3.98	173	N/A

[a] values are R_{exp}(%) including background, and with a background subtraction, respectively.
[b] Crystal structure prediction result, temperature factors for each molecule in the asymmetric unit (2) restrained to 5 Å^2
* Stable polymorph

Supplementary Table 1.3.3 Crystallographic information for crystal structures of ncm reported in the CSD.

Form	CSD Refcode	Space Group	Unit Cell parameters a, b, c (Å) α, β, γ (°)	Z	Z'	R-factor(%)	Temperature (K)	Notes
Form I (α)*A	NICOAM	*P2₁/a*	9.44, 15.65, 3.97 90.00, 99.13, 90.00	4	1	19	283-303	N/A
	NICOAM01	*P2₁/c*	3.88,15.6, 9.38 90.00, 98.45, 90.00	4	1	5.4	150	N/A
	NICOAM02	*P2₁/c*	3.98, 15.63, 9.42 90.00, 99.03, 90.00	4	1	5.7	283-303	N/A
	NICOAM03	*P2₁/c*	3.98, 15.63, 9.42 90.00, 99.03, 90.00	4	1	6	283-303	N/A
	NICOAM05	*P2₁/c*	3.98, 15.64, 9.43 90.00, 99.02, 90.00	4	1	0.56	283-303	N/A
	NICOAM06	*P2₁/c*	3.86, 15.63, 9.37 90.00, 98.18, 90.00	4	1	1.47	90	N/A
	NICOAM13	*P2₁/c*	3.88, 15.65, 9.38 90.00, 98.39, 90.00	4	1	5.38	100	N/A
Form II (β)A	NICOAM04	*P2/n*	15.06, 10.70, 15.21 90.00, 102.19, 90.00	16	4	4.99	173	N/A
	NICOAM14	*P2/n*	14.99, 10.68, 15.19 90.00, 101.96, 90.00	16	4	3.65	100	N/A
Form III (γ)†‡	NICOAM15	*P2₁/c*	15.37, 7.38, 21.12 90.00, 104.65, 90.00	16	4	7.49	100	N/A
Form IV (δ)†‡	NICOAM16	*P2₁/c*	7.35, 20.74, 7.41 90.00, 91.04, 90.00	8	2	6.09	100	N/A
Form V (ε)†B	NICOAM07	*P2₁*	3.81, 14.39, 5.12 90.00, 94.26, 90.00	2	1	2.51	100	N/A
	NICOAM10	*P2₁*	3.81, 14.39, 5.12 90.00, 94.26, 90.00	2	1	2.51	100	Missing 3D coordinates in CSD (Redetermination of 07)
Form VI (ζ)†A	NICOAM18	*P*1̄	7.56, 7.94, 10.80 108.10, 102.60, 98.29	4	2	5.45	100	N/A

Form VII (η)[†A]	NICOAM08	$P\bar{1}$	3.75, 12.32, 13.06 71.50, 85.68, 85.20	4	2	6.12	100	N/A
	NICOAM11	$P\bar{1}$	3.75, 12.32, 13.06 71.50, 85.68, 85.20	4	2	6.12	100	Missing 3D coordinates in CSD (Redetermination of 08)
Form VIII (θ)[†‡]	NICOAM09	$P2_1$	10.70, 35.19, 15.83 90.00, 102.68, 90.00	40	20	6.04	100	N/A
	NICOAM12	$P2_1$	10.70, 35.19, 15.83 90.00, 102.68, 90.00	40	20	6.04	100	Missing 3D coordinates in CSD (Redetermination of 09)
Form IX (ι)[†A]	NICOAM17	$P2_1/c$	9.90, 5.87, 10.28 90.00, 100.00, 90.00	4	1	6.4	100	N/A

* Stable polymorph
[†] Polymorph isolated from melt crystallisation
[A] Composed only of conformer A
[‡] Composed of a mixture of conformers A and B
[B] Composed only of conformer B

Supplementary Table 1.3.4 Crystallographic information for crystal structures of 3fbzm reported in the CSD.

Form	CSD Refcode	Space Group	Unit Cell parameters a, b, c (Å) α, β, γ (°)	Z	Z'	R-factor(%)	Temperature (K)	Notes
Form I*	BENAFM10	$P2_1/a$	23.78, 5.56, 5.04 90.00, 100.30, 90.00	4	1	13.20	283-303	Major occupancy only entered in file (conformer ratio is 0.67 : 0.33)
	BENAFM11	$P2_1/n$	5.03, 5.50, 23.05 90.00, 92.48, 90.00	4	1	3.51	173	F is disordered over two sites (conformer ratio is 0.669 : 0.331)

* Stable polymorph

Optimisation Procedure

All cells were optimised with DFT-d, using the VASP software (version 5.4.4),[6–9] employing the PBE functional[10] along with PAW pseudopotentials.[11,12] Three separate methods were employed to calculate the van der Waals dispersion force contributions to the electronic energies: Grimme's D2 method,[10] the Tkatchenko-Scheffler (TS) method,[13] and the many-body dispersion (MBD) energy method[14,15] (implemented in *k*-space).[16] A 520 eV cut-off was enforced for the planewave kinetic energies in all cases, and each structural optimisation was stopped when the force on each atom was below 0.003 eV $Å^{-1}$ (this value was reduced for the vibrational mode calculations, discussed later). The energy convergence criterion for energy difference between calculation steps was 10^{-7} eV for standard simulations. To sample the Brillouin zone, the Monkhurst-Pack approximation was applied, with *k*-point spacing of at least 0.04 $Å^{-1}$.[17] The energy optimisation process can be summarised in four steps:

I. optimisation with Grimme-D2 dispersion corrections, allowing the unit cell parameters to vary,
II. optimisation with Grimme-D2 dispersion corrections, with the unit cell parameters fixed,
III. optimisation with TS dispersion corrections, allowing the unit cell parameters to vary,
IV. optimisation with TS dispersion corrections, with the unit cell parameters fixed

with the optimised structure from each step feeding into the following step. After the fourth stage, the optimised structure was used as an input for a single point MBD calculation and a vibrational phonon mode calculation.

The lattice energy simulation setups for the solid solution (SS) and pure systems are summarised in Supplementary Table 1.3.5 (the same parameters were used for both bzm:ncm and bzm:3fbzm SS systems).

Supplementary Table 1.3.5 Cells and parameters used for SS and pure system lattice energy calculations (E_{latt})

Form	x_g	Supercell Construction	Input Cell Parameters[a]	Molecules in Cell	bzm Molecules in Cell	Guest Molecules in Cell	k-points
I (BZAMID05)	0[b]	1x1x1	5.6 x 5.0 x 21.7	4	4	0	4x4x1
	0.03125	2x4x1	11.1 x 20.1 x 21.7	32	31	1	2x1x1
	0.03125	4x2x1	22.3 x 10.1 x 21.7	32	31	1	1x2x1
	0.0625	1x4x1	5.6 x 20.1 x 21.7	16	15	1	4x1x1
	0.0625	2x2x1	11.1 x 10.1 x 21.7	16	15	1	2x2x1
	0.0625	4x1x1	22.3 x 5.0 x 21.7	16	15	1	1x4x1
	0.125	1x2x1	5.6 x 10.1 x 21.7	8	7	1	4x2x1
	0.125	2x1x1	11.1 x 5.0 x 21.7	8	7	1	2x4x1
	0.25	1x1x1	5.6 x 5.0 x 21.7	4	3	1	4x4x1
	0.5	1x1x1	5.6 x 5.0 x 21.7	4	2	2[c]	4x4x1
	0.75	1x1x1	5.6 x 5.0 x 21.7	4	1	3[c]	4x4x1
	1	1x1x1	5.6 x 5.0 x 21.7	4	0	4[c]	4x4x1
III (BZAMID08)	0[c]	1x1x1	5.1 x 5.5 x 23.0	4	4	0	4x4x1
	0.03125	2x4x1	10.1 x 22.1 x 23.0	32	31	1	2x1x1
	0.03125	4x2x1	20.2 x 11.0 x 23.0	32	31	1	1x2x1
	0.0625	1x4x1	5.1 x 22.1 x 23.0	16	15	1	4x1x1
	0.0625	2x2x1	10.1 x 11.0 x 23.0	16	15	1	2x2x1
	0.0625	4x1x1	20.2 x 5.5 x 23.0	16	15	1	1x4x1
	0.125	1x2x1	5.1 x 11.0 x 23.0	8	7	1	4x2x1
	0.125	2x1x1	10.1 x 5.5 x 23.0	8	7	1	2x4x1
	0.25	1x1x1	5.1 x 5.5 x 23.0	4	3	1	4x4x1
	0.5	1x1x1	5.1 x 5.5 x 23.0	4	2	2[c]	4x4x1
	0.75	1x1x1	5.1 x 5.5 x 23.0	4	1	3[c]	4x4x1
	1	1x1x1	5.1 x 5.5 x 23.0	4	0	4[c]	4x4x1
I (NICOAM05)	0[b]	1x1x1	4.0 x 15.6 x 9.4	N/A	N/A	N/A	4x2x3
I (BENAFM11)	0[b]	1x1x1	5.0 x 5.5 x 23.1	N/A	N/A	N/A	4x4x1

a – cells as input into the first Grimme-D2 optimisation stage

b – pure systems calculated for comparisons with physical mixture E_{latt}

c – E_{latt} calculated for all permutations of guest molecules where more than one guest molecule was present.

Solid solution lattice energy calculations

Starting from CSD refcodes BZAMID05 and BZAMID08 for BZM-I and III respectively, supercells were created to emulate SS structures with 3fbzm and ncm across a range of stoichiometries using a purpose written Python code. As both BZM-I and BZM-III both have four molecules in their unit cells, the values of $x_{\mathrm{g}}^{\mathrm{SS}}$ studied were: 0.03125, 0.0625, 0.125, 0.25, 0.5, 0.75 and 1. Lower mole fractions were ignored due to the large supercell size requirements. At $x_{\mathrm{g}}^{\mathrm{SS}}$ of 0.25 and below, one molecule of bzm was replaced with 3fbzm or ncm per supercell, whereas two, three and four molecules were replaced for $x_{\mathrm{g}}^{\mathrm{SS}}$ = 0.5, 0.75 and 1, respectively. As the replacement of multiple molecules raises questions about the interaction of neighbouring guest molecules, all permutations of molecule replacement were computed for $x_{\mathrm{g}}^{\mathrm{SS}}$ 0.5, (six permutations, with only 3 symmetry-unique permutations) along with 0.25 and 0.75 (four permutations each) to calculate the mean, minimum and maximum $E_{\mathrm{latt,g}}^{\mathrm{SS}}$ for each guest concentration. The effect of supercell axis permutations on the overall energy were also investigated (e.g. 1x2x1 vs 2x1x1) for the *a* and *b* unit cell dimensions only, given that these measure ~5 Å in both BZM-I and BZM-III vs. ~20 Å for the *c* dimension. As the phenyl ring in either guest molecule can rotate around a bond to produce two inequivalent conformers, two versions of each supercell were created, one where the bzm molecules were replaced with $3fbzm_A$ or ncm_A and one with the molecules replaced with $3fbzm_B$ or ncm_B.

To calculate the difference in energy caused by intermolecular interactions and conformational rotation in the crystal structure, a reference point of the molecule in the gas phase is required for $E_{\mathrm{latt,g}}^{\mathrm{SS}}$. This value was obtained by optimising the geometry of a single molecule of bzm in a fixed 20 x 20 x 20 Å supercell with matching energetic and dispersion constraints to the solid phase (previously discussed). Both conformers of 3fbzm and ncm were optimised, with $3fbzm_B$ chosen as a reference state for 3fbzm and the ncm_A chosen as a reference for ncm due to their lower energies from each pair of optimised gas molecules. The lattice energies of the SSs (E_{latt}) were computed with the following equation:

$$E_{\mathrm{latt,g}}^{\mathrm{SS}}[x_{\mathrm{g}}^{\mathrm{SS}}] = \frac{E_{cell}^{\mathrm{e}} - N_{\mathrm{h}}E_{\mathrm{h}}^{\mathrm{e}} - N_{\mathrm{g}}E_{\mathrm{g}}^{\mathrm{e}}}{N_{\mathrm{cell}}} \quad \text{Eq. 1}$$

where N_{cell}, N_{h} and N_{g} denote the total number of molecules present in the supercell, the number of host (bzm) molecules present and the number of guest (ncm or 3fbzm) molecules present in the supercell, respectively, and $E_{\mathrm{cell}}^{\mathrm{e}}$, $E_{\mathrm{bzm}}^{\mathrm{e}}$ and $E_{\mathrm{g}}^{\mathrm{e}}$ denote the electronic energy of the SS supercell along with bzm and lowest energy guest conformer in the gas phase, respectively.

Vibrational mode calculations

Vibrational mode calculations were performed for SS cells with x_g values of 0, 0.25, 0.5, 0.75 and 1.0, with all permutations of molecule replacement studied. TS-optimised structures from the final optimisation step were converted to supercells with unit cell parameters of at least 10 Å each, as recommended in literature.[18] As all supercells were constructed from cells roughly equal in size to a single cell of BZM-I or III (~5 x 5 x 20 Å), all cells were converted to 2 x 2 x 1 supercells.

Vibrational modes were calculated using the TS dispersion method, with tighter energy parameters than the four-step optimisation procedure (EDIFF=10^{-8} eV, EDIFFG=-10^{-6} eV Å^{-1}). Within VASP, the Hessian matrix and vibrational modes were calculated for the Γ-point using the finite-difference method (IBRION=6), where the magnitude of lattice displacements are controlled by the parameter POTIM. The lattice displacement was varied and finally set as 0.04 Å to ensure the only imaginary frequencies present were the three acoustic modes, and that the largest of these values was below 1.5 cm^{-1} per molecule. Some specific supercells required the displacement parameter to be varied slightly to eliminate additional imaginary frequencies. These cells were checked visually to ensure the remaining imaginary frequencies corresponded to the three acoustic modes. Imaginary frequencies could not be eliminated for the BZM-III cell with all molecules replaced with ncm_A despite numerous values of POTIM tested. A value of 0.04 Å was used for this cell, as this value performed best across the whole concentration range of supercells.

The obtained vibrational modes were converted into vibration contributions to the free energy (F_{vib}) by a Python code applying the following equation, approximating that harmonic phonons are non-interacting quantum harmonic oscillators with angular vibrational frequencies ω_i:[19]

$$F_{\mathrm{vib}}(T) = \frac{1}{2}\sum_i \hbar\omega_i + k_{\mathrm{B}}T\sum_i \ln\left(1 - \exp\left(\frac{-\hbar\omega_i}{k_{\mathrm{B}}T}\right)\right) \qquad \text{Eq. 2}$$

where $\hbar$ is the reduced Planck constant (1.055 x 10^{-34}), k_{B} is the Boltzmann constant (1.38 x 10^{-23} J / K) and T is the temperature in Kelvin. Values are summed across all vibrational modes i. The first term in this equation is the vibrational zero-point energy (ZPE) contribution to the free energy, whereas the second term encompasses the thermal contribution to the free energy and includes the vibrational entropic contribution to the free energy ($\mathrm{d}F_{\mathrm{vib}}/\mathrm{d}T$). To limit additional computational requirements for already expensive calculations, all SS cells from $0 < x_g < 0.25$ were instead fit to a polynomial trendline based on the values obtained for the

directly simulated cells. Trendlines were calculated with the NumPy Python library, using the Polyfit function with three polynomial coefficients.

To allow the direct comparison of temperature effects between SSs, the vibrational contributions to the free energy were computed relative to the BZM-I supercell substituted with ncm_A or $3fbzm_A$ ($\Delta F^{SS}_{vib,g}$). Meaning that at each x^{SS}_{g} (and each unit cell permutation), the BZM-I $guest_A$ unit cell is chosen as the reference energy state and set as the "zero" value for the temperature corrections. This process is analogous to the methods used when calculating relative lattice energies (ΔE_{latt}) for polymorphs of the same compound, given that at each guest concentration, the same reference states appear for each SS system (as the lowest energy conformer is always used). A SS system across values of x^{SS}_{g} can simply be thought of as a series of stable vs. metastable polymorphs competing in stability. This approach avoids the necessity of calculating vibrational frequencies for the gas phase reference molecules. In situations where POTIM is changed to eliminate imaginary frequencies, the same value for POTIM is used for the reference state calculation – meaning each value is always computed with a consistent reference point using the same energetic parameters.

Physical mixture energy calculations

The energies for the physical mixtures of bzm and ncm or 3fbzm were computed as a linear relationship between the lattice energies of the experimentally observed stable pure components. The lattice energies of the pure components were calculated with the same methodology as the SSs they are compared against, and all additional corrections were added where appropriate: disordered lattice energy averaging and entropy of mixing for pure disordered 3fbzm, plus temperature corrections for all systems. This ensures that the most accurate comparison can be made, as temperature effects will also apply to all pure systems, whereas entropy of mixing and disorder will still apply to pure but conformationally disordered solids like 3fbzm. Mixtures of a single SS with pure components were plotted by extrapolating from energy values at x_g to $x_g = 0$ and $x_g = 1$, while multi-SS mixtures can be plotted by linking SS energies at their respective x_g values, then extrapolation to the nearest pure component $x_g = 0$ and $x_g = 1$.

CSD Tools

The Packing Similarity Wizard in the CSD Mercury software was used for all crystal packing comparisons. Compared structures were added as refcodes directly from the CSD database. Clusters of 30 molecules were searched, with geometric distance tolerances of 20 % and angle tolerances of 20 degrees.

The molecule API within the CSD Python API was used to compute the molecular volumes for bzm, ncm and 3fbzm. The molecular_volume function was used on each refcode to obtain the molecular volume in $Å^3$.

1.4 Slurry Experiments

All slurry experiments were conducted using a total amount of solid mixture of 1.5 g and 3 g of IPA. The concentrations of 3fbzm relative to the total mass of solid load tested are detailed in Supplementary Table 1.4.1 and Supplementary Table 1.4.2. All samples underwent slurrying for a week using Polar Bear Plus Crystal at 25°C. The solutions were stirred continuously for 1 week with magnetic stirrer bars to ensure sufficient mixing and that solid-liquid equilibrium is achieved. After a week the slurries were filtered under vacuum and the resulting solid crystallites were immediately characterised with powder x-ray diffraction. An analogous methodology was followed for bzm:ncm and is described elsewhere.

The slurry of 12.5 wt.% 3fbzm, which showed the start of the conversion to BZM-III-SS, was repeated and slurried for a month to examine whether time would complete the conversion.

Slurry experiments were also attempted at other temperatures: 5 ℃, 50 ℃, 45 ℃, and 35 ℃. However, the slurries at temperatures higher than 25 ℃ could not be slurried for longer than several hours as precipitation of the solution on the walls started occurring. This occurred with decreased stirring rate too.

Supplementary Table 1.4.1. Concentrations of bzm and 3fbzm in the investigated slurries.

**Isopropanol (g)**		3				
**Total solid (g)**		1.5				
**Total Amount of Solution (g)**		4.5				
**bzm (wt.%)[a]**	**3fbzm (wt.%)[a]**	**Mass bzm (g)**	**Mass 3fbzm (g)**	**3fbzm (mol%) [a]**	**bzm (wt.% in solution) [b]**	**3fbzm (wt.% in solution)[b]**
95.0000	5.0000	1.4250	0.0750	4.3818	0.3167	0.0167
93.0000	7.0000	1.3950	0.1050	6.1505	0.3100	0.0233

90.0000	10.0000	1.3500	0.1500	8.8210	0.3000	0.0333
87.5000	12.5000	1.3125	0.1875	11.0625	0.2917	0.0417
85.0000	15.0000	1.2750	0.2250	13.3188	0.2833	0.0500
80.0000	20.0000	1.2000	0.3000	17.8762	0.2667	0.0667
70.0000	30.0000	1.0500	0.4500	27.1750	0.2333	0.1000
60.0000	40.0000	0.9000	0.6000	36.7275	0.2000	0.1333
50.0000	50.0000	0.7500	0.7500	46.5440	0.1667	0.1667
40.0000	60.0000	0.6000	0.9000	56.6357	0.1333	0.2000
30.0000	70.0000	0.4500	1.0500	67.0144	0.1000	0.2333
20.0000	80.0000	0.3000	1.2000	77.6924	0.0667	0.2667
10.0000	90.0000	0.1500	1.3500	88.6830	0.0333	0.3000
0.0000	100.0000	0.0000	1.5000	100.0000	0.0000	0.3333

[a] – wt.% with respect to total solid used

[b] – wt.% with respect to the total amount of solution, including solvent

Supplementary Table 1.4.1. Concentrations of bzm and 3fbzm in the investigated slurries.

**Isopropanol (g)**		3				
**Total solid (g)**		1.5				
**Total Amount of Solution (g)**		4.5				
**bzm (wt.%)[a]**	**3fbzm (wt.%)[a]**	**Mass bzm (g)**	**Mass 3fbzm (g)**	x_{3fbzm}^{slurry}	**bzm (wt.% in solution)[b]**	**3fbzm (wt.% in solution)[b]**
95.0000	5.0000	1.4250	0.0750	0.0438	0.3167	0.0167
93.0000	7.0000	1.3950	0.1050	0.0615	0.3100	0.0233
90.0000	10.0000	1.3500	0.1500	0.0882	0.3000	0.0333
87.5000	12.5000	1.3125	0.1875	0.1106	0.2917	0.0417
85.0000	15.0000	1.2750	0.2250	0.1332	0.2833	0.0500
80.0000	20.0000	1.2000	0.3000	0.1788	0.2667	0.0667
70.0000	30.0000	1.0500	0.4500	0.2718	0.2333	0.1000
60.0000	40.0000	0.9000	0.6000	0.3673	0.2000	0.1333
50.0000	50.0000	0.7500	0.7500	0.4654	0.1667	0.1667
40.0000	60.0000	0.6000	0.9000	0.5664	0.1333	0.2000
30.0000	70.0000	0.4500	1.0500	0.6701	0.1000	0.2333
20.0000	80.0000	0.3000	1.2000	0.7769	0.0667	0.2667
10.0000	90.0000	0.1500	1.3500	0.8868	0.0333	0.3000
0.0000	100.0000	0.0000	1.5000	1.0000	0.0000	0.3333

1.5 Crash Cooling Experiments

Crash cooling crystallisation of supersaturated solution of bzm in IPA was conducted at with a supersaturation ratio of 1.6. A stirred supersaturated solution was heated up to approximately 50 °C until complete dissolution. The solution was then quickly cooled down to 25 °C using a LAUDA ECO RE 415 water bath. Once the solution was cooled down it took few minutes for the crystals to be noticed by human eye. Once this occurred, the crystals were filtered and were retrieved immediately to prevent the conversion of BZM-III to BZM-I. The resulting crystals were filtered, dried, and characterised via SCXRD, optical microscopy and PXRD.

Crash cooling crystallisation was also utilised for preparation of solid solution crystals: rapid cooling from 60℃, where full dissolution was achieved, to 25℃ was conducted followed by 1 week equilibration at this temperature. Two sets of slurries were conducted: one involving stirring at the equilibration and one not involving stirring to ensure good quality diffraction crystals could be obtained for SCXRD. After 1 week of isothermal suspension, slurries were filtered via vacuum Büchner filtration and dried in vacuum oven at 40 ℃.

1.6 Characterisation Techniques

1.6.1 Powder X-ray Diffraction (PXRD) measurements

PXRD patterns for the different samples were recorded using a Bruker D2 Phaser diffractometer equipped with a LYNXEYE detector, using a Cu-Kα radiation (λ= 1.54 Å). Intensity data were recorded in the 2θ range of 5-40° (time 0.3 s, increment 0. 018°).

1.6.2 Differential Scanning Calorimetry (DSC) measurements

DSC measurements were performed using a TA instruments DSC 2500 calorimeter. Approximately 4 - 5 mg of each sample was placed into an aluminium sealed pan (type TzeroAluminum) and the measurement was carried out under N atmosphere, in the range of 40 °C to 160 °C with a heating ramp of 10 °C min^{-1}. The calorimeter was calibrated both for temperature and sensitivity using DSC standards. Data were analysed using the TA TRIOS software.

1.6.3 Nuclear Magnetic Resonance (NMR) measurements

NMR measurements were undertaken to estimate the incorporation of 3fbzm in bzm crystal lattice during slurry and crash cooling experiments. The 3fbzm content in such samples was detected by 1 H−NMR measurements (128 scans per sample) carried out using a 400 MHz NMR spectrometer (Bruker). Deuterated acetone was used in the NMR samples to dissolve the crystals used for analysis. The proton NMR scans were recorded and analysed using MestReNova version 14.1.0−24037.[20] The same procedure was followed for bzm:ncm.

1.6.4 Optical Microscopy

Samples from slurries and crash cooling were analysed using Zeiss Axioplan 2 microscope and images were obtained using the INFINITY Analyse and Capture Software version 6.5.6.

1.6.5 Single Crystal X-ray Diffraction (SCXRD) Measurements

The data for the BZM-III SSs crystals were collected at 100 K and 150 K using an Oxford Cryostream 800 Plus cooling device on a four-circle XtaLAB AFC11 (RINC): Kappa single diffractometer equipped with Hybrid Pixel Array Detector using Cu Kα (λ = 1.54 Å) radiation. Data reduction and face indexing of both samples were performed with the CrysAlisPro software (version 1.171.36.32).[21] OLEX2 (version 1.2)[22] was used to solve and refine the crystal structures by employing direct methods and the least-squares procedure using SHELXS program and SHELXL package, respectively.[23,24] The refinement was based on F^2 for all reflections except those with negative intensities. Weighted R factors (*w*R) and all goodness-of-fit (S) values were based on F^2, whereas conventional R factors were based on the amplitudes, with F set to zero for negative F^2. Scattering factors were taken from the International Tables for Crystallography.[25] Crystal data, data collection, and refinement details are summarized in the Supplementary Tables 2.7.1 – 2.7.3.

All non-hydrogen atoms were refined anisotropically. Initially the SS sample was solved as pure bzm to examine the difference in parameters. Disorder was confirmed by analysis of the anisotropic and isotropic displacements parameters and the electron density peaks. Once disorder was confirmed, the nitrogen/carbon disorder in bzm:ncm solid solutions was refined by dividing the disordered atoms into three groups using "PART" command. The atoms were linked with the free variable "FVAR". To sum up occupancies of all disordered parts to 1, the "SUMP" command with the esd = 0.001 was applied. If necessary, the positions and ADPs of split atoms were constrained with the "EXYZ" and "EADP" command, respectively. Similar procedure was followed for bzm:3fbzm, with an exception that F and H atoms instead of N and C atoms were disordered. Occupancy of three disordered parts of bzm:3fbzm sum up to 1.

1.6.6 Solid-State NMR

All details pertaining to solid-state NMR (ssNMR) experiments are discussed in the main manuscript.

Supplementary Results

2.1 Identification of polymorphs

bzm

The crystalline structures of bzm powders/crystals were identified by comparing the experimental PXRD patterns with the calculated patterns from the single crystal structures obtained from the CSD: BZAMID05 (BZM-I)[1] and BZAMID08 (BZM-III)[2].

3fbzm

The crystalline structure of commercial 3fbzm powder was confirmed by comparing the experimental PXRD pattern with the calculated patterns from the single crystal structures obtained from CSD: BENAFM11 (form I)[15].

2.2 Crystal packing comparison

Supplementary Table 2.2.1 shows the results of the crystal packing similarity study undertaken with the Packing similarity Wizard in Mercury comparing polymorphs of bzm to the only reported polymorph of 3fbzm. Supplementary Table 2.2.2 shows the results of the crystal packing comparison of bzm with the reported stable form of ncm, respectively.

Supplementary Table 2.2.1. Results of the crystal packing comparison (Packing Similarity Wizard in Mercury).

bzm polymorphs	Crystal packing similarity[d]
I[a]	18/30[60%]
II[b]	6/30 [20%]
III[c]	30/30 [100%]

Supplementary Table 2.2.2. Further results of the crystal packing comparison (Packing Similarity Wizard in Mercury).

bzm polymorphs	ncm polymorphs	Crystal packing similarity
I	I[e]	1/30 [3.33%]
III	I	1/30 [3.33%]

[a] BZAMID05
[b] BZAMID06
[c] BZAMID08
[d] No. of similar molecules out of 30
[e] NICOAM05

2.3 LAG experiments

Supplementary Figures 2.3.1 shows PXRD patterns of the bzm:3fbzm system prepared by LAG at 3fbzm concentrations in the ranges of 5-100 (wt.%). Starting from 20 wt.% 3fbzm PXRD patterns show a good fit with that of BZM-III, calculated from the single-crystal structure (BZAMID08[2]). At 5 and 10 wt.% 3fbzm the pattern corresponds to BZM-I, and the shift in

peaks suggests a solid solution formation at these concentrations too. LAG results for bzm:ncm are reported elsewhere.[1]

Supplementary Figure 2.3.2 shows shifts of the first 6 PXRD peaks as the concentration of 3fbzm increases, with the planes causing the most prominent peak shifts studied in the main manuscript shown in Supplementary Figure 2.3.3. Supplementary Figure 2.3.4 demonstrates the thermographs of the LAG powder samples. These results confirmed solid solution formation.

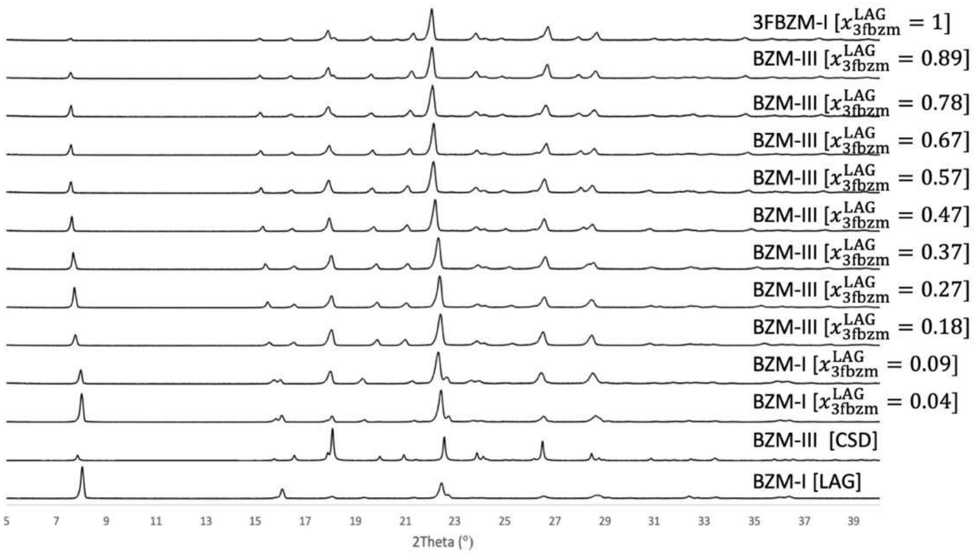

Supplementary Figure 2.3.1. PXRD patterns of LAG products containing various concentrations of 3fbzm (LAG with isopropanol).

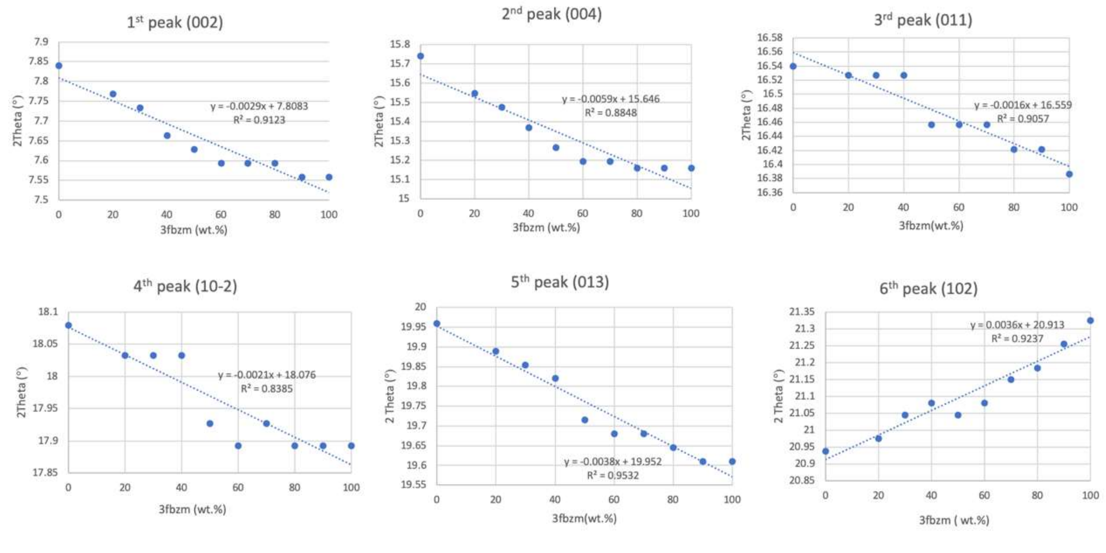

Supplementary Figure 2.3.2 a. Peak shifts of the first 6 peaks identified from PXRD patterns obtained from LAG samples with isopropanol for bzm:3fbzm.

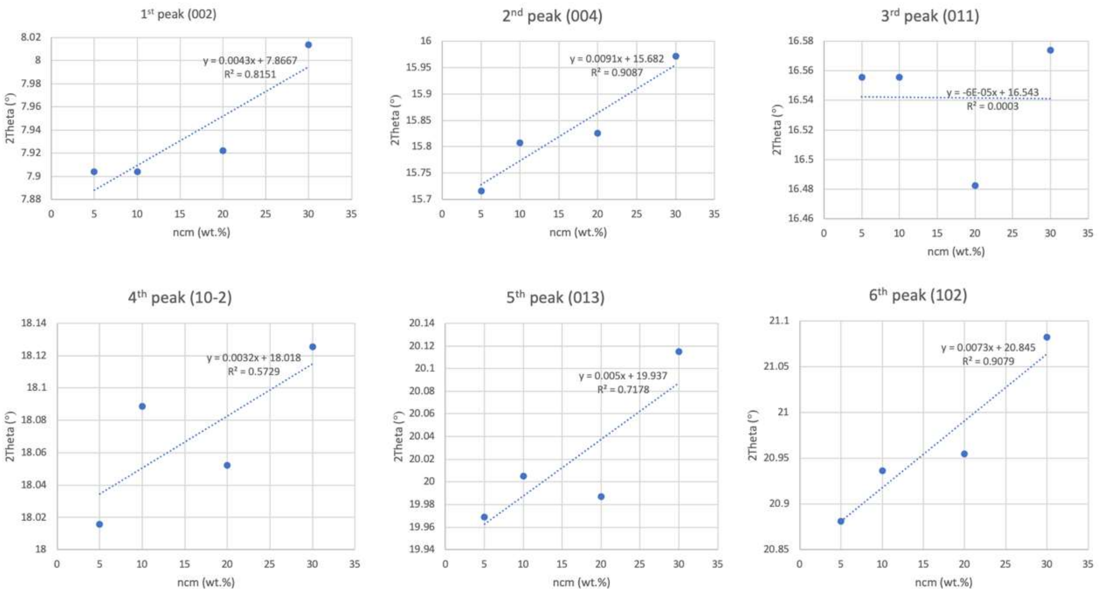

Supplementary Figure 2.3.2 b. Peak shifts of the first 6 peaks identified from PXRD patterns obtained from LAG samples with isopropanol for bzm:ncm system.

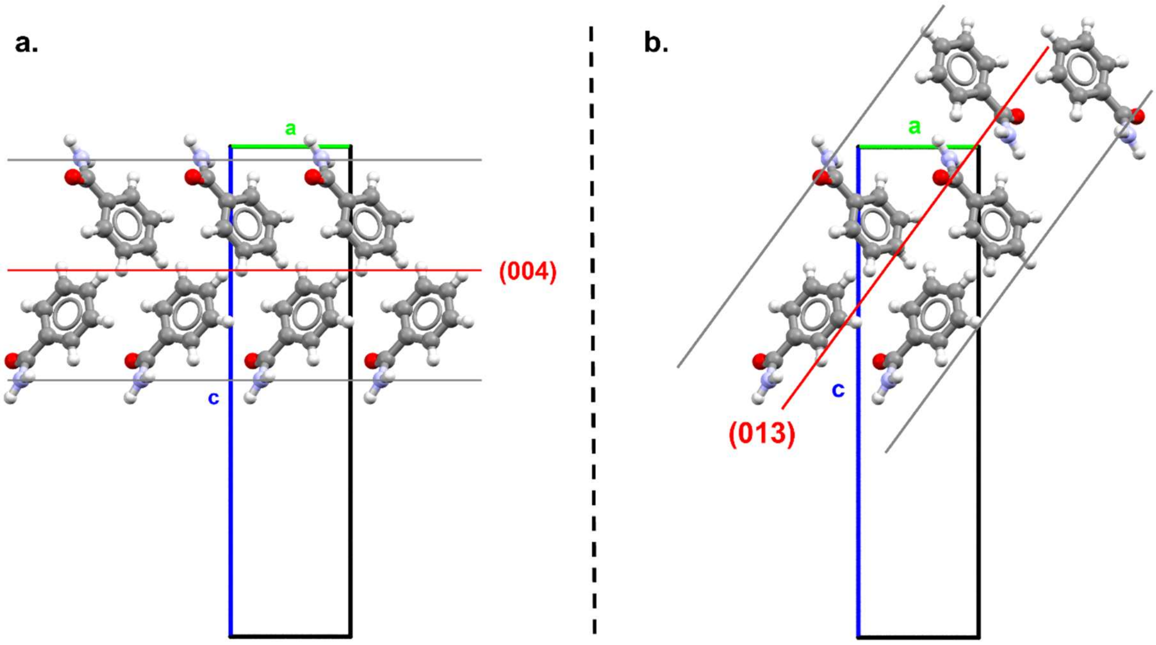

Supplementary Figure 2.3.3. Schematics of the planes studied in the manuscript from the pure BZM-III structure (BZAMID08) showing the most prominent peak shifts in a. the bzm:ncm system and b. the bzm:3fbzm system. Planes are shown in red and labelled by their hkl index, while the bounds of the planes are shown in grey.

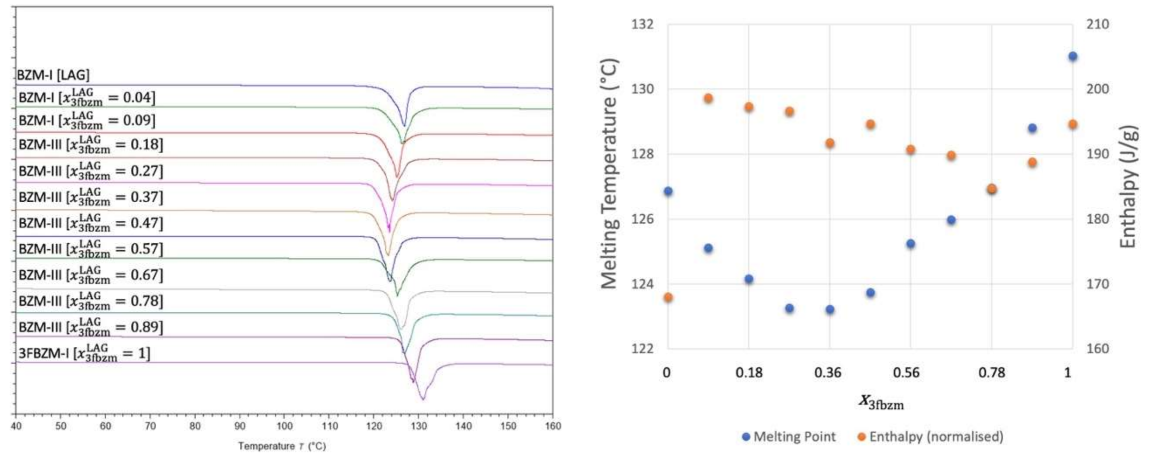

Supplementary Figure 2.3.4 a. DSC thermographs of LAG powder samples at different 3fbzm concentrations. A minimum occurs in the melting temperature corresponding to approximately 30 wt.% of 3fbzm.

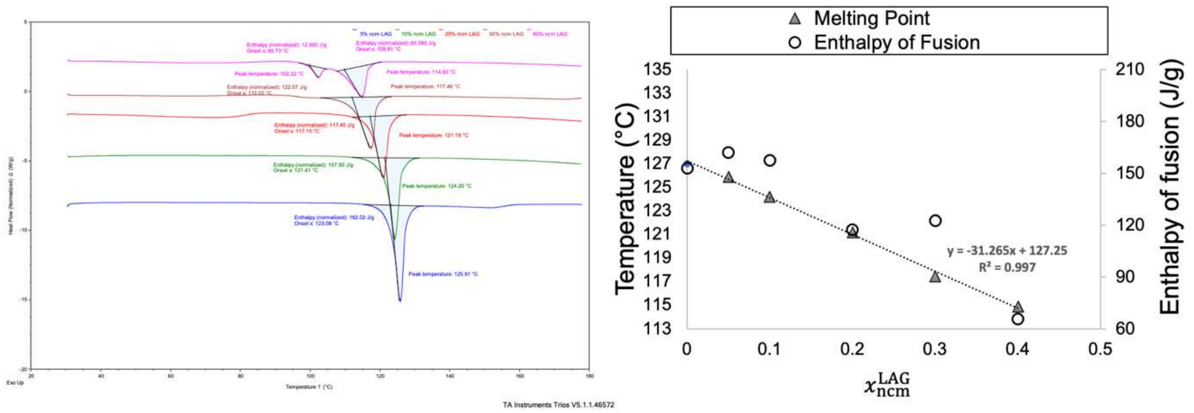

Supplementary Figure 2.3.4 b. DSC thermographs of LAG powder samples at different ncm concentrations. A linear decreasing correlation can be noticed between the concentration of ncm and melting temperatures. At approx. 0.3 ncm two melting events can be seen.

2.4 Physical Mixtures

For further confirmation of solid solution formation, physical mixtures at different bzm:3fbzm rations were prepared. Manual sample weighing and gentle spatula stirring of the samples were conducted. This was followed by obtaining PXRD diffractograms and DSC thermographs of the samples. Both are shown in Supplementary Figures 2.4.1 and 2.4.2. PXRD patterns show multiple phases present, with different patterns to those obtained from LAG. DSC thermographs of LAG crystals and physical mixtures are similar; however, the melting temperatures are different. Slower heating ratios were examined to see whether the two melting

events could be distinguished on the thermograms. These are shown in Supplementary Figure 2.4.3. Results for manual physical mixtures for bzm:ncm are reported elsewhere.[1]

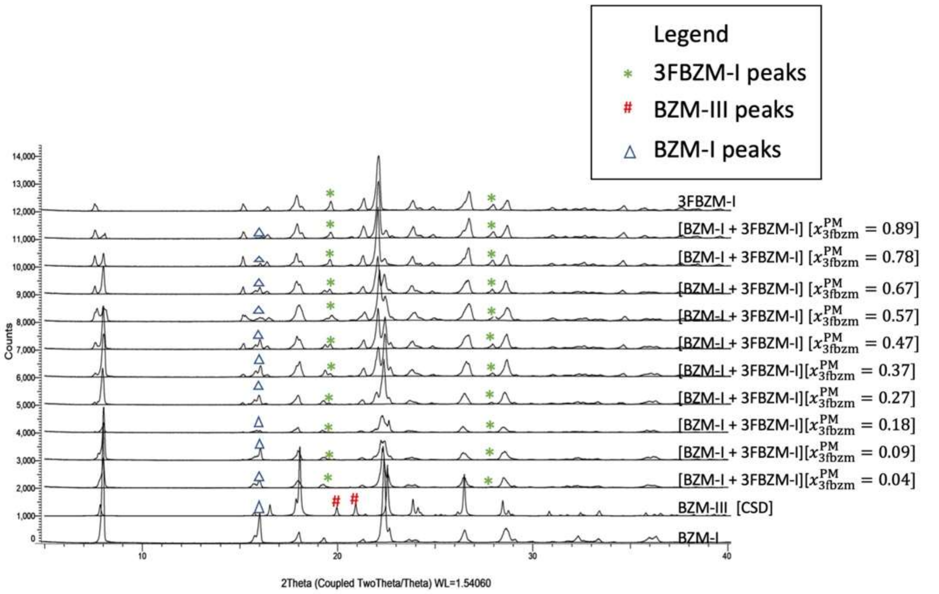

Supplementary Figure 2.4.1. PXRD patterns of physical mixtures.

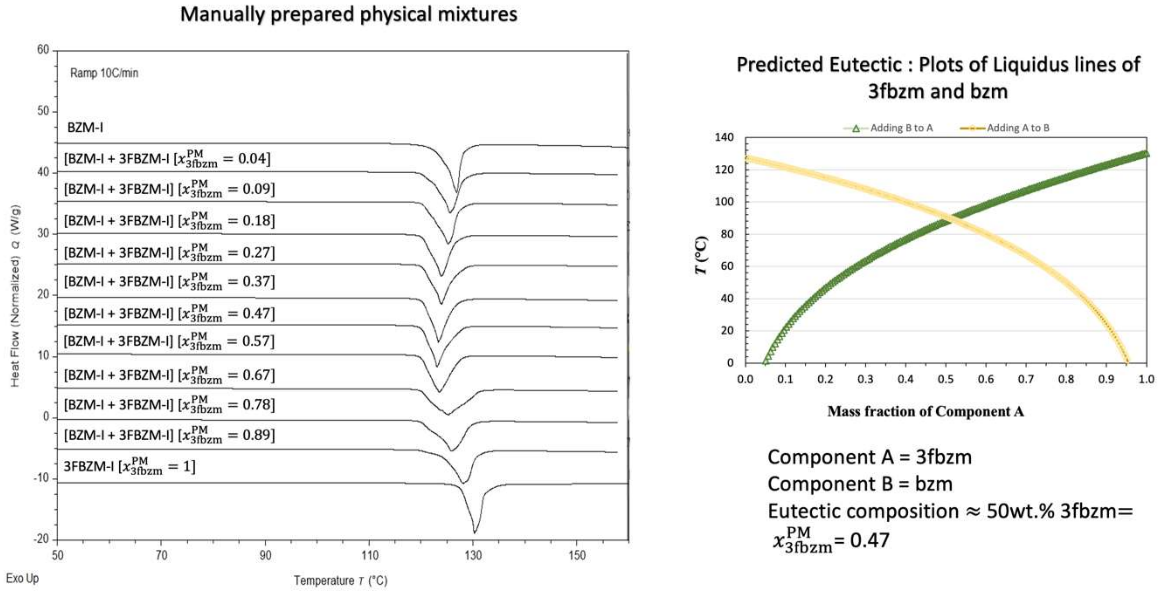

Supplementary Figure 2.4.2. DSC thermograms of physical mixtures and predicted eutectic point using Schroder-van Laar equation.

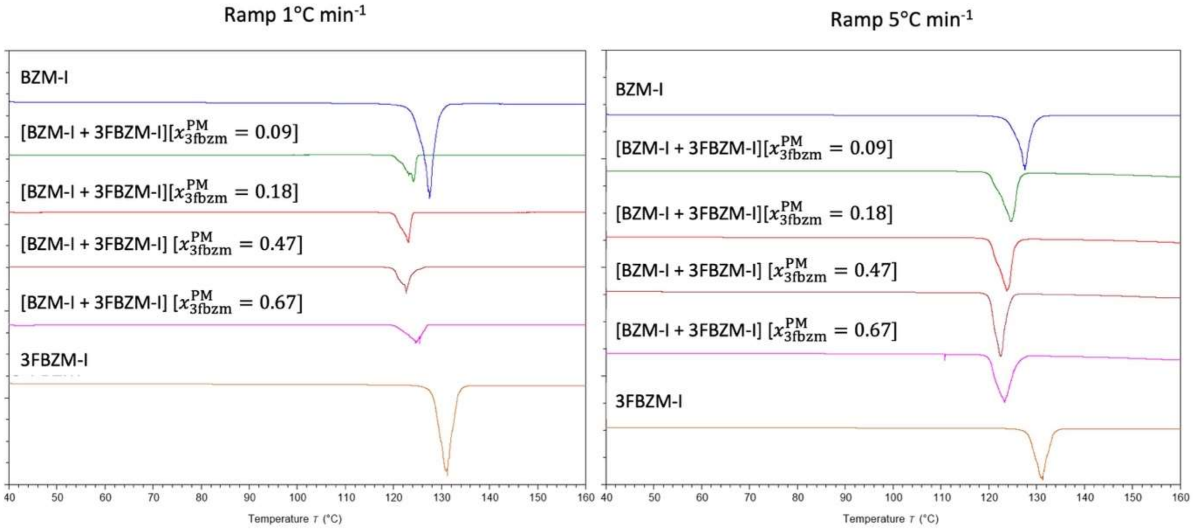

Supplementary Figure 2.4.3. DSC thermograms of physical mixtures at lower heating rates.

2.5 Slurry Experiments

- 25°C, 1 week, IPA

Supplementary Figure 2.5.1 shows the PXRD patterns of samples obtained by slurrying excess solids of BZM-I in the presence of x_{3fbzm}^{slurry} in the range of 0.04 to 1. Supplementary Figure 2.5.2 shows PXRD patterns of slurry crystallites when the concentrations of 3fbzm present in the excess solids at the start of the slurries were narrowed down to x_{3fbzm}^{slurry} = 0.09, 0.11 and 0.13. Supplementary Figure 2.5.3 illustrates the PXRD patterns when 3fbzm is lower than 0.09. The results show that at these conditions the conversion to BZM-III-SS starts at $x_{3fbzm}^{slurry} = 0.11$. Results for bzm:ncm slurries are reported elsewhere.[1]

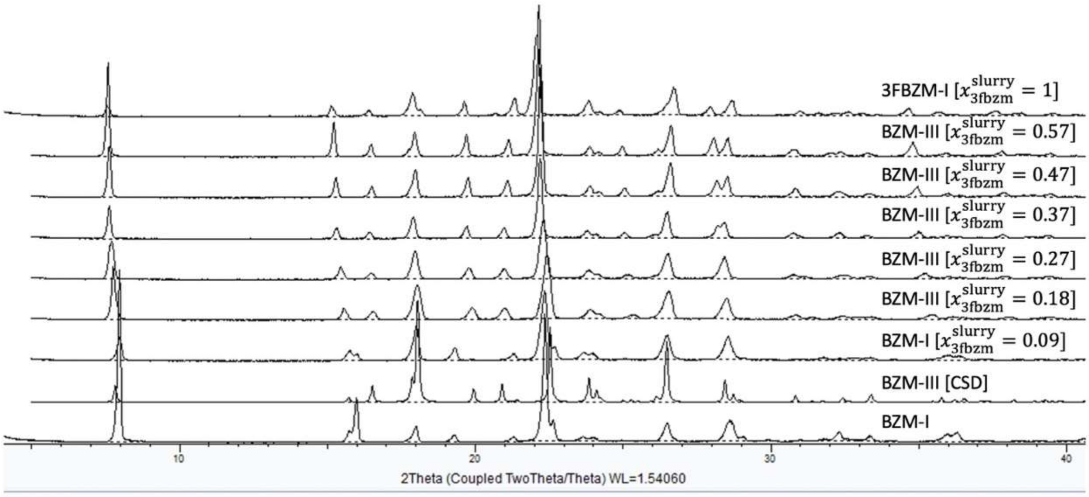

Supplementary Figure 2.5.1. PXRD patterns of slurry products obtained from slurrying excess solid mixtures of bzm and 3fbzm in IPA at different 3fbzm doping levels.

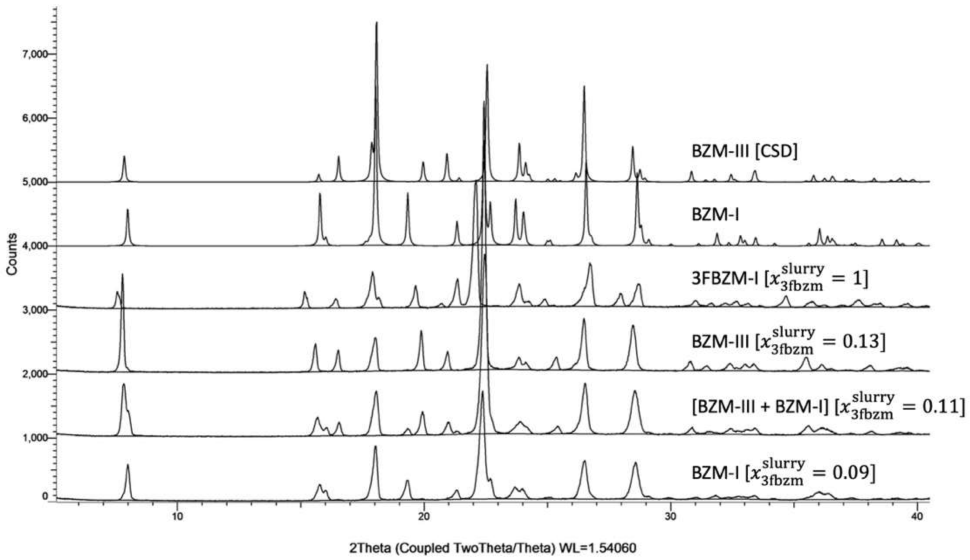

Supplementary Figure 2.5.2. PXRD patterns of slurry crystallites obtained from excess solid mixtures of bzm and 3fbzm in IPA at 10, 12.5 and 15 wt.% 3fbzm doping levels.

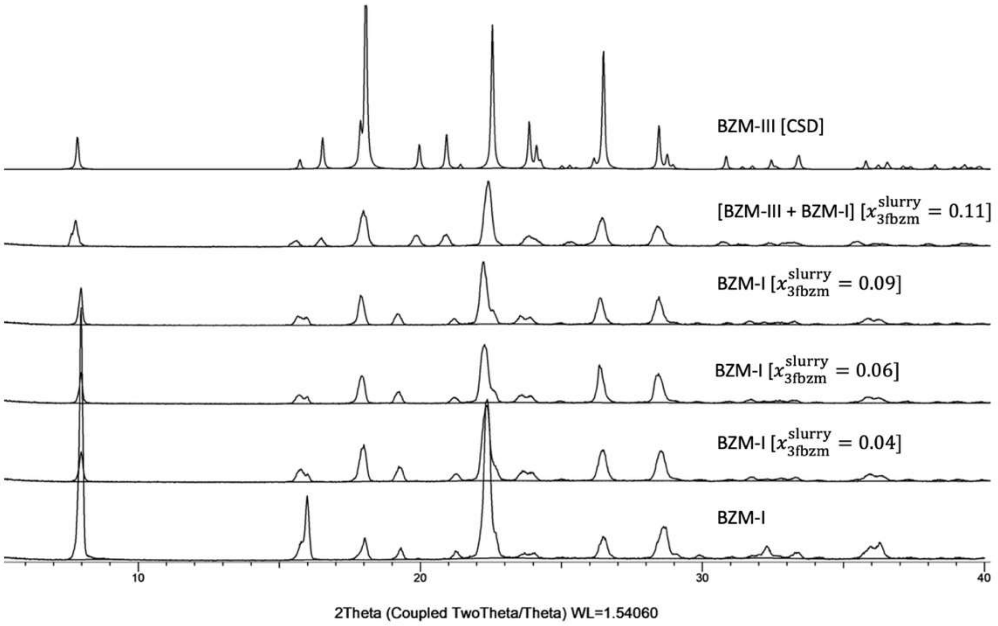

Supplementary Figure 2.5.3. PXRD patterns of dried slurry crystallites obtained from mixtures of bzm and 3fbzm in IPA at 12.5wt.%, 10wt.%, 7wt.% and 5wt.% 3fbzm doping level.

- 12.5 wt.% 3fbzm 1 month vs 1 week slurry in IPA at 25℃

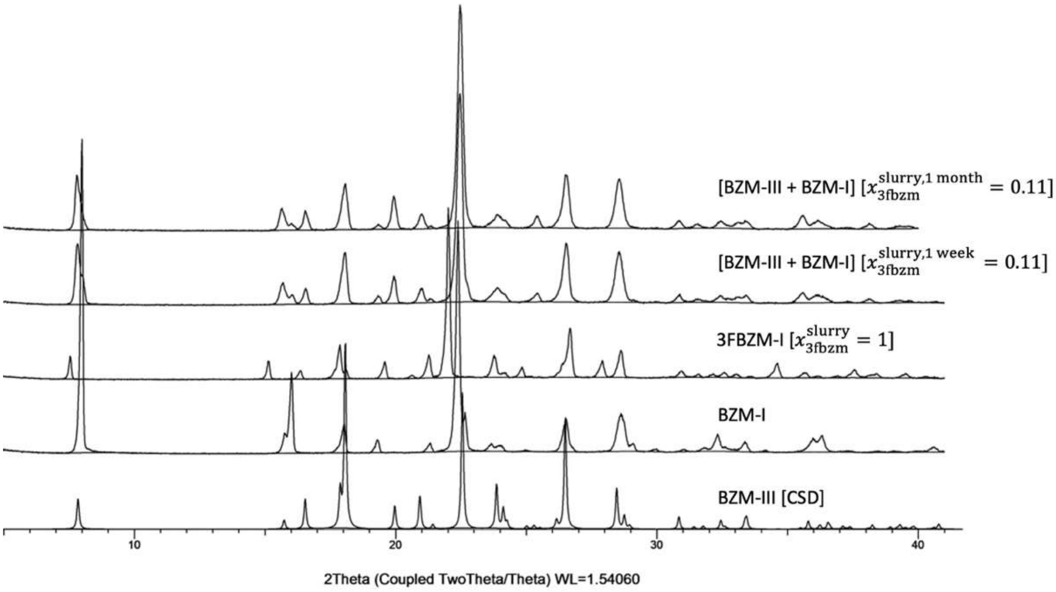

Supplementary Figure 2.5.4. PXRD patterns of slurry crystallites obtained from mixtures of bzm and 3fbzm in IPA at 12.5wt.% 3fbzm.

- Slurries at other temperatures

i. 5 ºC, 1 week, IPA, 12.5 wt.% 3fbzm.

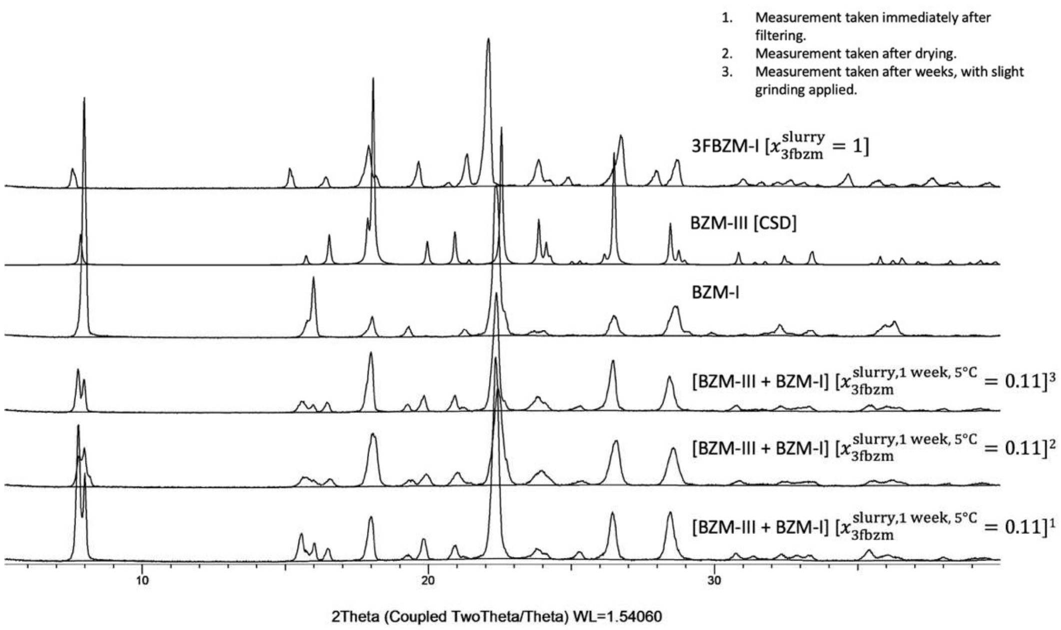

Supplementary Figure 2.5.5. PXRD patterns of dried slurry crystallites obtained from excess solids of bzm and 3fbzm in IPA at 12.5wt.% doping level and 5°C.

ii. Slurries at temperatures above 25°C

Slurries at temperatures of 30°C, 40°C, 45°C and 50°C were attempted. However, they proved problematic due to precipitation of solids on the walls of the flask after slurring for longer than 4 hours. Decreasing the stirring rate didn't stop the precipitation so slurries were repeated and only slurried for 4hrs. At 40°C slurrying for 4hr resulted in crystallisation of BZM-I and III solid solutions together.

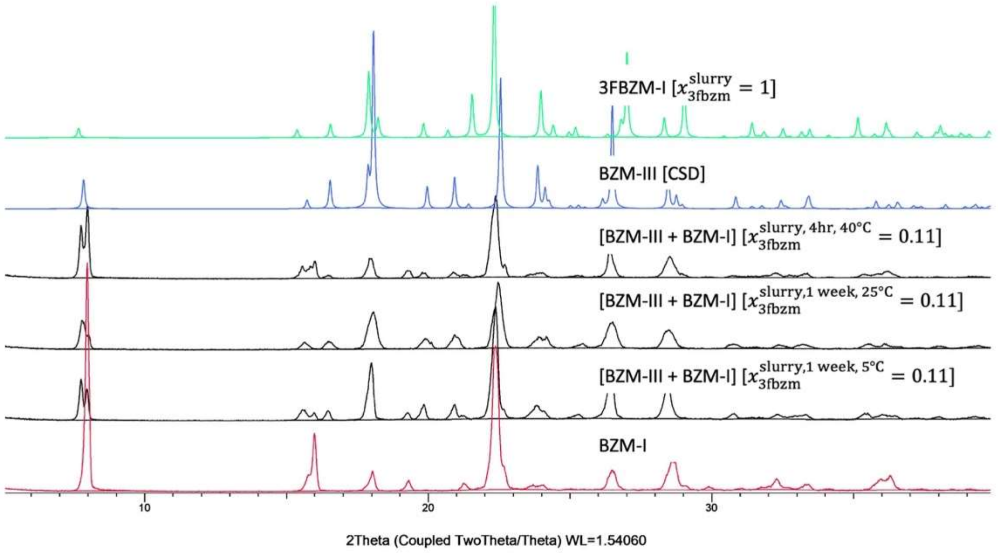

Supplementary Figure 2.5.6. Comparison of PXRD patterns of slurry crystallites of bzm:3fbzm obtained from different temperatures. 5°C and 25°C slurries were conducted for 1 week.

Slurry crystallites were also characterised by DSC. The DSC traces are stacked and compared to those of bzm:ncm crystallites in Supplementary Figure 2.5.7.

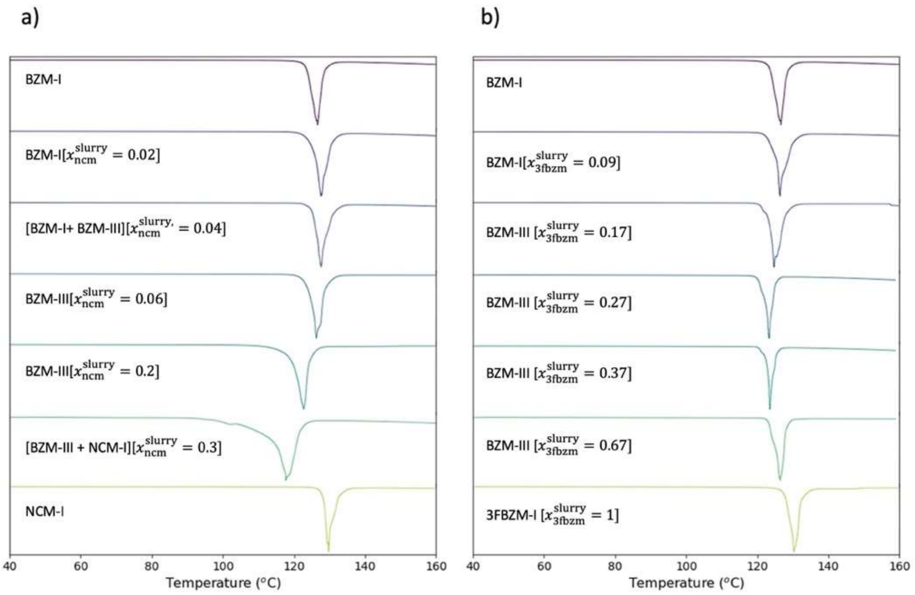

Supplementary Figure 2.5.7. Comparison of DSC thermograms of slurry crystallites of bzm:ncm (a)) vs bzm:3fbzm (b)) system. Continuous solid solution is observed in bzm:3fbzm system and a partial solid solution in bzm:ncm system

2.6 Unit Cell Parameters

Unit cell parameters for the bzm:3fbzm and bzm: ncm SSs systems were calculated using the PXRD patterns of LAG samples. The following assumptions were made in these calculations:

- PXRD peaks of solid solutions were indexed as for pure BZM-III (planes were assumed to be the same despite shift in peak positions).
- The crystal system of the solid solution was assumed to be monoclinic (the same as for BZM-III).
- β was assumed to be the same as for BZM-III - 101.2914°.

The results are demonstrated in Supplementary Figure 2.6.1 and 2.6.2

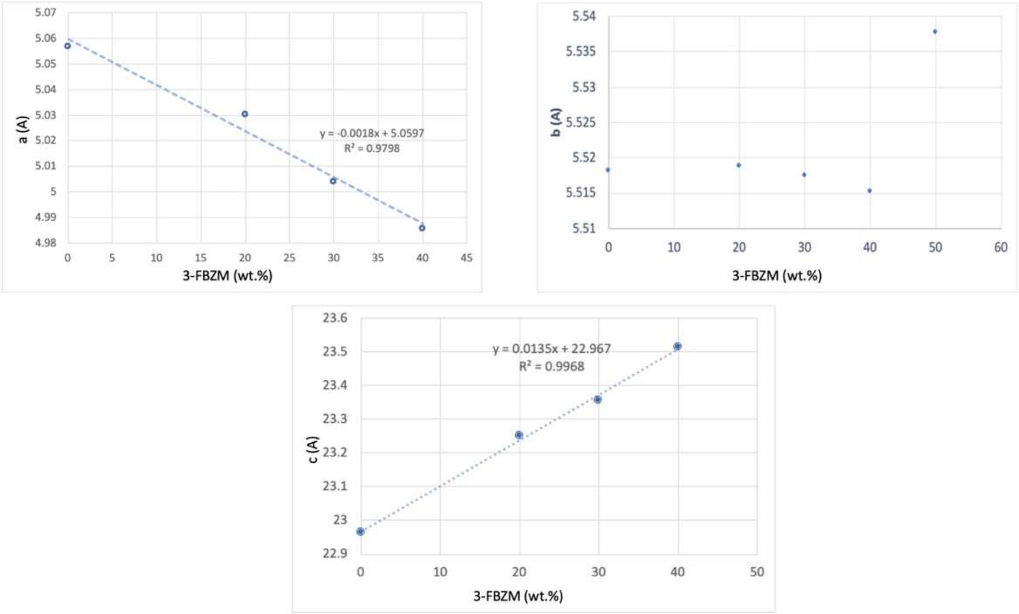

Supplementary Figure 2.6.1. Change of unit cell dimensions with 3fbzm incorporation.

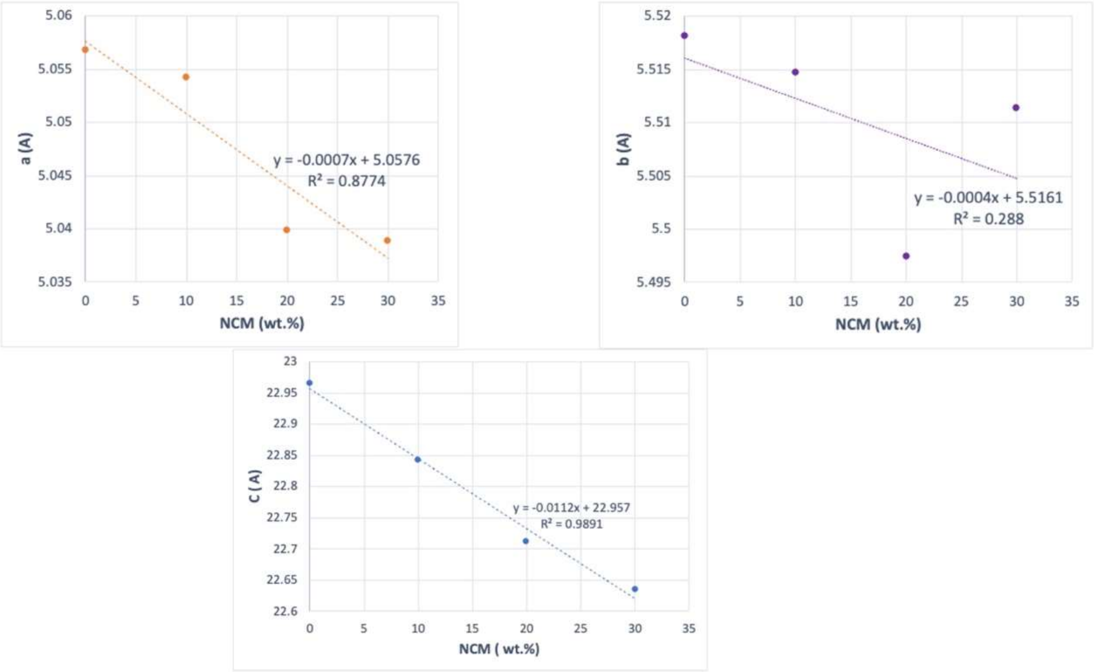

Supplementary Figure 2.6.2. Change of unit cell dimensions with ncm incorporation.

As illustrated in Supplementary Figures 2.6.1 and 2.6.2:

- on average an increase along c and a decrease along a and b are observed with increasing 3flbzm.
- bzm:ncm showed decrease in all unit cell parameters as ncm was increased.

- for bzm:ncm the biggest & most linear decrease was found along c, following by a, and then following by b.

2.7 SCXRD

Details of SCXRD experiments are described in the Section 1.6.5.

Supplementary Table 2.7.1 BZM-III crystal structures

	1 (racc34)	2 (racc8)
**Crystal Data**		
**Chemical Formula**	C_7H_7NO	
**Mr**	121.14	
**Crystal system**	monoclinic	
**Space Group**	$P2_1/c$	
**Temperature (K)**	100	150
**a, b, c (Å)**	a = 5.04249(19)<br>b = 5.42960(19)<br>c = 22.6391(9)	a = 5.04811(18)<br>b = 5.44651(19)<br>c = 22.7594(8)
**V (Å)^3**	601.95(4)	607.54(4)
**Z**	4	
**Z'**	1	
**Radiation type**	Cu Kα	
**μ(mm^{-1})**	0.738	0.731
**Crystal size (mm)**	0.308 × 0.098 × 0.054	0.039 × 0.072 × 0.360
**Data Collection**		
**Diffractometer**	XtaLAB AFC11 (RINC): Kappa single	
**T_{min}, T_{max}**	0.592, 1.000	0.730, 1.000
**Number of measured, independent and observed [I > 2σ(I)] reflections**	3326,<br>1073	2349,<br>1196
**R_{int}**	0.0268	0.0211
**Refinement**		
$R [F^2 > 2\sigma(F^2)], \omega R(F^2), S$	0.0441<br>0.1216<br>1.076	0.0455<br>0.1433<br>1.218

**No. of reflections**	1073	1196
**No. of parameters**	82	82
**Number of restraints**	0	0
**H atoms treatment**	H atoms treated by constrained refinement	
**$\Delta\rho_{max}$, $\Delta\rho_{min}$ (e Å^{-3})**	0.226, -0.208	0.153, -0.322
**CCDC entry**	2253776	2253775

Supplementary Table 2.7.2 BZM-III [bzm:ncm] crystal structures:

	3 (racc49)	4 (racc50)
**Crystal Data**		
**Chemical Formula**	$C_{6.84}H_{6.84}N_{1.15}O$	$C_{6.8}H_{6.8}N_{1.2}O$
**Molecular weight**	121.25	121.36
**Crystal system**	monoclinic	
**Space Group**	$P2_1/c$	$P2_1/c$
**Temperature (K)**	100	100
**a, b, c (Å)**	a = 5.04462(13)<br>b = 5.42932(12)<br>c = 22.4992(7)	a = 5.04645(15)<br>b = 5.43257(15)<br>c = 22.4512(7)
**V (Å)^3**	598.46(3)	597.8
**Z**	4	
**Z'**	1	
**Radiation type**	Cu Kα	
**μ(mm^{-1})**	0.751	0.754
**Crystal size (mm)**	0.074 x 0.166 x 0.199	0.215 × 0.143 × 0.096
**Data Collection**		
**Diffractometer**	XtaLAB AFC11 (RINC): Kappa single	
**T_{min}, T_{max}**	0.740, 1.000	0.798, 1.000
**Number of measured, independent and observed [$I > 2\sigma(I)$] reflections**	4179,<br>1195	4655,<br>1224
**R_{int}**	0.0294	0.0341
**Refinement**		
R [$F^2 > 2\sigma(F^2)$], $\omega R(F^2)$, S	0.0438<br>0.1152<br>1.046	0.0600<br>0.1623<br>1.077

**No. of reflections**	1195	1224
**No. of parameters**	97	97
**Number of restraints**	25	37
**H atoms treatment**	H atoms treated by a mixture of independent and constrained refinement	
**$\Delta\rho_{max}$, $\Delta\rho_{min}$ (e Å^{-3})**	0.175, -0.319	0.347, -0.273
**CCDC entry**	2253556	2253557

Supplementary Table 2.7.3 BZM-III [bzm:3fbzm] – racc44, racc45, and BZM-I[bzm:3fbzm] -racc53 crystal structures:

	5 (racc44)	6 (racc45)	7 (racc53)
**Crystal Data**			
**Chemical Formula**	$C_7H_{6.9}F_{0.1}NO$	$C_7H_{6.56}F_{0.44}NO$	$C_7H_{6.96}F_{0.04}NO$
**Mr**	122.93	129.05	121.90
**Crystal system**	monoclinic		
**Space Group**	$P2_1/c$		
**Temperature (K)**	150	150	100
**a, b, c (Å)**	a = 5.0416(2)<br>b = 5.44840(10)<br>c = 22.9244(8)	a = 5.0248(2)<br>b = 5.4441(2)<br>c = 23.4319(11)	a = 5.55040(10)<br>b = 5.03440(10)<br>c = 21.5501(5)
**V (Å)^3**	612.72 (4)	624.27(5)	602.12(2)
**Z**	4		
**Z'**	1		
**Radiation type**	Cu Kα		
**μ(mm^{-1})**	0.758	0.852	0.752
**Crystal size (mm)**	0.0054x 0.076x 0.176	0.04 × 0.068 × 0.081	0.12x 0.19x 0.26
**Data Collection**			
**Diffractometer**	XtaLAB AFC11 (RINC): Kappa single		
**T_{min}, T_{max}**	0.77606, 1.0	0.83127,1.0	0.60826, 1.0
**Number of measured, independent and observed [I > 2σ(I)] reflections**	4494,<br>1240	2456,<br>1116	4458,<br>1224
**R_{int}**	0.0358	0.0208	0.0325
**Refinement**			
$R [F^2 > 2\sigma(F^2)], \omega R(F^2), S$	0.0509	0.0483	0.0423

	0.1279<br>1.049	0.1105<br>1.045	0.1087<br>1.041
**No. of reflections**	1240	1116	1224
**No. of parameters**	104	111	106
**Number of restraints**	26	14	19
**H atoms treatment**	H atoms treated by a mixture of independent and constrained refinement		
**$\Delta\rho_{max}$, $\Delta\rho_{min}$ (e Å^{-3})**	0.196, -0.211	-0.168, 0.165	0.170, -0.284
**CCDC entry**	2253772	2253773	2253774

The unit cell dimensions obtained from SCXRD mostly agree with the unit cell parameter calculations shown in section 2.6.

2.8 Optical microscopy images

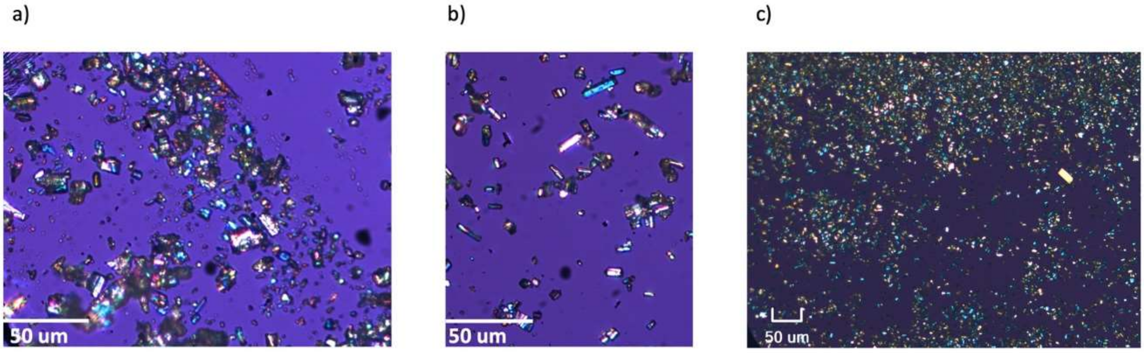

Supplementary Figure 2.8.1. Optical microscope images of slurry crystallites of bzm:3fbzm for a) BZM-I[x_{3fbzm}^{slurry} = 0.04], b) [BZM-III+BZM-I][x_{3fbzm}^{slurry} = 0.11],c) BZM-IIIx_{3fbzm}^{slurry} = 0.27]. Optical microscopy images of bzm:ncm slurry crystallites are reported elsewhere.[1] SSs crystallites of BZM-III structure have plate like morphology instead of the usual needle like pure BZM-III morphology.

2.9 Supplementary ssNMR spectra

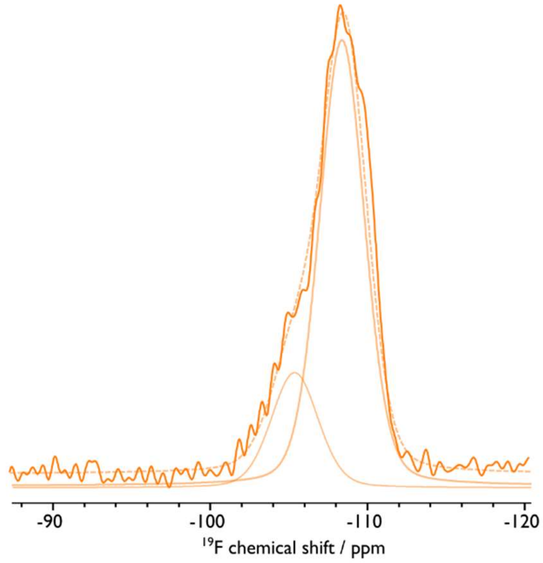

Supplementary Figure 2.9.1. ^{19}F ss-NMR spectrum of pure 3FBZM-I. Signal is deconvoluted into the signals from conformers A and B (dashed lines) with a ratio of 0.3:0.7. The orange colouring denotes that the crystal structure adopted is that of BZM-III or 3FBZM-I

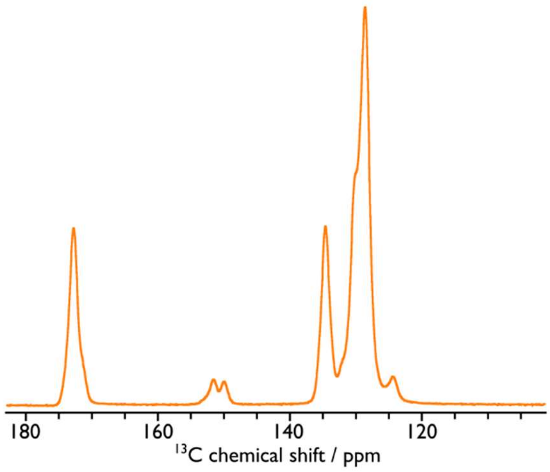

Supplementary Figure 2.9.2. Full-range ^{13}C ss-NMR spectrum of the BZM-III-SS formed with ncm shown in the main manuscript (Figure 9). The orange colouring denotes that the crystal structure adopted is that of BZM-III or 3FBZM-I. The main figure focussed on the peaks between 155 and 145 ppm.

Supplementary Computational Results

3.1 Impact of Dispersion Corrections

All plotted SS points on the below charts denote the mean energy from all cell permutations with the same value of x_g^{SS}.

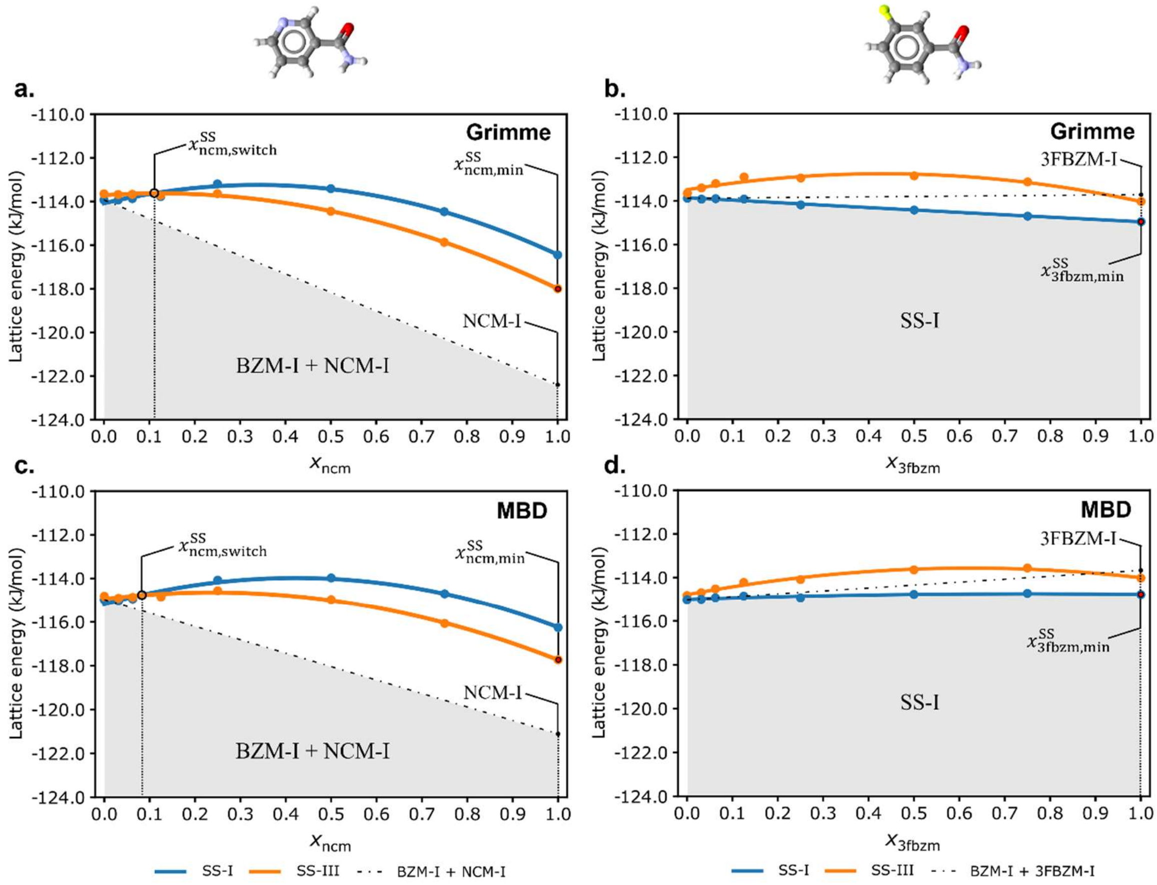

Supplementary Figure 3.1.1: The impact of improved dispersion corrections from Grimme-D2 corrections to many-body dispersion (MBD) corrections on the lattice energies of SSs incorporating lowest energy conformers - ncm_B (a and c) and $3fbzm_A$ (BZM-I) and $3fbzm_B$ (BZM-III) (b and d) incorporating into the BZM-I (blue) and BZM-III (orange) lattices. The overall energetic trends remain the same with some slight changes in crossover concentrations between the conformers. Physical mixtures energies of the two stable pure components (BZM-I + NCM-I and BZM-I + 3FBZM-I) in each system are shown by black dash-dotted lines, while the minimum energy SS ($x_{g,min}^{SS}$) is shown by a red point. Energies of the pure guest structures (NCM-I and 3FBZM-I) are labelled, as well as $x_{ncm,switch}^{SS}$. The lowest energy phase or mixture is labelled and shaded for clarity, with SS-I denoting a SS with the BZM-I structure.

3.2 Impact of Mixing Entropy

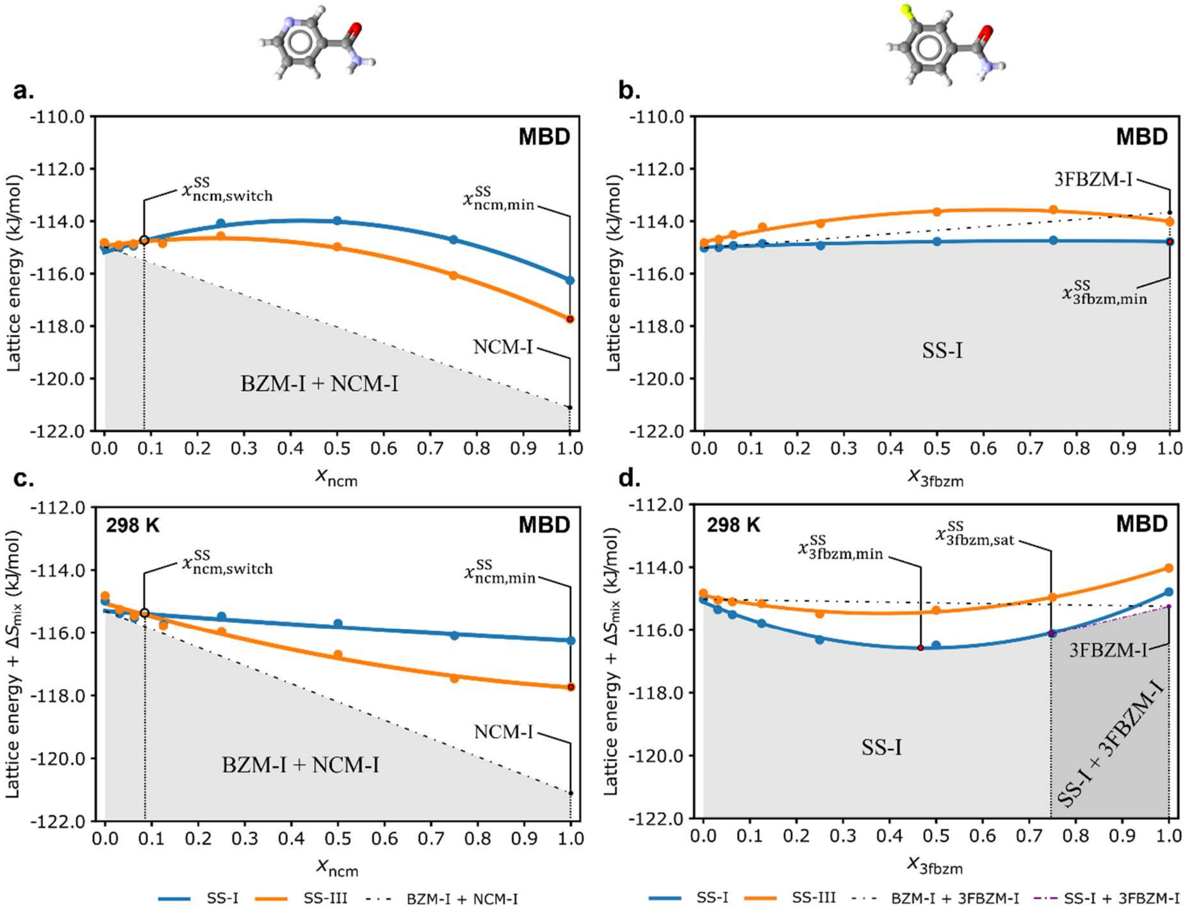

Supplementary Figure 3.2.1: The impact of adding the entropy of mixing ($-T\Delta S_{\mathrm{mix}}$ at 298 K) on the lattice energies of SSs incorporating lowest energy conformers - ncm$_B$ (a and c) and 3fbzm$_A$ (BZM-I) and 3fbzm$_B$ (BZM-III) (b and d) incorporating into the BZM-I (blue) and BZM-III (orange) lattices. Ideal mixing is assumed, with only one type of conformer incorporating (i.e. a 2-component SS). The entropy of mixing term reaches a maximum at x_g = 0.5 ($-T\Delta S_{\mathrm{mix}}$ = 1.72 kJ / mol). The same corrective factor is added to each structure, therefore no change in crossover concentrations occurs, the SS structures are simply stabilised relative to the pure components. Physical mixtures energies of the two stable pure components (BZM-I + NCM-I and BZM-I + 3FBZM-I) in each system are shown by black dash-dotted lines, while the minimum energy SS ($x^{\mathrm{SS}}_{\mathrm{g,min}}$) is shown by a red point. Energies of the pure guest structures (NCM-I and 3FBZM-I) are labelled, as well as $x^{\mathrm{SS}}_{\mathrm{ncm,switch}}$ and $x^{\mathrm{SS}}_{\mathrm{3fbzm,sat}}$. This model incorrectly predicts a saturation point ($x^{\mathrm{SS}}_{\mathrm{3fbzm,sat}}$) for the bzm:3fbzm system, shown by a purple dash-dotted line. Phase boundaries are labelled and shaded with the lowest energy phase or mixture expected to crystallise. SS-I denotes a SS with the BZM-I structure, while SS-I + 3FBZM-I denotes a mixture of BZM-I-SS at $x^{\mathrm{SS}}_{\mathrm{3fbzm,sat}}$ with excess 3FBZM-I.

3.3 Impact of Disorder

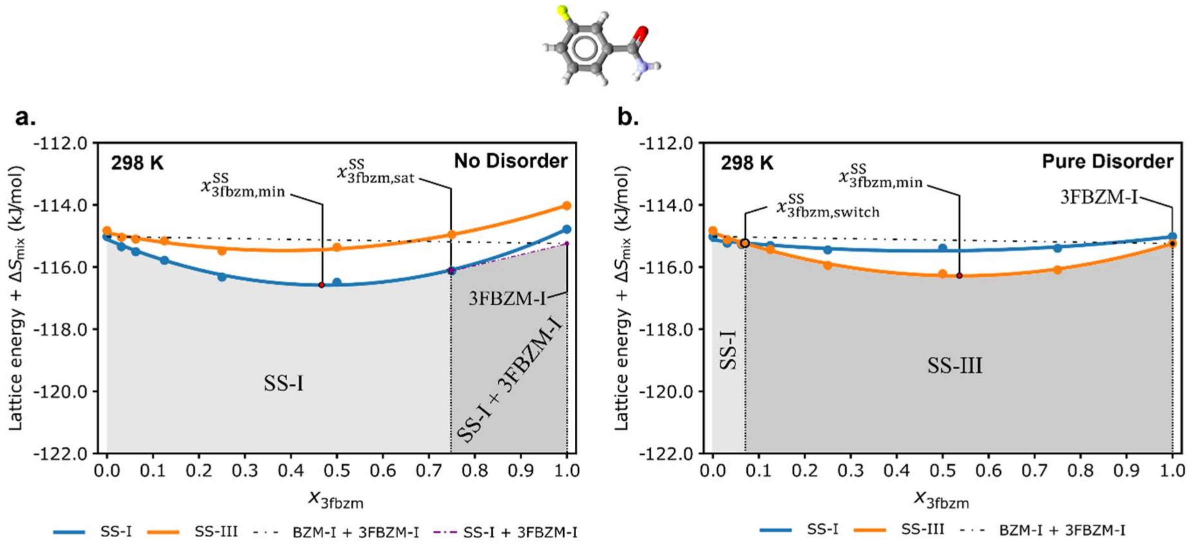

Supplementary Figure 3.3.1: A comparison between the bzm:3fbzm system without conformational disorder (a) and including conformational disorder corresponding to the conformer ratio in the pure 3FBZM-I system - 0.331:0.669 | A:B (b). SSs with the BZM-I structure are shown in blue, while SSs with the BZM-III structure are shown in orange. Physical mixtures energies of the two stable pure components (BZM-I + NCM-I and BZM-I + 3FBZM-I) in each system are shown by black dash-dotted lines, a mixture of BZM-I-SS and excess 3FBZM-I is shown by a purple dash-dotted line, while the minimum energy SS ($x^{SS}_{g,min}$) is shown by a red point. The energy of the pure 3FBZM-I is labelled, as well as $x^{SS}_{3fbzm,switch}$ and $x^{SS}_{3fbzm,sat}$. Phase boundaries are labelled and shaded with the lowest energy phase or mixture expected to crystallise. SS-I and SS-III denotes a SS with the BZM-I and BZM-III structures, respectively, while SS-I + 3FBZM-I denotes a mixture of BZM-I-SS at $x^{SS}_{3fbzm,sat}$ with excess 3FBZM-I.

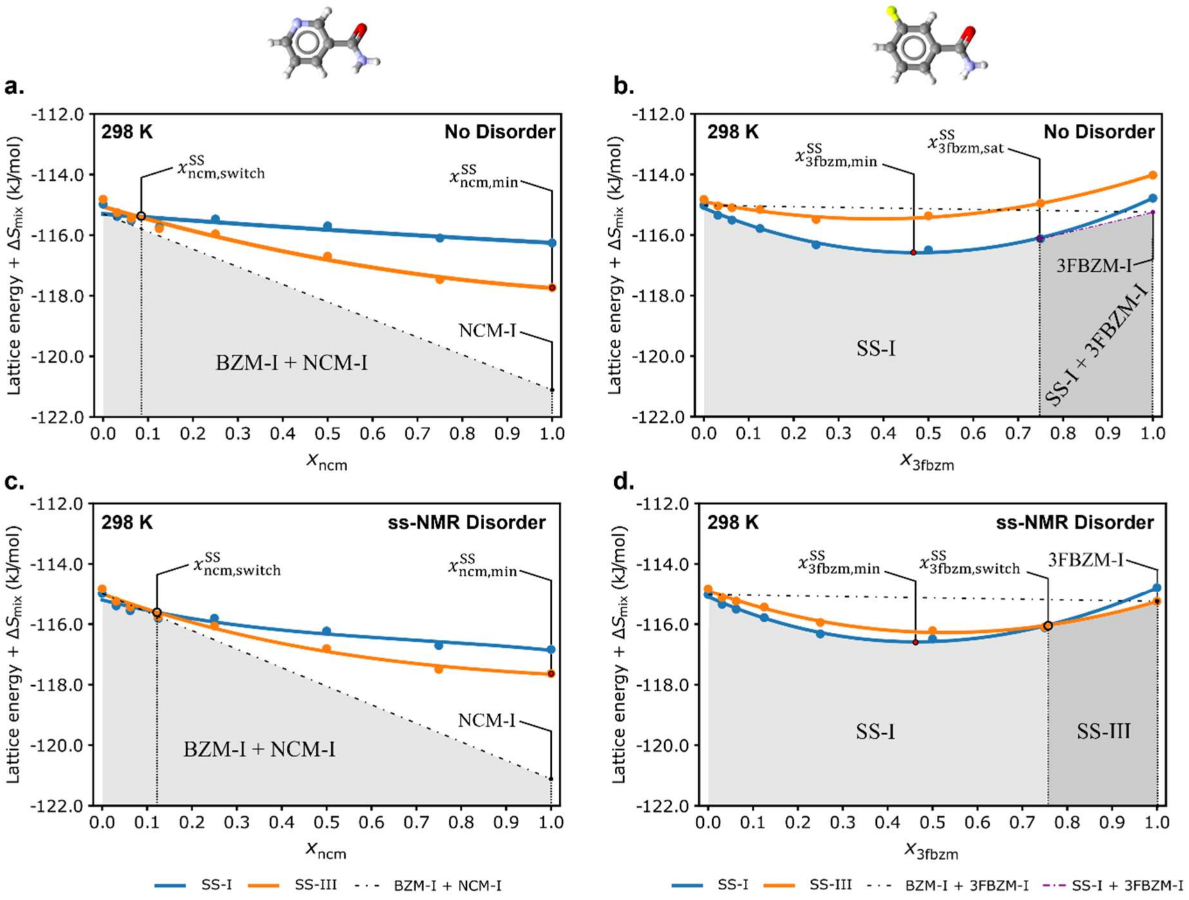

Supplementary Figure 3.3.2: The impact of adding disorder and entropy of mixing ($-T\Delta S_{\text{mix}}$ at 298 K) on the lattice energies of bzm:ncm (c) and bzm:3fbzm (d) incorporating into the BZM lattices with conformational disorder, with the conformationally ordered structures for bzm:ncm and bzm:3fbzm shown in (a) and (b), respectively. Both conformers of ncm and 3fbzm were modelled to incorporate in the bzm lattices, with the ratios informed from the ssNMR experiments. In the bzm:ncm system, the conformer ratio used was 0.2:0.8 | ncm_A:ncm_B for both BZM-I and BZM-III, while in the bzm:3fbzm system the conformer ratio used was 1:0 | $3fbzm_A$:$3fbzm_B$ in BZM-I and 0.331:0.669 | $3fbzm_A$:$3fbzm_B$ in BZM-III. SSs with the BZM-I structure are shown in blue, while SSs with the BZM-III structure are shown in orange. Physical mixtures energies of the two stable pure components (BZM-I + NCM-I and BZM-I + 3FBZM-I) in each system are shown by black dash-dotted lines, while the minimum energy SS ($x^{\text{SS}}_{\text{g,min}}$) is shown by a red point. Energies of the pure guest structures (NCM-I and 3FBZM-I) are labelled, as well as $x^{\text{SS}}_{\text{g,switch}}$. Phase boundaries are labelled and shaded with the lowest energy phase or mixture expected to crystallise exclusively. SS-I and SS-III denote a SS with the BZM-I and BZM-III structures, respectively, while SS-I + 3FBZM-I denotes a mixture of BZM-I-SS at $x^{\text{SS}}_{\text{3fbzm,sat}}$ with excess 3FBZM-I.

3.4 Free Energy vs. Physical Mixtures – A Detailed Discussion

Crystallisation of a host and a guest under different stoichiometries and solvent conditions may result in several possibilities. A SS is competing in formation and stability against several other solid forms including: a mixture of forms of the pure host and guest, a host:guest cocrystal or cocrystals with various stoichiometries, a mixture of cocrystal and an excess of forms of pure host and guest, a solvate or hydrate, a SS adopting an alternative crystal structure, a mixture of multiple SSs of varying $x_{\mathrm{g}}^{\mathrm{SS}}$, a mixture of a SS at a higher or lower $x_{\mathrm{g}}^{\mathrm{SS}}$ value with an excess of one phase of pure guest or host, and any combination of the above.

For a SS to be the most stable form, its free energy must be the lowest overall when compared to the free energy of the other competing systems at a given value of $x_{\mathrm{g}}^{\mathrm{SS}}$ (or the general term x_{g}, also encompassing mixtures). The systems studied in this work did not form host:guest cocrystals or solvates, therefore they will not be discussed any further here – however their formation is always a possibility. Instead, we compare here the stability of SSs relative to the pure host and guest solids or the SS with excess of pure guest or host.

Comparisons of the free energy of a SS to the free energies of physical mixtures of the pure host or the SS crystallising with excess host or guest can be used to predict if a SS is continuous or partial in nature and predict a value of $x_{\mathrm{g,sat}}^{\mathrm{SS}}$. Comparing the free energies of multiple SSs may result in a value for $x_{\mathrm{g,switch}}^{\mathrm{SS}}$ if the free energies of the structures cross, and thus the stability of the polymorphic SSs can switch at different concentrations. The free energies of mixtures of phases can be calculated by simple extrapolation from $G_{\mathrm{g}}^{\mathrm{SS}}\left[x_{\mathrm{g}}^{\mathrm{SS}}\right]$ between the phases involved. For example, the free energy of the mixture of pure host and pure guest can be plotted by drawing the line between the free energy of the pure host most stable crystal structure at x_{g}=0 and the free energy of the pure guest most stable crystal structure at x_{g}=1. Energies for mixtures of different SSs can also be calculated by connecting the energies of the two SSs at their respective $x_{\mathrm{g}}^{\mathrm{SS}}$ values, then extrapolating to either pure component in excess, depending on $x_{\mathrm{g}}^{\mathrm{SS}}$ or x_{g}. Mixture lines can be drawn using an identical method to the continuous SS system, however there will be a point where a mixture of a SS and excess pure component deviates from the SS energy and becomes more stable. This point can be found by calculating the gradient of the SS line between each x_{g} value and comparing this to the gradient of the mixture of the SS with the pure component in excess above x_{g}. Once the gradient of the SS energy is less steep than the mixture line, $x_{\mathrm{g,sat}}^{\mathrm{SS}}$ has been reached, and the lowest energy system

at each x_g above this value will be a mixture of the SS at $x_{g,sat}^{SS}$ and an excess of the lowest energy pure component.

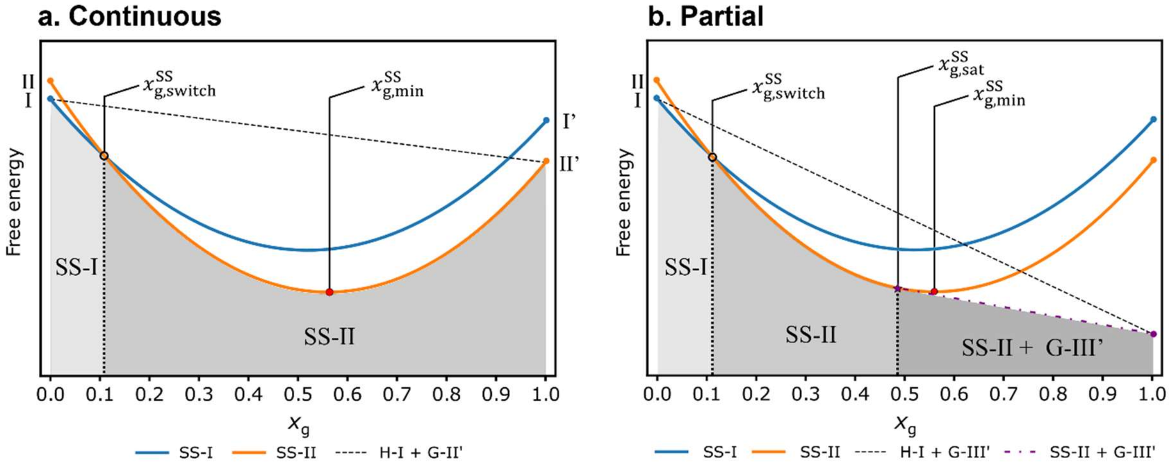

Supplementary Figure 3.4.1: Plots of free energy (G_g^{SS}) vs. x_g for two thermodynamically stable SS systems composed of host "H" and guest "G", where two polymorphic SSs are competing as a continuous SS (a) and a partial SS (b). Both plots show the free energies of a SS with the structure of the initially stable form I (blue) and initially metastable form II (orange) of the host compound. The free energy of the mixture of the two pure components of the SS is shown by a dashed black line and the minimum energy SS is shown by a red point. A purple dash-dotted line (in b) denotes the energy of saturated SS-II mixed with excess of pure guest (G-III'), with $x_{g,sat}^{SS}$ marked by a star. Points I and II denote the energies of the pure host I and II structures, while I' and II' denote the energies of the same crystal structures with all molecules replaced by the guest species. The pure guest exists as II' in a and as III' in b. Phase boundaries are coloured and labelled according to the thermodynamically stable solid phase or mixture of phases.

Idealised examples of the free energy of two thermodynamically stable SSs are shown in Supplementary Figure 3.4.1, along with the energies of possible mixtures (dashed lines). Each SS adopts a crystal structure observed for the host molecule, either the known stable form (blue, SS-I), or an alternative known metastable form (orange, SS-II). In the examples, the pure guest exists in one crystal structure only (II' in a and III' in b). Supplementary Figure 3.4.1a illustrates the case for a continuous SS and Figure Supplementary Figure 3.4.1b illustrates the case for a partial SS.

In the example for the continuous system, one polymorph of the pure host (II) is isostructural with the pure guest crystal structure (II'). The free energies of the SSs adopting the structure of the host (I and II) remain lower than the free energy of the physical mixtures of the two pure components (black dashed line) across the entire range of guest composition. The free energies of the two SSs (SS-I and SS-II) cross at $x_{g,switch}^{SS}$~0.12. In this example, a SS would exist

across the entire compositional range (continuous) with SS-I being the stable form below 0.12 and SS-II being the stable form above 0.12.

In the example for the partial system, the crystal structure of the pure guest (III') is different to the two known polymorphs of the pure host (I and II'). Here, identical to the continuous system, the free energies of the two SSs (SS-I and SS-II) also cross at $x_{\mathrm{g,switch}}^{\mathrm{SS}}$~0.12 with SS-I being the stable form below 0.12 and SS-II being the stable form above 0.12. The most stable SS at different concentrations remains lower than the free energy of the physical mixtures of the two pure components (black dashed line) only up to a specific guest composition. And the free energy of the SS-II with excess of pure guest G-III' is even lower than the free energy of the pure physical mixture or the pure SS-II above $x_{\mathrm{g,sat}}^{\mathrm{SS}}$.

<u>Practical Example:</u>

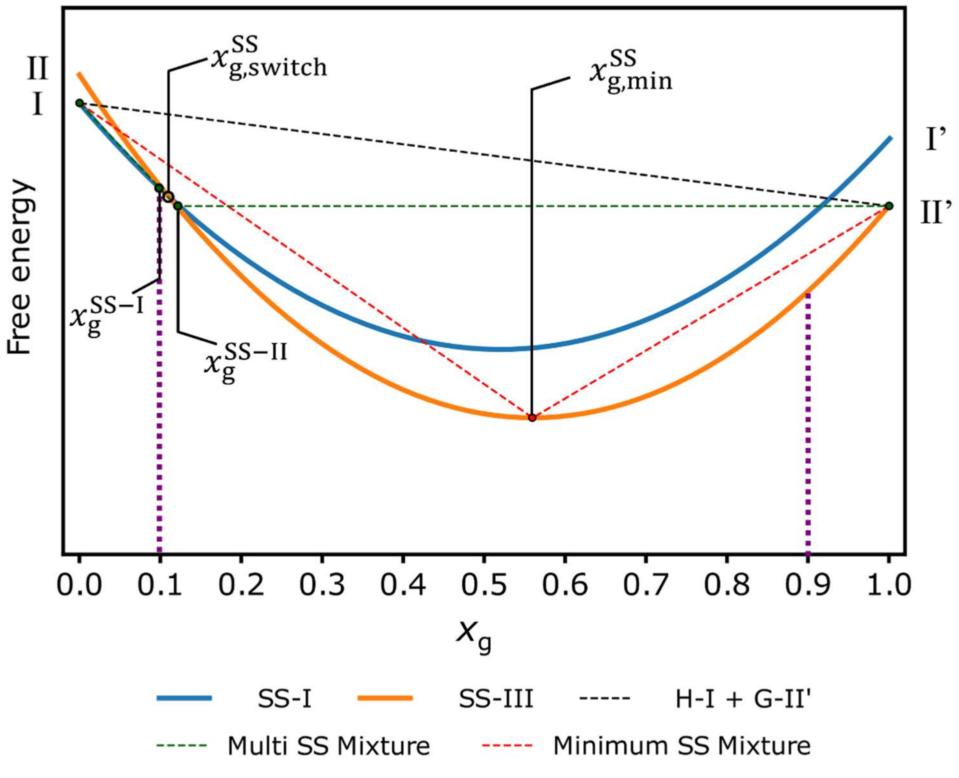

Supplementary Figure 3.4.2: Plot of free energy ($\Delta G_{\mathrm{g}}^{\mathrm{SS}}$) vs. x_{g} for a thermodynamically stable continuous SS system, where two polymorphic SSs are competing. The plot shows the free energy of a SS with the structure of the initially stable form-I (blue) and initially metastable form-II (orange) of the host compound. Mixtures are shown by dashed lines: two pure components (black); a mixture of pure components with the global minimum SS structure over the x_{g} range (red); and a mixture of two SSs plus a mixture of excess pure components (green). $x_{\mathrm{g,switch}}^{\mathrm{SS}}$ is marked as well as $x_{\mathrm{g,min}}^{\mathrm{SS}}$, and the two SSs that make up the mixture denoted by the green line $x_{\mathrm{g}}^{\mathrm{SS-I}}$ and $x_{\mathrm{g}}^{\mathrm{SS-II}}$ with their structures indicated by the superscript. Two purple dotted lines are also used to denote the two x_{g} values discussed in detail in this section, with the lines terminating when meeting the lowest energy phase in the system.

To practically demonstrate the complex mixing behaviour of a SS system, an example is discussed here. Focussing on the continuous SS system (ESI figure 3.4.2); two possible scenarios are presented. At $x_{\mathrm{g}} = 0.1$ (first dotted purple line):

i. 100% of the guest could mix in the host structure homogeneously across the system (producing a SS with $x_{\mathrm{g}}^{\mathrm{SS}} = 0.1$ exclusively, blue and orange lines); or
ii. a higher proportion of guest could mix heterogeneously with the host crystal structure and crystallise a SS with $x_{\mathrm{g}}^{\mathrm{SS}} > 0.1$ along with crystallising an excess of pure host.

One example of the latter scenario is shown by the red dashed line denoting the formation of the minimum energy SS across the x_{g} range (meeting the SS free energy at $x_{\mathrm{g,min}}^{\mathrm{SS}}$). If this minimum energy SS were to form at $x_{\mathrm{g}} = 0.1$, a fraction (x_{g} / $x_{\mathrm{g,min}}^{\mathrm{SS}}$) of the system would crystallise as the minimum energy SS and the remainder of the system would crystallise as pure host. As $x_{\mathrm{g}} < 0.5$, this would be an example of "guest-controlled" crystallisation of the SS, where the guest species would be consumed first. The free energy of this mixture is higher than free energy of crystallising either SS exclusively with $x_{\mathrm{g}}^{\mathrm{SS}} = 0.1$, and therefore will not form at $x_{\mathrm{g}} = 0.1$ under thermodynamic conditions.

Measuring the same mixture line at $x_{\mathrm{g}} = 0.9$ (second dashed purple line) would be an example of "host-controlled" crystallisation of the SS as $x_{\mathrm{g}} > 0.5$ ($x_{\mathrm{h}} < 0.5$). An equivalent proportion (x_{h} / $x_{\mathrm{h,min}}^{\mathrm{SS}}$) of the system would crystallise as the minimum energy SS ($x_{\mathrm{h,min}}^{\mathrm{SS}}$), with the remainder crystallising as pure guest. This is again higher in energy than any SS crystallising exclusively with $x_{\mathrm{g}}^{\mathrm{SS}} = 0.9$. For simplicity of discussion, host and guest here refer to the same respective components across the whole x_{g} range, despite this role technically switching at $x_{\mathrm{g}} = 0.5$. The free energy of the mixture line relative to the free energies of the SSs proves why, despite being the global minimum of the system, it is unfavourable to form the minimum energy SS at any x_{g} value aside from when $x_{\mathrm{g}} = x_{\mathrm{g,min}}^{\mathrm{SS}}$.

3.5 Disorder Ratio Calculations

All plotted disorder values on the below charts denote the mean distribution of conformers from all cell permutations with the same value of $x_{\mathrm{g}}^{\mathrm{SS}}$.

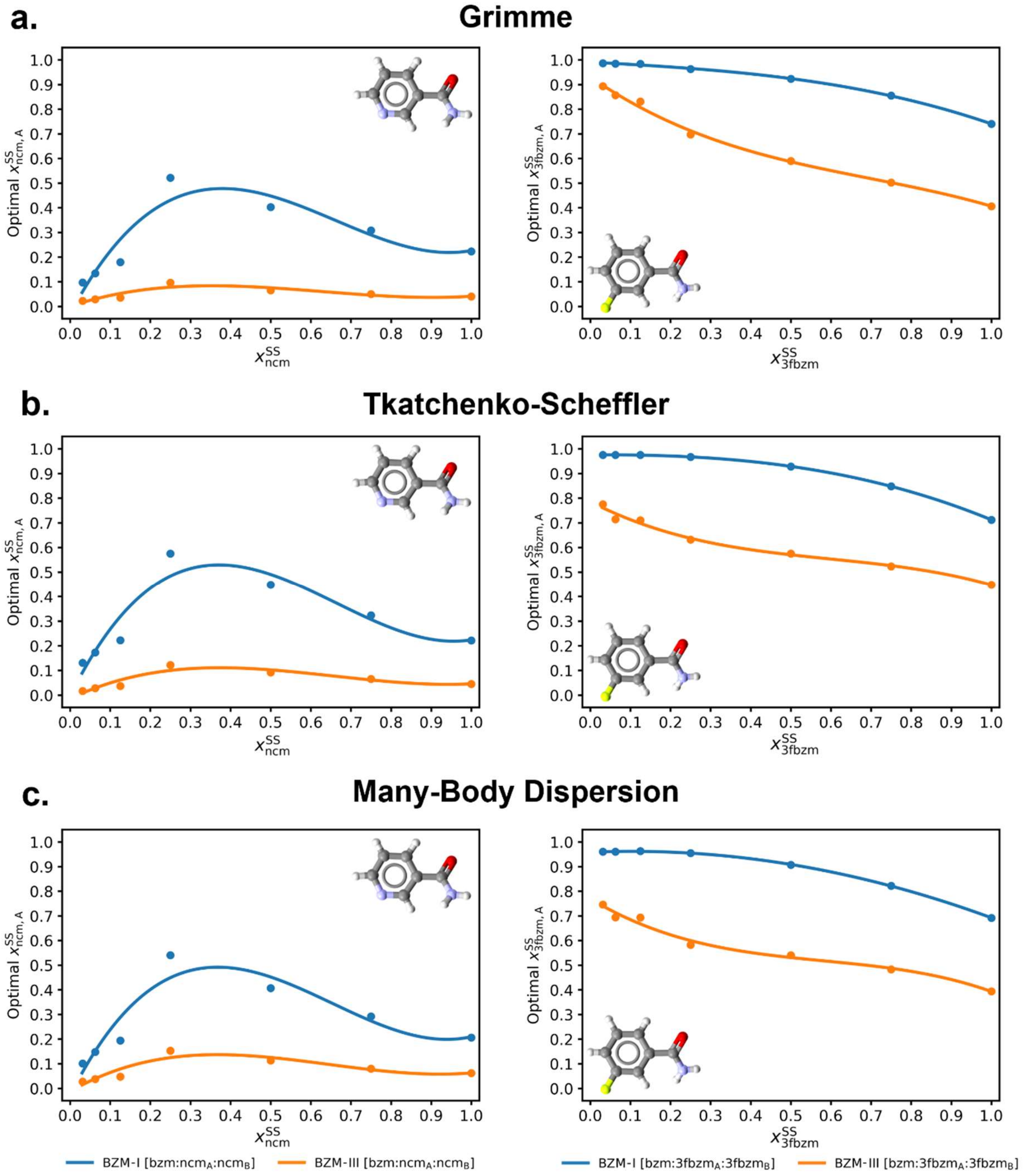

Supplementary Figure 3.5.1: The optimal disorder ratio in terms of guest conformer A predicted from the lowest energy value obtained from a weighted average of the energy for incorporating conformer A and conformer B (e.g. 0.5 x Energy A + 0.5 x Energy B). Left shows the bzm:ncm system and right shows the bzm:3fbzm system with different dispersion models: a – Grimme D2 corrections, b – Tkatchenko-Scheffler corrections and c – Many-body dispersion corrections. SSs with the BZM-I structure are shown in blue, while SSs with the BZM-III structure are shown in orange.

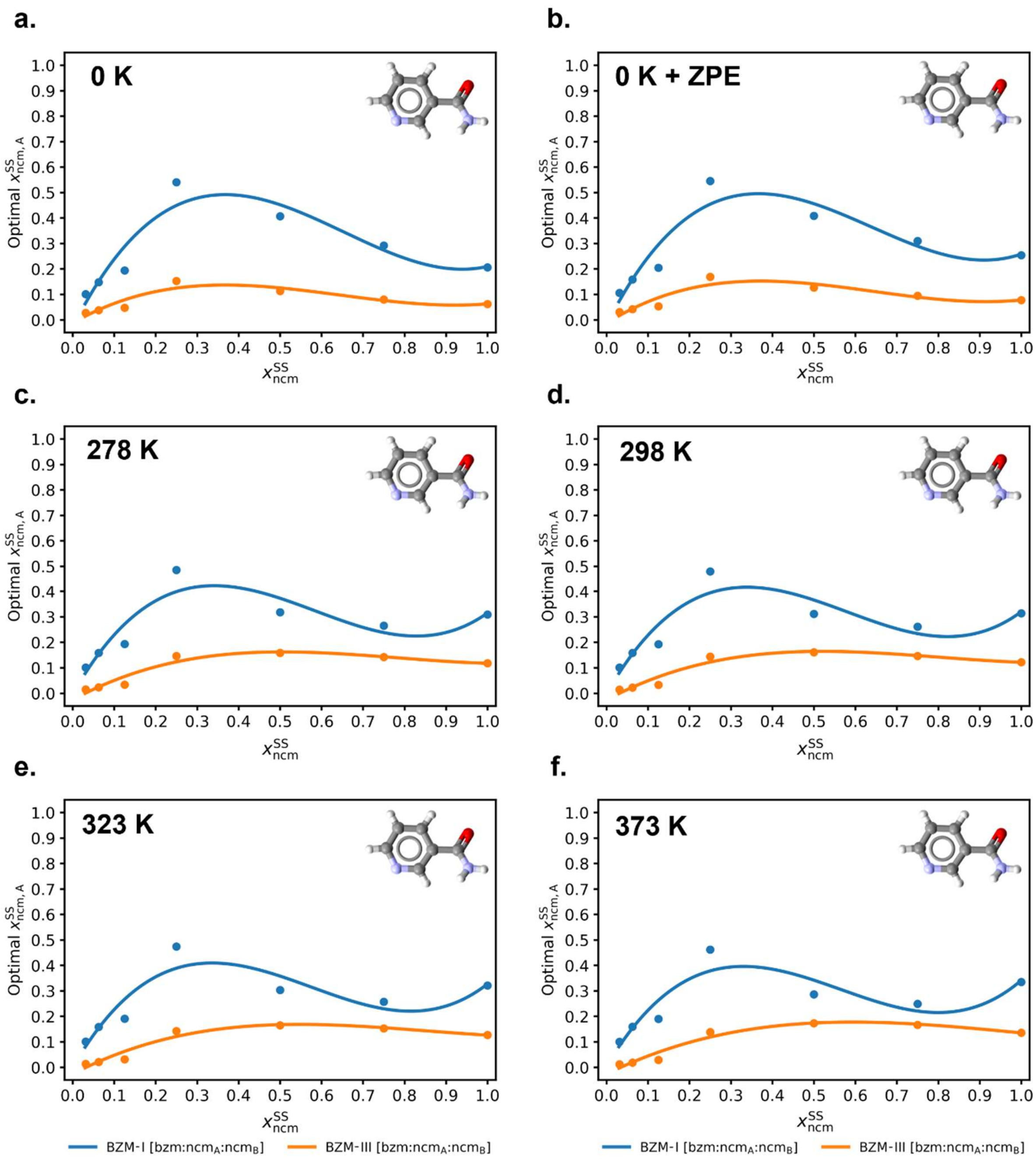

Supplementary Figure 3.5.2: The optimal conformer disorder ratio in terms of ncm$_A$ predicted for the bzm:ncm system, based on the cell producing the lowest energy at x^{SS}_{ncm}, starting from the MBD model (a), then adding zero-point energy (ZPE) corrections (b), followed by the addition of vibrational corrections (F_{vib}) to model four temperatures – 278 K, 398 K, 323 K and 373 K (c, d, e and f). SSs with the BZM-I structure are shown in blue, while SSs with the BZM-III structure are shown in orange.

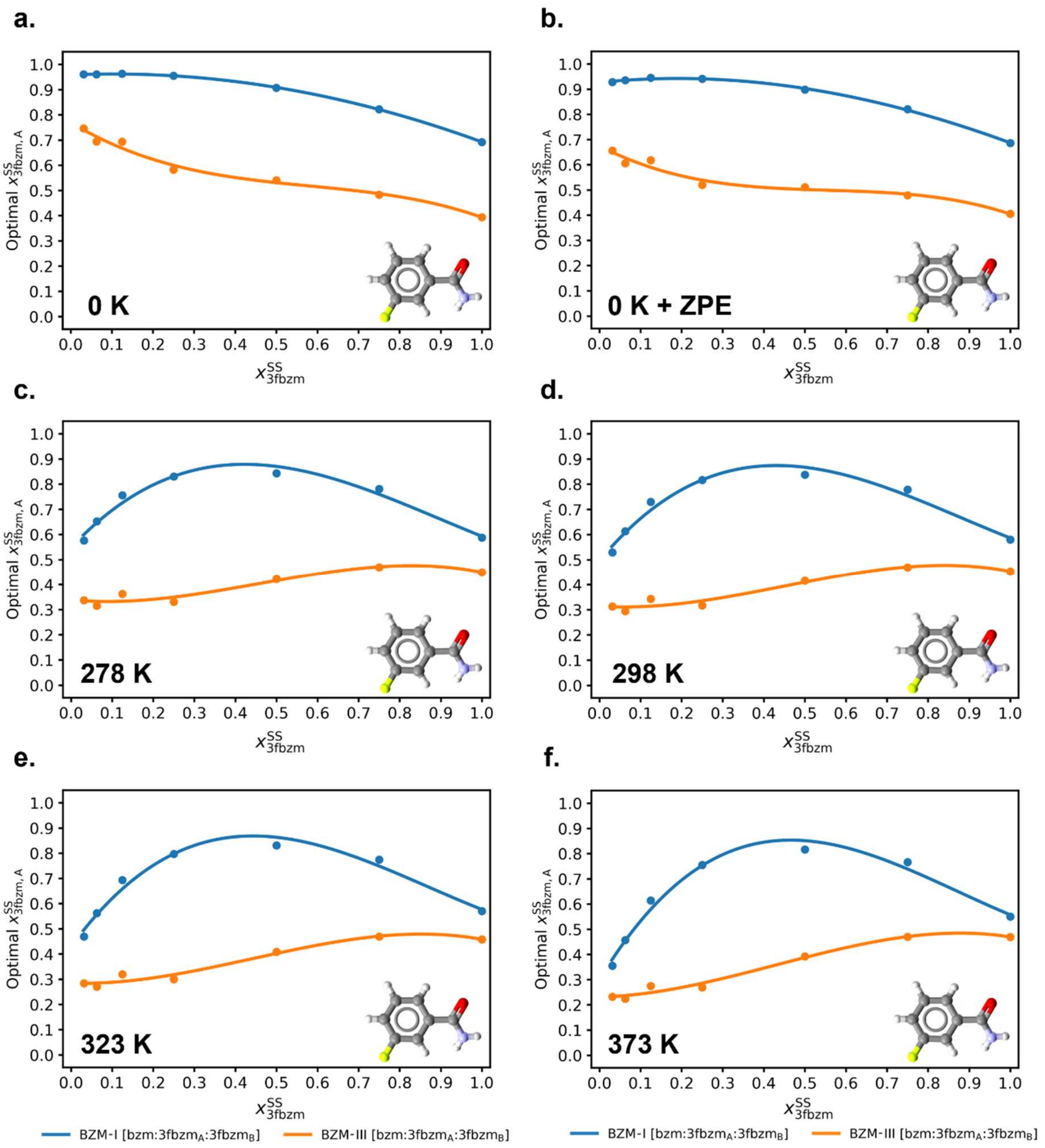

Supplementary Figure 3.5.3: The optimal conformer disorder ratio in terms of 3fbzm$_A$ predicted for the bzm:3fbzm system, based on the cell producing the lowest energy at x^{SS}_{3fbzm}, starting from the MBD model (a), then adding zero-point energy (ZPE) corrections (b), followed by the addition of vibrational corrections (F_{vib}) to model four temperatures – 278 K, 398 K, 323 K and 373 K (c, d, e and f). SSs with the BZM-I structure are shown in blue, while SSs with the BZM-III structure are shown in orange.

3.6 SS Energies with Error Bars

Here, figures from the main article are repeated with error bars included. Each plotted SS energy value is computed by taking the mean energy from all cell permutations with the same value of x_g^{SS}. Error bars are computed by calculating the standard error over the range of data (i.e. the standard deviation divided by the square root of the sample size).

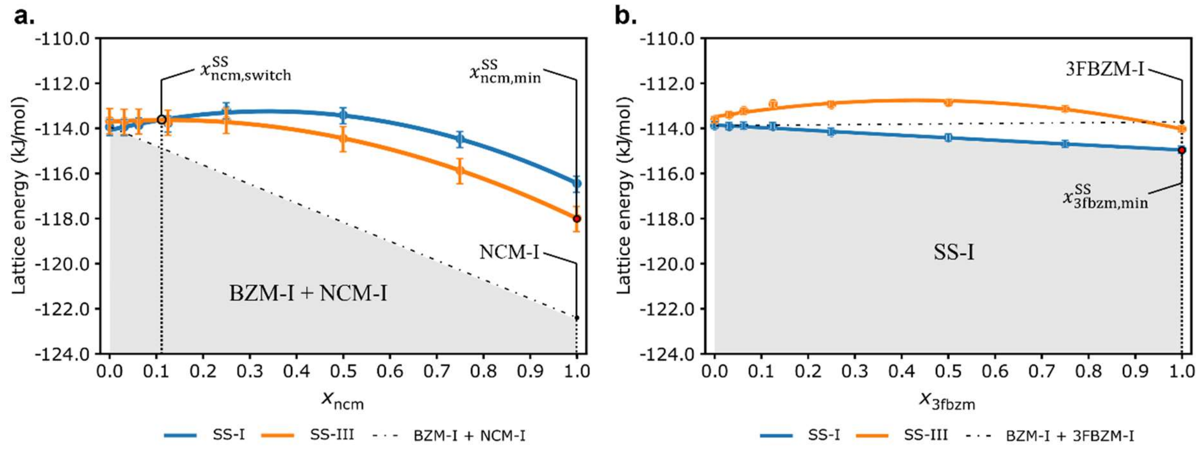

Supplementary Figure 3.6.1: Results from the original Grimme-D2 model for calculating SS energies for bzm:ncm (a) and bzm:3fbzm (b) with no mixing effects considered. Plots are a copy of figure 10 from the main manuscript with error bars plotted, denoting the standard error.

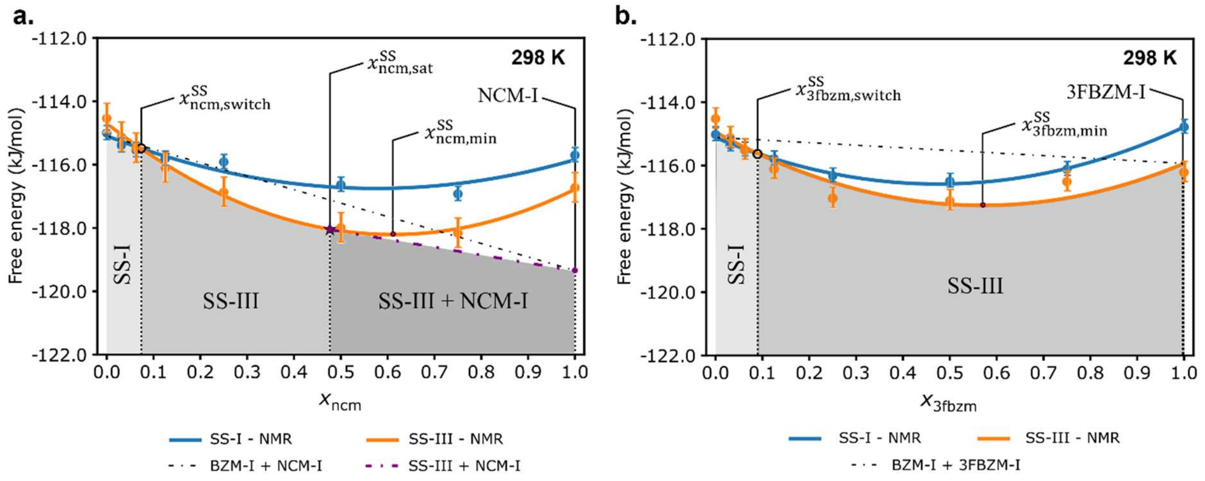

Supplementary Figure 3.6.2: Results from the improved model with ssNMR measured conformational disorder values for bzm:ncm (a) and bzm:3fbzm (b) at 298 K. Plots are a copy of figure 11 from the main manuscript with error bars plotted, denoting the standard error.

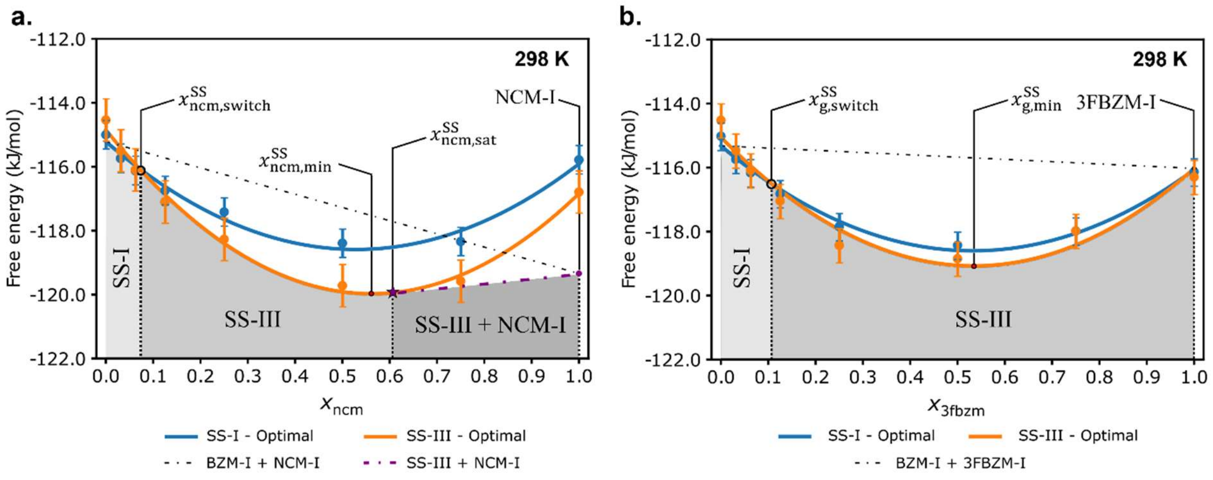

Supplementary Figure 3.6.3: Free energies obtained using the final model with predicted optimal disorder values for bzm:ncm (a) and bzm:3fbzm (b) at 298 K. Plots are a copy of figure 12 from the main manuscript with error bars plotted, denoting the standard error.

3.7 Studies of Molecular ncm and 3fbzm

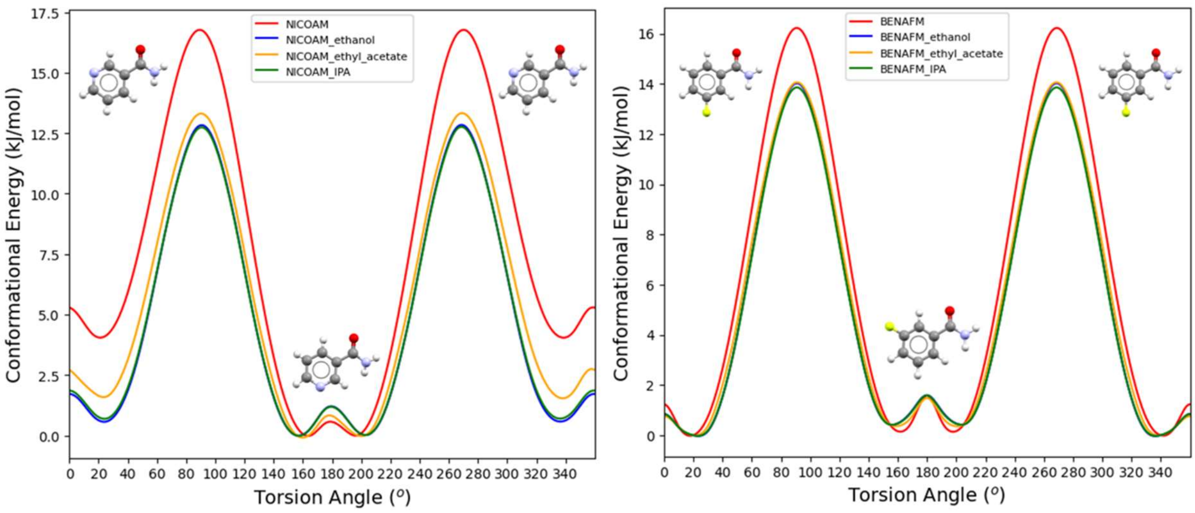

Supplementary Figure 3.7.1: Conformational energy plots for ncm (left) and 3fbzm (right). Conformational scans were performed with Gaussian16, using the M06 functional with the 6-31+G** basis sets. Solvent models were added using the predefined SMD solvent models. Torsion scans performed in vacuum are shown in red, ethanol in blue, ethyl acetate in yellow and IPA in green.

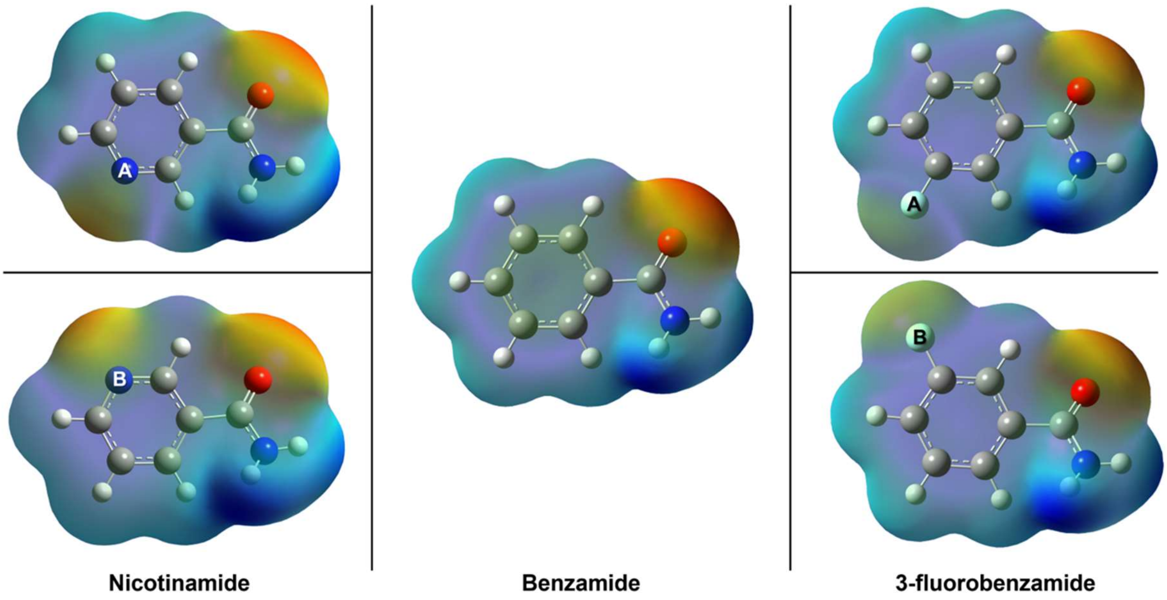

Supplementary Figure 3.7.2: ESP charge maps of benzamide (centre) and related molecules nicotinamide (left) and 3-fluorobenzamide (right). Both conformer A (top) and B (bottom) are shown. The red area denotes areas of more negative charge, whereas the blue areas denote areas of more positive charge. ESP charge maps were calculated by optimising the molecular geometries at the M06/6-31+G** level of theory.

Supplementary References

1 W. Kras, A. Carletta, R. Montis, R. A. Sullivan and A. J. Cruz-Cabeza, Switching polymorph stabilities with impurities provides a thermodynamic route to benzamide form III, *Commun Chem*, 2021, **4**, 38.
2 C. R. Groom, I. J. Bruno, M. P. Lightfoot and S. C. Ward, The Cambridge Structural Database, *Acta Crystallographica Section B*, 2016, **72**, 171–179.
3 K. Kobayashi, A. Sato, S. Sakamoto and K. Yamaguchi, Solvent-Induced Polymorphism of Three-Dimensional Hydrogen-Bonded Networks of Hexakis(4-carbamoylphenyl)benzene, *J Am Chem Soc*, 2003, **125**, 3035–3045.
4 J. Thun, L. Seyfarth, C. Butterhof, J. Senker, R. E. Dinnebier and J. Breu, Wöhler and Liebig Revisited: 176 Years of Polymorphism in Benzamide - and the Story Still Continues!, *Cryst Growth Des*, 2009, **9**, 2435–2441.
5 K. E. Johansson and J. van de Streek, Revision of the Crystal Structure of the First Molecular Polymorph in History, *Cryst Growth Des*, 2016, **16**, 1366–1370.
6 G. Kresse and J. Hafner, Ab initio molecular dynamics for liquid metals, *Phys Rev B*, 1993, **47**, 558–561.
7 G. Kresse and J. Hafner, Ab initio molecular-dynamics simulation of the liquid-metal--amorphous-semiconductor transition in germanium, *Phys Rev B*, 1994, **49**, 14251–14269.
8 G. Kresse and J. Furthmüller, Efficiency of ab-initio total energy calculations for metals and semiconductors using a plane-wave basis set, *Comput Mater Sci*, 1996, **6**, 15–50.
9 G. Kresse and J. Furthmüller, Efficient iterative schemes for ab initio total-energy calculations using a plane-wave basis set, *Phys Rev B*, 1996, **54**, 11169–11186.
10 S. Grimme, Semiempirical GGA-type density functional constructed with a long-range dispersion correction, *J Comput Chem*, 2006, **27**, 1787–1799.
11 P. E. Blöchl, Projector augmented-wave method, *Phys Rev B*, 1994, **50**, 17953–17979.
12 G. Kresse and D. Joubert, From ultrasoft pseudopotentials to the projector augmented-wave method, *Phys Rev B*, 1999, **59**, 1758–1775.
13 A. Tkatchenko and M. Scheffler, Accurate Molecular Van Der Waals Interactions from Ground-State Electron Density and Free-Atom Reference Data, *Phys Rev Lett*, 2009, **102**, 73005.
14 A. Tkatchenko, R. A. DiStasio, R. Car and M. Scheffler, Accurate and Efficient Method for Many-Body van der Waals Interactions, *Phys Rev Lett*, 2012, **108**, 236402.
15 A. Ambrosetti, A. M. Reilly, R. A. DiStasio and A. Tkatchenko, Long-range correlation energy calculated from coupled atomic response functions, *J Chem Phys*, 2014, **140**, 18A508.
16 T. Bučko, S. Lebègue, T. Gould and J. G. Ángyán, Many-body dispersion corrections for periodic systems: an efficient reciprocal space implementation, *Journal of Physics: Condensed Matter*, 2016, **28**, 45201.
17 H. J. Monkhorst and J. D. Pack, Special points for Brillouin-zone integrations, *Phys Rev B*, 1976, **13**, 5188–5192.
18 H. Johannes, K. Hsin-Yu, N. M. A., C. Roberto, D. R. A. and T. Alexandre, Reliable and practical computational description of molecular crystal polymorphs, *Sci Adv*, 2019, **5**, eaau3338.
19 J. Nyman and G. M. Day, Static and lattice vibrational energy differences between polymorphs, *CrystEngComm*, 2015, **17**, 5154–5165.
20 M. R. Willcott, MestRe Nova, *J Am Chem Soc*, 2009, **131**, 13180.
21 Agilent Technologies Ltd, 2017.

22 O. V Dolomanov, L. J. Bourhis, R. J. Gildea, J. A. K. Howard and H. Puschmann, OLEX2: a complete structure solution, refinement and analysis program, *J Appl Crystallogr*, 2009, **42**, 339–341.
23 G. M. Sheldrick, A short history of SHELX, *Acta Crystallographica Section A*, 2008, **64**, 112–122.
24 G. M. Sheldrick, Crystal structure refinement with SHELXL, *Acta Crystallographica Section C*, 2015, **71**, 3–8.
25 E. Prince and C. H. Spiegelman, in *International Tables for Crystallography*, Kluwer Academic Publishers, Dordrecht, 1992, vol. C, pp. 622–624.
